# Efficacy and safety of *Melaleuca alternifolia* (tea tree) oil for human health—A systematic review of randomized controlled trials

**DOI:** 10.3389/fphar.2023.1116077

**Published:** 2023-03-24

**Authors:** Lana Kairey, Tamara Agnew, Esther Joy Bowles, Bronwyn J. Barkla, Jon Wardle, Romy Lauche

**Affiliations:** ^1^ National Centre for Naturopathic Medicine, Southern Cross University, Lismore, NSW, Australia; ^2^ Southern Cross Plant Science, Faculty of Science and Engineering, Southern Cross University, Lismore, NSW, Australia

**Keywords:** tea tree oil, *Melaleuca alternifolia* oil, essential oils, phytotherapy, systematic review, randomized controlled trials, anti-infective agents, human trial

## Abstract

**Introduction:** Leaves of the Australian tea tree plant *Melaleuca alternifolia* were used traditionally by First Nations Australians for treating wounds, burns, and insect bites. Tea tree oil, the essential oil steam-distilled from *M. alternifolia*, is well-known for its medicinal properties, the evidence for most applications however is limited. This review aimed to critically appraise evidence from clinical trials examining the therapeutic efficacy and safety of tea tree oil on outcomes.

**Methods:** Randomized controlled trials with participants of any age, gender, or health status, comparing tea tree oil to any control were included, without limit on publication date. Electronic databases were searched on 12 August 2022 with additional records sourced from article reference sections, reviews, and industry white papers. Risk of bias was assessed by two authors independently using the Cochrane risk-of-bias 1.0 tool. Results were summarized and synthesized thematically.

**Results:** Forty-six articles were eligible from the following medical fields (*Dentistry n* = 18, *Dermatology n* = 9, *Infectious disease n* = 9, *Ophthalmology n* = 6, *Podiatry n* = 3; and *Other n* = 1). Results indicate that oral mouthwashes with 0.2%–0.5% tea tree oil may limit accumulation of dental plaque. Gels containing 5% tea tree oil applied directly to the periodontium may aid treatment of periodontitis as an adjunctive therapy to scaling and root planing. More evidence is needed to confirm the benefits of tea tree oil for reducing acne lesions and severity. Local anti-inflammatory effects on skin, if any, also require further elucidation. Topical tea tree oil regimens show similar efficacy to standard treatments for decolonizing the body from methicillin-resistant *Staphylococcus aureus*, although intra-nasal use of tea tree oil may cause irritation to mucous membranes. Tea tree oil with added iodine may provide an effective treatment for *molluscum contagiosum* lesions in young children. More evidence on efficacy of tea tree oil-based eyelid wipes for *Demodex* mite control are needed. Side effects were reported in 60% of included studies and were minor, except where tea tree oil was applied topically in concentrations ≥ 25%.

**Discussion:** Overall, the quality of research was poor to modest and higher quality trials with larger samples and better reporting are required to substantiate potential therapeutic applications of tea tree oil.

**Systematic Review Registration:** PROSPERO, identifier [CRD42021285168].

## 1 Introduction

Tea tree oil is the essential oil derived from the *M. alternifolia* (Maiden & Betche) Cheel plant, an Australian native plant endemic to north-eastern New South Wales and Southern Queensland (Carson et al., 2006; Montreal Process Implementation Group for Australia and National Forest Inventory Steering Committee, 2019). While the whole above-ground structure is harvested and turned into biomass, only the leaves contribute to the constituents in tea tree oil, which are extracted by steam distillation. The resulting essential oil has a unique and distinct medicinal, camphoraceous odor (Rhind, 2020). The oil of *M. alternifolia* (Maiden & Betche) Cheel contains more than 100 components. For the purpose of assessing the quality of manufactured tea tree oil, the International Organization for Standardization has defined minimum and maximum concentrations for 15 of these components, with the primary active constituent being terpinen-4-ol, comprising 35%–48% of tea tree oil (International Organization for Standardization, 2017).

While tea tree oil may be manufactured from other species of the *Melaleuca* genus, *M. alternifolia* (Maiden & Betche) Cheel accounts for nearly 100% of tea tree oil manufactured worldwide and is therefore the most common form of tea tree oil available for purchase. Tea tree oil may be purchased over the counter or online as pure essential oil (100%), retailing for around $2-5 USD per 10 ml, or diluted to 5%–15% in a carrier oil. The majority of tea tree oil production occurs in Australia, although China, South Africa, Zimbabwe and Kenya also produce tea tree oil for commercial sale (Thomas and Deshmukh, 2019). In 2021, Australia account for 81% of the global production for steam-distilled, ISO4730:2017 compliant tea tree oil. Tea tree oil is used in healthcare/household, cosmetic, pharmaceutical, and aromatherapy products. Given the antimicrobial, anti-inflammatory and analgesic properties of tea tree oil and relative safety for topical use, tea tree oil is increasingly being used in cosmetic and pharmaceutical products (e.g., shampoos, soaps and liquid body wash, mouth washes, as well as over-the-counter treatments for cold sores, acne, burns, bites, lice, and fungal nail infections) (Thomas and Deshmukh, 2019; Rhind, 2020).

First Nations Australians have a long oral history of using the tea tree plant for medicinal purposes, of which they still practice today (Carson et al., 2006). The earliest written record of its use by First Nations Australians was the Bundjalung people of northern New South Wales who use it to treat wounds, burns, insect bites and upper respiratory infections (Braun et al., 2005; Carson et al., 2006). Arthur Penfold described the medicinal value of tea tree essential oil in 1925 (Carson et al., 2006), although the first official Australian report of its use in western medicine was in a 1930 article published in the Medical Journal of Australia (MJA) where it was described as having ‘impressive wound healing and antiseptic qualities’ (Murray, 1992; Braun et al., 2005). According to the European Medical Agency (EMA) monograph, tea tree oil has a well-established use as a traditional herbal medicinal product for 1) the treatment of small superficial wounds and insect bites, 2) the treatment of small boils (furuncles and mild acne), 3) relief of itching and irritation in cases of mild athlete’s foot, and 4) symptomatic treatment of minor inflammation of the oral mucosa, based upon its’ long-standing use for these indications (European Medical Agency, 2012). Similarly, the World Health Organization (1999) monograph on tea tree oil states the primary evidence-based therapeutic indications include symptomatic treatment of common skin disorders (e.g., acne, tinea pedis, furunculosis, and onychomycosis), as well as vaginitis (World Health Organization, 1999). However, the use of tea tree oil for oral inflammatory conditions including gingivitis, stomatitis, and tonsillitis, is described as folk medicine due to a lack of experimental or clinical data (World Health Organization, 1999). Further, due to a lack of safety data, the use of tea tree oil is contraindicated in those with hypersensitivity to the active substance or colophony, those pregnant, lactating, or trying to conceive, or aged < 12 years (International Organization for Standardization, 2017). While tea tree oil is considered safe for topical application at concentrations < 15% (Tisserand and Young, 2014), application *via* oral, ocular, otic or inhalation routes is not recommended (International Organization for Standardization, 2017).

Studies have reported positive benefits of tea tree oil against a range of bacteria, fungi and protozoa (Carson et al., 2006; Deyno et al., 2019). *In vitro* evidence has demonstrated tea tree oil to have broad spectrum antimicrobial activity, as well as anti-fungal and anti-viral actions, and to increase peripheral blood flow (Carson et al., 2006; Rhind, 2020). Tea tree oil has been proposed to alter the integrity and permeability of the cell wall of the bacteria, inhibit cell respiration, and alter the ability to of cells to maintain homeostatic conditions, inhibiting functions related to cell growth and replication (Carson et al., 2006).

Despite having an established historical and traditional use for selected therapeutic indications, supported by *in vitro* data, clinical human trials investigating the efficacy and safety of tea tree oil are required to permit evidence-based treatment advice. Previous systematic reviews have been limited to demodectic conditions and periodontitis (Casarin et al., 2018; Lam et al., 2020; Savla et al., 2020). Further, in 2000 Ernst and Huntley reviewed randomized clinical trials on topically applied tea tree oil for dermatological conditions (Ernst and Huntley, 2000). To date no comprehensive systematic review on the efficacy and safety of tea tree oil for human therapeutic use has been conducted. The aim of this review, therefore, was to critically appraise evidence from human trials (i.e., randomized control trials; RCTs) testing the therapeutic efficacy and/or safety of tea tree oil on any outcome(s) related to human health.

## 2 Materials and methods

The protocol for this review was registered prospectively with the International Prospective Register of Systematic Reviews (PROSPERO) CRD42021285168. The research was funded by AgriFutures Australia (Grant No. PRJ-012616). Reporting of this systematic review is in accordance with the Preferred Reporting Items for Systematic Reviews and Meta-Analyses (PRISMA) statement (Page et al., 2021).

### 2.1 Eligibility criteria

We included randomized controlled trials (RCTs) testing the effect of tea tree oil on any health-related outcome in human participants. Participants of any age, gender and health status were included. Interventions included tea tree oil from the species *M. alternifolia* present in any dilution and within any type of carrier (e.g., gels, creams, salves). We excluded interventions testing tea tree oil within a product containing other active ingredients known to have the same anticipated effect(s) as tea tree oil, e.g., an herbal shampoo with other essential oils, as the effect of tea tree oil was not studied in isolation. We also excluded interventions using tea tree oil in combination with other therapies unless these therapies were present in both intervention and comparison groups (i.e., co-interventions). Comparison groups eligible for inclusion were inactive controls (i.e., placebo, no treatment, wait list, usual or standard care) or an active control testing the same product without tea tree oil added or testing some variation of the tea tree oil intervention (e.g., different concentration, form, or dosage). Eligible studies measured the effect of tea tree oil on outcomes related to human health or disease including mortality, physiological (clinical) measures, quality of life and functional capacity, and safety outcomes such as toxicity or adverse reactions. Both self-reported and objectively measured outcomes were included. We included all types of RCTs, i.e., parallel, cluster, factorial, cross-over, as well as “split-body” and “split-mouth” designs where different treatments were applied to separate parts of the body or mouth, respectively, provided allocation was randomized, e.g., treatments allocated at random to right and left hands. Furthermore, we limited publication type to peer reviewed journal articles and included articles published in languages in which the review authors were fluent (i.e., English, German and Russian) which enabled an accurate translation of the text to be obtained within available resources.

### 2.2 Information sources, searches, and selection

Electronic databases PubMed, Scopus, and the Cochrane Central Register of Controlled Trials (CENTRAL) were searched on 12 August 2022 using pre-defined search strategies (see Supplementary Material). No limit regarding the publication date was applied. Titles and abstracts of all records from electronic database searches were exported to Endnote X9 (Clarivate Analytics). Additional records were sourced by examining references from 1) reference lists of included studies, 2) reference lists of relevant reviews, and 3) reference lists of industry white papers (Down Under Enterprises, 2016a; Down Under Enterprises, 2016b; Down Under Enterprises, 2017a; Down Under Enterprises, 2017b). All records identified were exported or entered manually into Microsoft Excel version 16 to permit recording and analysis of eligibility assessments. One author screened all identified records by title and abstract against eligibility criteria. For eligible abstracts, the full-text article was located and downloaded. Full-text articles were then assessed against eligibility criteria by two authors (LK and RL) with any discrepancies in judgements discussed and, where required, the opinion of a third author sought.

### 2.3 Data extraction and synthesis

One author (LK) extracted the following data from all included articles first author, year of publication, trial registration (if applicable), study design, country, study setting, participant characteristics, eligibility criteria, details of the intervention including tea tree oil source and dose(s), details of comparison group(s) including product name(s), brand(s) and manufacturer(s), outcome measures including their methods and timepoints of assessment, main findings (efficacy) and safety, funding source(s) and any potential conflicts of interest. Where one or more data items required for extraction were missing from the article text, an attempt was made to contact the corresponding author(s) for these data. Data were verified by another author (RL).

### 2.4 Risk of bias

Risk of bias within included studies was assessed using the Cochrane risk-of-bias 1.0 tool for assessing risk of bias in randomized trials (RoB 1.0) (Higgins et al., 2011). The RoB 1.0 tool comprises six domains of bias, i.e., selection bias, performance bias, detection bias, attrition bias, reporting bias, and other sources of other bias. Selection bias occurs when participants are allocated treatments using a non-random (or quasi-random) sequence, e.g., alternating allocation based on hospital admission. This may also allow personnel allocating treatments to foresee assignment and result in biased allocations. Performance bias may occur when participants know which treatment they have been allocated and systematically change their behavior as a result or when personnel are aware of participants’ allocations and systematically change their delivery of the intervention(s). Detection bias may arise if those assessing the outcome(s) measured in the study are unblinded, as this may alter how these outcome(s) are assessed. Attrition bias occurs when participants drop-out from the study creating missing data, particularly where numbers or reasons for drop-out are imbalanced between treatment arms. Reporting bias arises from the selective reporting of study outcomes, e.g., five outcomes were reportedly measured in the study, but the results of only three outcomes are reported. Two authors (LK and RL) independently assessed each of the included studies against these domains and, for each study, provided a rating (i.e., low, unclear, or high risk of bias) for each domain. Any disagreements were discussed, and judgement of a third independent author sought if required. Assessments for performance and detection bias were based on primary outcomes measured (or all outcomes if no primary outcomes were defined) and were further divided according to the nature of outcomes measured (i.e., objective, or subjective). Risk of bias assessments were tabulated and summarized thematically under each main topic (e.g., dentistry or dermatology). Where information in the full-text article text was inadequate to permit a decision on eligibility or risk of bias, or where data items required for extraction were missing, an attempt was made to contact the corresponding author(s) for this information.

## 3 Results

**FIGURE 1:**
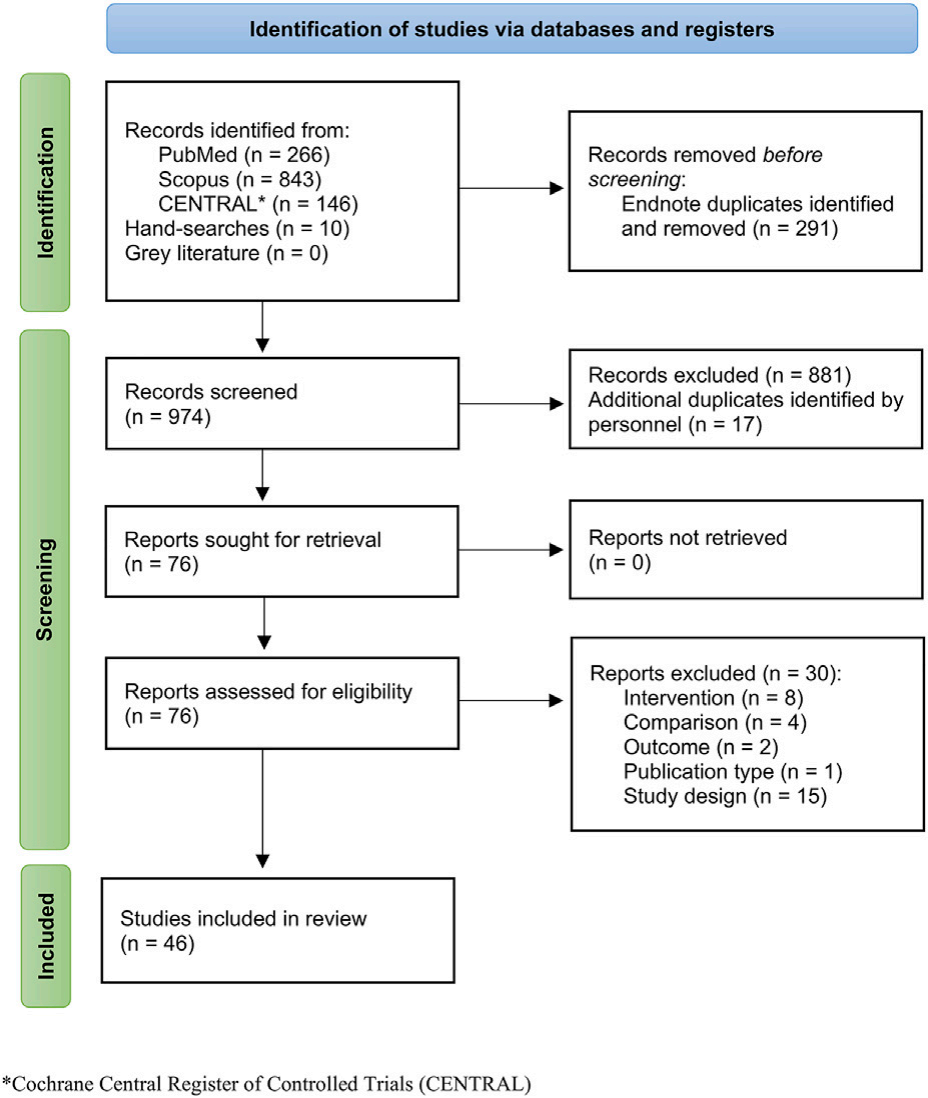
PRISMA flow diagram for study identification, screening, and inclusion.

Figure 1 presents the PRISMA flow diagram for study inclusion and exclusion. Of the total 974 records screened by title and/or abstract, 76 full-text articles were retrieved and assessed for eligibility. Thirty were excluded, primarily due to use of a study design other than a RCT, resulting in 46 full-text articles being included in this review (Figure 1).

### 3.1 Characteristics of included trials

Five fields of medicine categorized 45 of the 46 included studies, i.e., dermatology, dentistry, infectious disease, ophthalmology, and podiatry. The remaining study tested the effect of tea tree oil on anxiety and sleep disturbance in patients receiving chemotherapy (Ozkaraman et al., 2018). Eighteen studies were published in the field of dentistry addressing control of microbial plaque [*n* = 12; (Groppo et al., 2002; Saxer et al., 2003; Soukoulis and Hirsch, 2004; Prabhakar et al., 2009; Chandrdas et al., 2014; Rahman et al., 2014; Salvatori et al., 2017; Casarin et al., 2019; Bharadwaj et al., 2020; Kamath et al., 2020; Reddy et al., 2020; Ripari et al., 2020)], periodontitis [*n* = 3; (Elgendy et al., 2013; Raut and Sethi, 2016; Taalab et al., 2021)], denture stomatitis [*n* = 1; (Catalán et al., 2008)], oral halitosis [*n* = 1; (Srikumar et al., 2022)], or prevention of patient-clinician cross-contamination during dental procedures [*n* = 1; (Shetty et al., 2013)]. Nine studies were published in the field of dermatology addressing acne vulgaris [*n* = 3; (Bassett et al., 1990; Enshaieh et al., 2007; Najafi-Taher et al., 2022)], seborrheic dermatitis [*n* = 1; (Beheshti Roy et al., 2014)], inflammatory skin disease [n = 1; (Beikert et al., 2013)], wound healing [*n* = 2; (Rothenberger et al., 2016; Cho and Choi, 2017)], skin photo-aging [*n* = 1; (Hugo Infante et al., 2023)], or dandruff [*n* = 1; (Satchell et al., 2002a)]. Nine studies were published in the field of infectious disease addressing hand disinfection [*n* = 3; (Gnatta et al., 2013; Gnatta et al., 2021; Youn et al., 2021)], MRSA decolonization [*n* = 4; (Caelli et al., 2000; Dryden et al., 2004; Blackwood et al., 2013; Lee et al., 2014)] or prevention of MRSA colonization [*n* = 1; (Blackwood et al., 2013)], molluscum contagiosum [*n* = 1; (Markum and Baillie, 2012)], or oral *Candida* infection [*n* = 1; (Maghu et al., 2016)]. Six studies were published in the field of ophthalmology addressing *Demodex* infestation [*n* = 4; (Koo et al., 2012; Karakurt and Zeytun, 2018; Wong et al., 2019; Craig et al., 2022)], dry eye post cataract surgery [*n* = 1; (Mohammadpour et al., 2020)], or meibomian gland dysfunction [*n* = 1; (Zarei-Ghanavati et al., 2021)]. Three studies were published in the field of podiatry addressing onychomycosis [*n* = 1; (Buck et al., 1994)], or tinea pedis [*n* = 2; (Tong et al., 1992; Satchell et al., 2002b)]. Tables 1–6 summarize the characteristics of included studies i.e., country, setting, sample size and demographic profile, interventions, comparisons, and outcome measures, as well as results for efficacy and safety, according to the health problem studied e.g. denture stomatitis or MRSA decolonization.

**TABLE 1:** Characteristics of included studies—Dentistry.

Author; Year of publication; Trial registration	Study design; Country; Setting	Participant characteristics	Eligibility criteria	Interventions; Comparisons	Outcome measures (*methods*); Timepoints	Main findings	Safety	Funding; Conflict of interest
***Oral hygiene practices***								
Bharadwaj; 2020; trial not registered Bharadwaj et al. (2020))	RCT (parallel); India; Tertiary education institution	N = 60 enrolled (*n = 60 analysed*) Age in years, mean (SD): TTO group 19.7 (1.0) Control group 1 19.1 (0.9) Control group 2 19.4 (1.0) Female: TTO group 8/20 Control group 1 7/20 Control group 2 6/20	*Inclusion criteria*: age 18–25 years; PI score >1 and GI score >1 in 10% of affected sites. *Exclusion criteria*: current orthodontic treatment; use of a removable appliance or use of a mouthwash; allergies or systemic diseases	Intervention group: TTO mouthwash (TTO concentration not stated, *Melaleuca alternifolia*), Naturalis Essence of Nature, TSBT International, India. Other ingredients: 2.5 g Tween-80, 5 g propylene glycol, 5 g glycerine, 0.2 g benzyl alcohol and Milli-Q water. Control group 1: Chlorine dioxide mouthwash, Freshchlor®, Group Pharmaceuticals Ltd, India. Control group 2: Chlorhexidine mouthwash, Guard-OR®, Group Pharmaceuticals Ltd, India. All subjects swished with 10 mL of allocated mouthwash for 30 seconds, twice daily, for 3 weeks. Subjects were instructed to avoid eating or drinking for 30 minutes after using the mouthwash, and to continue their usual oral hygiene practices.	**PI** (*measured using mouth mirrors and dental explorers, disclosing solution, according to the modified Silness-Löe index*) **GI** (*measured using mouth mirrors and periodontal probes according to the Löe-Silness index*) Patient-reported overall rating of mouthwash. Timepoints: Baseline and after 3 weeks.	**PI** sig. greater mean reduction in TTO group (-0.261) compared with chlorine dioxide group (-0.145) after 3 weeks (*p =0.011*). Mean reduction lower in TTO group (-0.261) compared with chlorhexidine group (-0.388) after 3 weeks (*p = 0.063*) **GI** sig. greater mean reduction in chlorhexidine group (-0.438) compared with TTO group (-0.305) after 3 weeks (*p =0.024*). No difference between TTO group (-0.305) and chlorine dioxide group (-0.227) after 3 weeks (*p = 0.326*) Patient-reported rating of mouthwash as ‘good’ was 85% for TTO and chlorine dioxide mouthwashes respectively, and 80% for Chlorhexidine mouthwash.	Not assessed.	Authors state no funding and no conflicts of interest. Acknowledgement of support from Group Pharmaceuticals Ltd employee (company provided two of the mouth rinses).
Casarin; 2019; NCT02695901; Casarin et al. (2019)	RCT (cross-over); Brazil; Tertiary education institution	N = 60 enrolled (*n = 60 analysed*) Age in years, mean (SD): 24.7 (5.7) Female: 63.7%	*Inclusion criteria*: age ≥ 18 years; healthy (i.e. not undergoing medical treatment); ≥ 6 teeth per quadrant. *Exclusion criteria*: allergy to TTO or chlorhexidine; recent use of chlorhexidine or other antiseptic; use of fixed and/or removable prostheses; use of orthodontic appliance; dental caries; maladapted restorations; lesions involving the oral mucosa; active infectious foci (endodontic or periodontal abscesses); history of periodontitis (i.e., clinical attachment loss > 3mm in two or more nonadjacent teeth); marginal gingival bleeding > 15%; any systemic condition that could affect gingival health (e.g. pregnant or lactating, tobacco use); having undergone local or systemic antimicrobial treatment within 90 days prior to study.	Intervention group: TTO mouthwash (0.3% TTO nanoparticles, *Melaleuca alternifolia*), Inventiva, Brazil. Other ingredients: acetyl palmitate and polysorbate 80. Control group: Chlorhexidine mouthwash, Periogard®, Colgate-Palmolive, Brazil. Subjects received professional prophylaxis and stopped all oral hygiene practices for 72 hours. On day 3, gingival crevicular fluid was collected and subjects received professional prophylaxis to two randomly selected contralateral quadrants (either Q1-Q3 or Q2-Q4) providing one surface area that was biofilm free and one that was biofilm covered. Subjects were then instructed to swish with 15mL of their allocated mouthwash for 60 seconds twice daily for 4 days (with no additional oral hygiene practices). Subjects then resumed usual oral hygiene practices for a 21-day washout period, before repeating the experiment using the other mouthwash.	**PI** (*clinician assessed using Quigley & Hein PI score modified by Turesky et al. after applying two-tone disclosing solution Young Dental, Earth City, US)* Subjects’ perceptions (*assessed by VAS from 0 = negative extreme to 10 = positive extreme for taste of the product, duration of taste, change in taste, application time, comfort of use and perception of biofilm control*) Timepoints: day 7 only Gingival crevicular fluid volume (*collected using Periopaper® strip by Oralflow, US, and assessed using Periotron 8000® by Oralflow, US*) Timepoints: day 3 (baseline) and day 7	Mean **PI** sig. lower in chlorhexidine group compared with TTO group on day 7, on both biofilm free surfaces (2.65 ± 0.34 vs. 3.34 ± 0.33, *p < 0.05*) and biofilm covered surfaces (2.84 ± 0.37 vs. 3.37 ± 0.33, *p < 0.05*). Gingival crevicular fluid volume non-sig. difference between TTO and chlorhexidine groups on day 7. Subjects perceived sig. better taste of product and biofilm control, but also greater change in taste, with chlorhexidine mouthwash compared with TTO mouthwash (all *p < 0.001*).	No serious adverse events or side effects reported by subjects.	Authors state no funding and no conflicts of interest.
Chandrdas; 2014; trial not registered Chandrdas et al. (2014)	RCT (parallel); India; Tertiary education institution	N = 210 Age: not reported Female 105/210	*Inclusion criteria*: age 18–25 years; residing on campus; DMFT score ≤ 3. *Exclusion criteria*: current orthodontic treatment; extensive intra-oral prosthesis; antibiotic medication or antiseptic mouthwash use within three months prior to study (or at enrolment).	Intervention group: TTO toothbrush sanitising solution (0.2% TTO, *Melaleuca alternifolia*), Mother Herbs Private Ltd., India. Other ingredients: distilled water and 0.5% Tween 80. Control group 1: 3% garlic toothbrush sanitising solution made from 12 mL of fresh garlic obtained from local market mixed with distilled water. Control group 2: Chlorhexidine toothbrush sanitising solution, Hexidine®, ICPA Health Products Ltd, India. Control group 3: 0.05% cetylpyridinium chloride toothbrush sanitising solution made from 15mg cetylpyridinium chloride powder (CDH Laboratory, India) mixed with distilled water. Control group 4: UV toothbrush sanitizing device, VIOLight Toothbrush Sanitizer®, Violight Inc., US. Control group 5: Distilled water toothbrush sanitising solution. All subjects brushed twice daily with new toothbrushes (Oral B Shiny Clean, Procter & Gamble, India) for two weeks. Toothbrushes were then immersed in the allocated toothbrush sanitising solution for 12 hours and then subject to microbial analysis.	***Streptococcus mutans* count** on used toothbrushes (*CFU/mL counted after 48-hr incubation on Mitis salivarius agar at 37°C*). Timepoints: Baseline and after 2 weeks.	***Streptococcus mutans* count** on toothbrushes sig. decreased in all groups, including distilled water group, compared with baseline (*p < 0.001*). Largest decrease in *Streptococcus mutans* count was in the garlic group from 102.87 ± 12.59 to 0.0 ± 0.0, *p < 0.001*. In TTO group, *Streptococcus mutans* decreased from 103.47 ± 14.42 to 59.20 ± 14.99, *p < 0.001*. *Note: S. mutans counts differed significantly across groups at baseline (ANOVA p < 0.001).*	Not assessed, but solutions also not tested on human participants.	Authors state no funding and no conflicts of interest.
Groppo; 2002; trial not registered Groppo et al. (2002)	RCT (parallel); Brazil; Not described	N = 30 enrolled (*n analysed not reported*) Age: not reported Female 16/30	*Inclusion criteria*: healthy; age 18–35 years; all teeth except third molars. *Exclusion criteria*: allergies; microbial agent used within two weeks prior to study.	Intervention group: TTO mouthwash (0.2% TTO, *Melaleuca alternifolia*), imported from Australia by Galena Pharmacy Ltd. Other ingredients: vehicle solution and 0.5% Tween 80 Control group 1: 2.5% garlic mouthwash (*Allium sativum* fresh bulb obtained from local supermarket) mixed with vehicle solution Control group 2: 0.12% chlorhexidine mouthwash (chlorhexidine gluconate – source unknown) mixed with vehicle solution **All subjects:** **Week 1**: no oral hygiene practiced. **Week 2:** 1 min mouthwashes using 10 mL vehicle solution (i.e., distilled water with 5% spearmint essence, and 2% sorbitol) 30 minutes after the last tooth brushing of the day. **Week 3:** subjects swished with allocated mouthwash daily 30 minutes after last tooth brushing of the day.	**Salivary total microorganism count** (*total CFU/mL counted after 48-hr incubation on blood agar Difco Co. at 37°C, followed by an aerobic incubator at 37°C for 24hours*) **Salivary *Streptococcus mutans* count** (*CFU/mL counted after 48-hr incubation on Mitis salivarius agar Difco Co. at 37°C*) Adverse events (*self-reported on VAS for following events solution taste, breath alteration, burning sensation, tooth colour alteration, and systemic adverse effects: 0 = None, 0 < 2.5 = low, 2.5 < 5.5 = moderate, 5.5 < 8.5 = serious, 8.5 < 10 = severe*) Timepoints: Baseline (week 1) and weeks 2 (control phase), 3 (experimental phase), 4 and 5 (regrowth phase).	*Note: study only reports within group changes.* **Total salivary microorganism count** sig. decreased in TTO group during mouthwash use (i.e. Week 3) and in both TTO and garlic groups post-intervention (i.e. Weeks 4 and 5), compared with Week 1 (no oral hygiene) and Week 2 (standard oral hygiene), all *p < 0.05*. No sig. changes observed in chlorhexidine group. ***S. mutans* count** sig. decreased in all groups during mouthwash use (i.e. Week 3) and remained sig. lower in TTO and garlic groups post-intervention (i.e. Weeks 4 and 5), compared with Week 1 (no oral hygiene) and Week 2 (standard oral hygiene), all *p < 0.05*	Burning sensation sig. more intense in garlic group than TTO group (*p = 0.049*). Breath sig. worse in garlic group compared with TTO group (*p = 0.007*). Taste sig. worse in garlic group compared with TTO group (*p = 0.002*). All adverse effects between TTO group and control group 2 were not sig.	Funding: CNPq (Brazilian National Council for Scientific and Technological Development) Conflicts of interest: no data provided.
Kamath; 2020; trial not registered Kamath et al. (2020)	RCT (parallel); India; Primary education institution	N = 152 enrolled (*n analysed not reported*) Age in years, mean (SD): TTO group 12.2 (2.0) Control group 1 11.9 (1.8) Control group 2 12.2 (1.9) Control group 3 11.3 (2.2) Female: TTO group 19/38 Control group 1 13/38 Control group 2 20/38 Control group 3 11/38	*Inclusion criteria:* school children aged 8–14 years; PI score >1 and GI score >1; similar oral hygiene practices. *Exclusion criteria:* severe caries with pulp involvement; chronic systemic illness; antibiotic or anti-inflammatory medication use one month prior to, or during, study.	Intervention group: TTO mouthwash (0.5% TTO, *Melaleuca alternifolia*), Falcon essential oils, India. Other ingredients: glycerin 5g, propylene glycol 5g, Tween-80 2.5 g, benzyl alcohol 0.2g, and Milli-Q water. Control group 1: aloe vera mouthwash (aloe vera 7 g, Falcon essential oils, India). Other ingredients: peppermint oil 0.025 g, Tween-80 0.5 g, benzyl alcohol 0.2 g, and Milli-Q water). Control group 2: chlorhexidine mouthwash (0.2% chlorhexidine gluconate), brand and manufacturer not stated. Control group 3: placebo mouthwash (distilled water). All subjects swished with 10 mL of allocated mouthwash for 30 seconds, twice daily, for 4 weeks. Subjects were instructed to avoid eating or drinking for 30 minutes after using the mouth rinse, and to continue their usual oral hygiene practices.	PI (*according to the Silness-Löe Index*) GI (*according to the Löe-Silness GI*) Salivary *Streptococcus mutans* CFU/ml (*assessed by microbiological analysis using Mitis salivarius agar* *culture media, Hi-Media company.)* Timepoints: Baseline and at week 4 (post-intervention) and week 6 (two weeks after end of intervention).	PI sig. reduction in TTO group compared with placebo group (*p < 0.001*). *Note: sig. reduction also found for aloe vera and chlorhexidine groups compared with placebo (p < 0.001)* GI sig. reduction in TTO group compared with placebo group (*p < 0.001*). *Note: sig. reduction also found for aloe vera and chlorhexidine groups compared with placebo (p < 0.001).* Salivary *Streptococcus mutans* CFU/ml sig. reduction in TTO group compared with placebo group (*p* < 0.001). *Note: sig. reduction also found for aloe vera and chlorhexidine groups compared with placebo (p < 0.001).* No sig. difference in any of the measured outcomes between TTO, aloe vera and chlorhexidine groups.	Not assessed.	Funding: no data provided. Authors state no conflicts of interest.
Prabhakar; 2009; trial not registered Prabhakar et al. (2009)	RCT (parallel); India; Primary education institution	N = 36 (*n analysed not reported*) Sample not described.	*Inclusion criteria*: aged 9-11 years; DMFT > 3 *Exclusion criteria*: antibiotic therapy one month prior to study; systemic disease; history of fluoride or topical fluoride use; no allergies to herbal products tested.	Intervention group: 0.2% TTO mouthwash (0.2% TTO, *Melaleuca alternifolia*) Thursday Plantations Ltd. Australia. Other ingredients: 0.5% Tween 80 and distilled water. Control group 1: curry leaf mouthwash (2.5% fresh curry leaves obtained from local market mixed with distilled water) Control group 2: garlic mouthwash (2.5% white garlic obtained from local market mixed with distilled water) Control group 3: placebo mouthwash (not defined) All subjects swished with 10 mL for one minute, 30 minutes after brushing teeth, twice daily, for seven days.	**Salivary Streptococcus mutans and Lactobacilli** CFU/ml (*assessed by microbiological analysis using Mitis Salivarius Bacitracin agar and Rogassa L agar*) Adverse events (*assessed on visual analogue 10 cm ruler scale where 0 cm = None; 0cm < 2.5 cm = Low; 2.5 cm < 5.5 cm = Moderate; 5.5cm < 8.5 cm = Serious; and 8.5 cm < 10 cm = Severe*) Timepoints: Baseline (Day 0, 30 minutes after brushing teeth, i.e., pre-mouthwash), Day 0 (30 minutes after first saliva sample and after swishing with allocated mouthwash, i.e. post-mouthwash), Day 3, Day 7, and Day 14.	**Salivary S. mutans and Lactobacilli** sig. decreased in TTO, curry leaf and garlic groups from baseline to Day 7 (*p = 0.008*). **Salivary S. mutans and Lactobacilli** remained sig. lower than baseline in TTO and garlic groups at Day 14 (*p = 0.008*). *Note: only within group analyses performed. Results of placebo group not reported.*	Adverse effects - *Unpleasant taste*: curry leaves 44.4%, garlic 88.9%, TTO 66.6% - *Burning sensation*: curry leaves 55.6%, garlic 88.9%, TTO 77.8% - *Bad breath*: curry leaves 44.4%, garlic 100%, TTO 22.2% - *Nausea*: curry leaves 0%, garlic 100%, TTO 44.4%	No data provided.
Rahman; 2014; trial not registered Rahman et al. (2014)	RCT (cross-over); United Arab Emirates; University campus	N = 20 (*n = 20 analysed*) Age in years, mean (SD): 22.6 (1.8) Female: 16/20	*Inclusion criteria*: minimum 20 teeth. *Exclusion criteria*: pregnant or lactating; systemic disease; orthodontic appliances; periodontitis; known allergy to any of the components of mouthwashes; antibiotic use within 3 months prior to study.	Intervention group: TTO mouthwash (1.5% TTO, *Melaleuca alternifolia*), Tebodont®, DrWild and Co AG, Switzerland. Other ingredients: Aqua, Xylitol, Sorbitol, Glycerin, Propylene Glycol, PEG-40-Hydrogenated Castor Oil, Aroma, Sodium Saccharin, Limonene. Control group 1: cetylpyridinium chloride mouthwash, Aquafresh®, GlaxoSmithKline Consumer Healthcare, UK. Control group 2: chlorhexidine mouthwash, Oro-Clense®, Germiphene Corporation, Canada. Control group 3: placebo mouthwash (coloured water). Subjects swished with allocated mouthwash twice daily according to manufacturer’s instructions, for five-day test period. During each five-day test period, subjects were instructed to suspend all oral hygiene practices. Crossover was performed using a two-week washout period where subjects resumed usual oral hygiene practices followed by professional tooth scaling and polishing prior to starting a new five-day test period.	**PI** (*assessed according to Turesky's modified Quigley-Hein*) **GBI** (*assessed by gentle probing of the gingival crevice with periodontal probe and recording any bleeding within 10 seconds, reported as percentage of gingival margins assessed*) Timepoints: Pre-treatment and post-treatment (after five-day test period).	**PI** non-sig. reduction in Tebodont® group compared with placebo. Reduction sig. greater with Oro-Clense® compared with Tebodont® (*p =0.019*) and with placebo (*p =0.001*). **GBI** no sig. difference between post-test scores of Tebodont®, Aquafresh®, Oro-Clense®, or placebo groups.	Side effects: *Bitter taste*: TTO group =1/20, chlorhexidine group = 4/20, cetylpyridinium chloride group = 1/20 *Burning sensation*: TTO group = 1/20, chlorhexidine group = 2/20, cetylpyridinium chloride group = 1/20 *Dry mouth*: chlorhexidine group = 1/20 *Tooth staining*: chlorhexidine group = 2/20	Funding: University of Sharjah, grant no. 101006). Conflicts of interest: no data provided.
Reddy; 2020; trial not registered Reddy et al. (2020)	RCT (parallel); India; Secondary education institution	N = 90 enrolled (*n analysed not reported*) Age in years, range: 12–15 Female: 47/90	*Inclusion criteria*: age 12–16 years; ≥20 teeth; moderate to severe plaque induced gingivitis. *Exclusion criteria*: orthodontic appliance; known allergy to any component of the test mouthwashes; antibiotic use within 3 months prior to study; systemic disease.	Intervention group: TTO mouthwash (0.2% TTO, *Melaleuca alternifolia*), brand and manufacturer not stated. Other ingredients: 2g Tween 80 and 2L distilled water. Control group 1: chlorhexidine mouthwash (procured from pharmacy, 0.12% chlorhexidine, not further defined). Control group 2: placebo mouthwash (not defined). Subjects swished with 10 mL mouthwash once daily at 9 am, for 15 days, and were instructed to avoid eating or drinking for following 30 minutes. All subjects followed their usual oral hygiene practices. At baseline all subjects received professional tooth scaling and polishing.	**PI** (*according to the Silness-Löe index*) **GI** (*according to the Löe-Silness index*) Adverse events (*self-reported change in taste perception or breath, burning sensation, and staining*). Timepoints: Baseline, Day 7 and Day 15 (post-intervention)	*Note: mean difference between groups post-intervention calculated in Review Manager 5.4.* **PI:** TTO group vs. chlorhexidine group Baseline: 1.92 ± 0.38 vs. 1.99 ± 0.36 (mean diff -0.07 [-0.26, 0.12]) Day 15: 1.63 ± 0.33 vs. 1.74 ± 0.31 (mean diff -0.11 [-0.27, 0.05]) TTO group vs. placebo Baseline: 1.92±0.38 vs. 2.11±0.38 (mean diff -0.19 [-0.38, 0.00]) **Day 15: 1.63 ± 0.33 vs. 2.02 ± 0.38 (mean diff. -0.39 [-0.57, -0.21])** **GI:** TTO group vs. chlorhexidine group Baseline: 1.04 ± 0.31 vs. 1.06 ± 0.33 (mean diff. -0.02 [-0.18, 0.14]) * **Day 15: 0.84 ± 0.29 vs. 1.14 ± 0.37 (mean diff. -0.30 [-0.47, -0.13])** *Conflicts with results reported in text TTO group vs. placebo **Baseline: 1.04 ± 0.31 vs. 1.24 ± 0.35 (mean diff -0.20 [-0.37, -0.03]** **Day 15: 0.84 ± 0.29 vs. 1.08 ± 0.37 (mean diff. -0.24 [-0.41, -0.07])**	TTO group 1/30 reported changes in taste and breath as well as burning sensation.	No data provided.
Ripari; 2020; trial not registered Ripari et al. (2020)	RCT (parallel); Italy; Hospital clinic	N = 42 enrolled (*n = 42 analysed*) Age in years, range: 18–60 Female: 30/42	*Inclusion criteria*: age >18 years; ≥20 teeth (excluding third molars); GI ≥1 and <3; PI ≥1 and <3; presence of pseudopockets; bleeding on probing. *Exclusion criteria*: known allergy to any component of the test mouthwashes; periodontitis; tooth mobility; periodontal treatment within 6 months prior to study; systemic disease; clinical attachment loss >4 mm; periodontal pockets; mental or physical retardation that could have influenced domestic oral hygiene.	Intervention group: TTO mouthwash (9 drops i.e., 0.65 mL of 100% TTO, *Melaleuca alternifolia*) brand and manufacturer not stated. Other ingredients: water. Subjects swished with mouthwash, 1 – 3 times daily depending on preferred frequency, as long as 9 drops TTO were used each day diluted in water, for 14 days. Control group: chlorhexidine mouthwash (0.12% chlorhexidine, not further defined); subjects swished with 5 mL of mouthwash, twice daily, for 14 days All subjects provided with same oral hygiene instructions: use of medium bristle brush, same toothpaste and Bass modified brushing technique. Subjects swished with mouthwash for 60 seconds and instructed to avoid eating or drinking for following 30 minutes.	PI (*reported as percentage of plaque present according to O’Leary index – also states Silness- Löe index was used but reported results do not reflect this index*) GI (*according to the Löe-Silness index*) GBI (*reported as percentage according to Ainamo & Bay*) Probing depth (*measured in mm using William’s probe*) Dental dyschromia (*recorded as present or absent and expressed as percent average for all surfaces assessed*). Timepoints: Baseline and after Day 14	*Note: results analysed in SPSS using ANCOVA using raw data provided in manuscript.* PI non-sig. difference between TTO group (5.50 ± 4.45) and control group (3.28 ± 3.31), after 14 days (*p =0.166*). GI sig. lower in TTO group (0.32 ± 0.48), compared with control group (0.95 ± 0.69), after 14 days (*p < 0.001*). GBI non-sig. difference between TTO group (4.22 ± 6.09) and control group (6.29 ± 5.95), after 14 days (*p = 0.988*). Probing depth sig. lower in TTO group (0.68 ± 0.78), compared with control group (1.35 ± 1.04), after 14 days (*p = 0.016*). Dental dyschromia TTO group 0/22 and control group 4/20	TTO group: Nausea 4/22 Control group: Taste changes when eating salted and spicy foods 4/20; Burning sensation 12/20	Funding: no data provided. Authors state no conflicts of interest.
Salvatori; 2017; trial not registered Salvatori et al. (2017)	RCT (unclear – methods state ‘cross-over’, but study procedure described as having been conducted in parallel); Italy; Hospital clinic	N = 16 enrolled (*n = 16 analysed*) Age in years, range: 21-37 Female: 9/16	*Inclusion criteria*: age 18–70 years; ≥20 teeth (excluding third molars); Gingivitis; Periodontal Screening and Recording score 1–2; PPD ≤3mm. *Exclusion criteria*: known allergy to any component of the test mouthwashes; periodontal disease PPD >3 mm; periodontitis; orthodontic appliance; cortisone use; anti-inflammatory medication use within 3 months prior to study; periodontal treatment or antibiotic use within 6 months prior to study; systemic disease that may affect the intervention; pregnancy; oral contraceptive use; mental or physical limitations restricting home oral hygiene practices.	Intervention group: TTO mouthwash (1.5% TTO, *Melaleuca alternifolia*), Tebodont®, DrWild and Co AG, Switzerland. Other ingredients: Aqua, Xylitol, Sorbitol, Glycerin, Propylene Glycol, PEG-40-Hydrogenated Castor Oil, Aroma, Sodium Saccharin, Limonene. Control group 1: chlorhexidine mouthwash (0.12% chlorhexidine, not further defined). Control group 2: Essential oil mouthwash (thymol, menthol, eucalyptol, limonene, sodium fluoride, zinc and xylitol). Control group 3: placebo mouthwash (100 mL red food dye diluted in 2L of water). Subjects swished with allocated mouthwash for 2 weeks. At baseline, all subjects received professional tooth scaling and polishing. The same oral hygiene instruction was then given to all subjects to be followed while using their allocated mouthwash.	**Full Mouth Plaque Score** (*not further defined*) **Full Mouth Bleeding Score** (*not further defined*) **GI** (*not further defined*) Timepoints: Baseline and after 2 weeks (i.e., post-intervention) Dental discoloration (*recorded as present or absent and expressed as percent average for all surfaces assessed*). Lingual Patina Index (*clinician-assessed from 0–4 based on degree of patina presence*) Timepoints: After 2-weeks (i.e., post-intervention)	*Note: results analysed in SPSS using ANCOVA using raw data provided in manuscript.* **Full Mouth Plaque Score** decreased in all groups. No sig. difference between groups, after 2 weeks (*p = 0.694*). **Full Mouth Bleeding Score** decreased in all groups. No sig. difference between groups, after 2 weeks (*p = 0.070*). **GI** decreased in all groups. No sig. difference between groups, after 2 weeks (*p = 0.189*). Dental discoloration reported to be greater in chlorhexidine group. Lingual Patina Index no difference between groups at follow-up.	Taste changes (*self-assessed using VAS*) reported by 2/4 in control group 1 (chlorhexidine).	No data provided.
Saxer; 2003; trial not registered Saxer et al. (2003)	RCT (parallel); Switzerland; Outpatient clinic	N = 30 enrolled *(n = 26 analysed)* TTO group = 13 Placebo = 13 Age: not reported Female: not reported	*Inclusion criteria*: age 18–65 years; ≥20 teeth (max. 4 crowned teeth i.e., one per quadrant); generally healthy; brushing teeth ≥2 times per day; mean sulcus bleeding index of >1.5 (otherwise dentition was in good condition). *Exclusion criteria*: smokers; medical risk factors; PPD >5mm.	Intervention group: TTO mouthwash (1.5% TTO, *Melaleuca alternifolia*), Tebodont®, DrWild and Co AG, Switzerland. Other ingredients: Aqua, Xylitol, Sorbitol, Glycerin, Propylene Glycol, PEG-40-Hydrogenated Castor Oil, Aroma, Sodium Saccharin, Limonene. Control group: placebo mouthwash (Sorbitol, Glycerin, Aqua, PEG-40-Hydrogenated Castor Oil, Sodium Saccharin, Aroma), provided by DrWild and Co AG, Switzerland. Subjects swished with 10mL mouthwash for 60–90 seconds, three times daily (within 30 minutes of cleaning their teeth), for 12 weeks. After randomisation, all subjects were provided the same toothbrush (Emoform sensitive) and toothpaste (Colgate Gel) products to use for the entire duration of the study. During the intervention, subjects were prohibited from flossing or using other mouthwash products.	**Sulcus Bleeding Index** (*assessed according to Mühlemann & Son, 1971*) **PI** (*assessed according to Turesky Index*) Patient questionnaire (subjectively assessed effects of treatment e.g., clean mouthfeel, prevention of plaque formation, inflammation, taste changes, overall taste, tongue coatings and changes in oral mucosa) Timepoints: Baseline and after weeks 3 and 12.	**Sulcus Bleeding Index** sig. decrease within both TTO and control groups from baseline to 12-weeks (*p < 0.01*). Difference between groups after 12 weeks non-sig. **PI** not sig. between groups after 12 weeks (*p = 0.06*). Within TTO group, non-sig. reduction from baseline to after 12 weeks. Within placebo group, non-sig. increase from baseline to after 12 weeks. Mean overall taste (rated 1–5; most positive = 5) after 12 weeks: TTO group 1.38 Placebo group 2.08	Minor changes in the oral mucosa reported by 24% in TTO group and 8% in placebo group. *Note: researchers could not attribute changes in oral mucosa to use of the mouthwashes.*	No data provided. Test products provided by Dr. Wild & Co. AG, Switzerland.
Soukoulis; 2004; trial not registered Soukoulis and Hirsch, (2004)	RCT (parallel); Australia; Not described	N = 58 enrolled *(n = 49 analysed)* Age in years, mean (SD): 45.6 (9.5), range 26–63 Female: 24/49	*Inclusion criteria*: age 18–60 years; moderate to severe gingivitis (GI 2–3 in ≥1 tooth per quadrant); ≥20 teeth. *Exclusion criteria*: smokers; diabetes, hepatic, or kidney disease; rheumatoid arthritis; pregnant or lactating; periodontal therapy within 6 months prior to study; known allergy to TTO; use of steroids, NSAIDs, Dilantin (Phenytoin), or antibiotics for ≥7 days within 6 months prior to study; subjects requiring antibiotics for dental treatment.	Intervention group: TTO gel (vehicle gel with 2.5% TTO, botanical species not stated) Control group 1: chlorhexidine gel (Perioguard®, Colgate, Australia) Control group 2: placebo gel (vehicle gel without TTO) Subjects applied gel to supplied toothbrushes and used as a toothpaste ensuring contact with gingival tissues for ≥2 minutes, twice daily. After using gel, subjects were instructed to avoid eating or drinking for following 30 minutes. Subjects prohibited from using other toothpastes, mouthwashes, or other cleaning aids during the study.	GI (*according to the Löe-Silness index, i.e., 1 = Normal gingiva, 1 = Mild inflammation, 2 = Moderate inflammation, 3 = Severe inflammation*) Papillary Bleeding Index (*not further described*) Plaque Surface Score (*modified version of Turesky PI using Disclogel, Colgate, Australia applied to the teeth as disclosing solution*) Timepoints: Baseline, and at weeks 4 and 8.	GI no sig. difference between TTO and Perioguard® or placebo. Papillary Bleeding Index sig. decrease in TTO group (only for posterior teeth and buccal surfaces) compared with Perioguard® and placebo (*data not provided in paper*). Plaque surface score no sig. difference between TTO group and Perioguard® or placebo.	No reports of adverse reactions to any of the gels.	Funding: no data provided. Conflicts of interest: no data provided.
***Periodontitis***								
Elgendy; 2013; trial not registered Elgendy et al. (2013)	RCT (parallel); Egypt; Outpatient clinic	N = 40 enrolled (*n = 40 analysed*) Age: not reported Female: 19/40	*Inclusion criteria*: age 30–60 years; diagnosis of moderate to severe chronic or recurrent periodontitis (untreated); single rooted teeth with PPD 5–8mm, without recession, that bleed on probing. *Exclusion criteria*: periodontal surgery within prior 24 months; systemic disease affecting periodontium; pregnant or post-menopausal; antibiotic or anti-inflammatory medication or vitamin use within 3 months prior to study; smokers >10 cigarettes/day; history of alcohol abuse; received SRP or subgingival instrumentation within two months prior to baseline; any teeth with a periodontal pocket extending to apex.	Intervention group: SRP + TTO gel (5% TTO, *Melaleuca alternifolia*) Sigma Aldrich®, Germany. Other ingredients: methyl cellulose powder and boiled water; areas where gel was applied were covered with periodontal pack (Coe-Pack) for seven days. *Note: TTO gel dose not stated* Control group: SRP only Both groups provided oral hygiene instructions and full mouth SRP.	**Pentraxin‑3 level** in gingival crevicular fluid (*samples collected using paper strips from deepest periodontal disease pocket ≥5mm and analysed by immunoassay (Quantikine®, R and D Systems Inc., US)*). **PI** (*according to the Silness-Löe index*) **GI** (*according to the Löe-Silness index*) **PPD** (*according to the periodontal disease index*) **Clinical attachment level** (*according to the periodontal disease index*) Timepoints: Baseline and at 1, 3 and 6 months	**Pentraxin‑3 level** sig. lower in TTO group compared with control group at 1 month (*p < 0.01*), 3 months (*p < 0.001*) and 6 months (*p < 0.001*). **PI** no sig. difference between TTO group and control group at 1, 3 and 6 months. **GI** sig. lower in TTO group compared with control group at 1 month (*p < 0.01*), 3 months (*p < 0.01*) and 6 months (*p < 0.001*). **PPD** sig. lower in TTO group compared with control group at 1 month (*p < 0.05*), 3 months (*p < 0.01*) and 6 months (*p < 0.01*). **Clinical attachment level** sig. lower in TTO group compared with control group at 1 month (*p < 0.01*), 3 months (*p < 0.05*) and 6 months (*p < 0.01*).	Adverse effects reported as assessed, but not reported.	Authors state no funding and no conflicts of interest.
Raut; 2016; trial not registered Raut and Sethi, (2016)	RCT (parallel ‘split-mouth’ trial); India; Outpatient clinic	N = 15 enrolled, N = 45 total affected sites Age in years, mean (SD): 37.4 (9.8), range 20–60 Female: 9/15	*Inclusion criteria*: clinical and radiographical diagnosis of moderate to severe chronic periodontitis (untreated); affected sites have PPD >5mm and clinical attachment loss >4 mm. *Exclusion criteria*: systemic disease affecting periodontium; pregnant or lactating; antibiotic or anti-inflammatory medication or vitamin use within prior three months; smokers >10 cigarettes/day; received SRP or subgingival instrumentation within two months prior to baseline examination.	Intervention group: SRP + TTO gel (5% TTO, *Melaleuca alternifolia*), Allin Exporters, India. Other ingredients: methyl cellulose powder and boiled water. Control group 1: SRP + Placebo gel (methyl cellulose powder and boiled water) Control group 2: SRP + CoQ10 gel (Perio Q®, PerioQ Inc., Manchester, US) *Note: Doses not stated* For all groups, SRP was performed first followed by application of selected gel into periodontal pocket. Periodontal pack was then applied to retain gel within periodontal pocket (removed after seven days).	**PI** (*according to the Silness-Löe Index*) **Gingival bleeding index** (*according to the sulcus bleeding index*) **PPD** (*according to the periodontal disease index*) **Clinical attachment level** (*according to the periodontal disease index*) Timepoints: Baseline and after 1 month	*Note: study only reports within group changes* **PI** sig. decrease in all groups from baseline to 1 month (*p < 0.05*). **Gingival bleeding index** sig. decrease in all groups from baseline to 1 month (*p < 0.05*). **PPD** sig. decrease in TTO and Perio Q® groups from baseline to 1 month (*p < 0.05*). Decrease in placebo group non-sig. **Clinical attachment level** sig. decrease in TTO and Perio Q® groups from baseline to 1 month (*p < 0.05*). Decrease in placebo group non-sig.	Not assessed.	Authors state no funding and no conflicts of interest.
Taalab; 2021; NCT04769271 Taalab et al. (2021)	RCT (parallel); Egypt; Outpatient clinic	N = 30 enrolled (*n = 30 analysed*) Age in years, mean (SD): TTO group 30.5 ± 5.6 Control group 28.9 ± 6.3 Female: TTO group 10/15 Control group 10/15	*Inclusion criteria*: age 25–50 years; diagnosis of stage 2 (grade B) periodontitis according to 2017 World Workshop on the Classification of Periodontal and Peri-Implant Diseases and Conditions; CAL 3–4 mm; BOP in proximal tooth surface; able to maintain an O’Leary plaque index ≤ 10%; radiographic horizontal bone loss related to the coronal third of the root (15%–33%). *Exclusion criteria*: teeth loss due to periodontitis; CAL of 3–4 mm of non-periodontal cause; systemic disease; smokers; pregnant; use of contraindicated medications (not defined); chemotherapy or radiotherapy within one year prior to study.	Intervention group: TTO gel (5% TTO, *Melaleuca alternifolia*), Sigma Aldrich®, Germany, + SRP; approximately 0.5 mL was injected into the periodontal pocket and subject was advised not to brush their teeth for the following 24 hours. Control group: SRP only All subjects received full mouth SRP using hand instruments and ultrasonic scalers, in addition to oral hygiene instructions.	PPD CAL (*measured in millimetres*) GI (*according to the Löe- Silness index*) BOP (*reported as percentage according to Ainamo & Bay*) Timepoints: Baseline and after 3 and 6 months Matrix metalloproteinase-8 in the gingival crevicular fluid (*measured in n/ml using Sandwich-enzyme-linked immunosorbent assay, Biovision Company, Ltd, China*). Timepoints: Baseline and after 1, 3 and 6 months	PPD no sig. difference between TTO group and control group after 3 (*p = 0.137*) or 6 (*p = 0.050*) months. CAL no sig. difference between TTO group and control group after 3 months (*p = 0.174*). After 6 months, sig. lower in TTO group compared with control group (*p =0.004*). GI no sig. difference between TTO group and control group after 3 months (*p = 0.250*). After 6 months, sig. lower in TTO group compared with control group (*p = 0.002*); *Note: data in Table 3 conflict with text*. BOP no sig. difference between TTO group and control group after 3 months (*p =0.250*). After 6 months, sig. lower in TTO group compared with control group (*p =0.002*). Matrix metalloproteinase-8 level no sig. difference between TTO group and control group after 1 month (*p = 0.389*) and 3 months (*p = 0.233*). After 6 months, sig. lower in TTO group compared with control group (*p = 0.005*).	No subjects reported adverse reactions to the TTO gel. Unpleasant taste reported by some subjects using TTO gel (data not reported).	Authors state no funding and no conflicts of interest.
***Denture stomatitis***								
Catalán; 2008; trial not registered Catalán et al. (2008)	RCT (parallel); Chile; Outpatient clinic	N = 27 enrolled (*n analysed not stated*) Age in years, mean (SD): 63.5 (7.4), range 50–77 Female: 26/27	*Inclusion criteria*: patients with denture stomatitis type II. *Exclusion criteria*: smoker; diabetes mellitus; hypertension; antibiotic medication use.	Intervention group: TTO tissue conditioner (TTO, *Melaleuca alternifolia*), The Australian Tea Tree Oil Research Institute Ltd., Australia. Other ingredients: Coe-Comfort, GC America Inc. For each 5mL dose of Coe-Comfort conditioner, 1 mL was removed and replaced with 1 mL of TTO). Control group 1: Nystatin tissue conditioner (Nystatin with Coe-Comfort). For each 5 mL dose of Coe-Comfort conditioner, 2 mL was removed and replaced with 2 mL of Nystatin. Control group 2: Coe-Comfort tissue conditioner. Subjects’ allocated tissue conditioner was applied to the maxillary prosthesis and replaced every four days at clinic visits for 12 days (i.e., three times).	**Salivary Candida albicans count** (*sample collected from palate mucosa using paper points DMS Dental Mirror Co. Ltd., and CFU/ml of C. albicans counted after 48 hours incubation at 37°C on Sabouraud agar plate Difco Laboratories*). Erythema of palate mucosa (*clinician assessed: 0 = none, 1=slight, 2 = moderate, 3 = severe*) Timepoints: Baseline and at 4, 8 and 12 days	**Salivary Candida albicans count** sig. decrease in TTO group compared with Coe-Comfort group at day 12 (*p < 0.0001*). Difference between TTO group and Nystatin group not sig. at day 12. Erythema of palate mucosa (i.e. palatal inflammation) sig. decreased in TTO group compared with Coe-Comfort group at day 12 (*p = 0.001*). Difference between TTO group and Nystatin group not sig. at day 12.	Not assessed.	Funding: grant DIUC 203.102.006-1-0, Universidad de Concepción, Chile. Conflicts of interest: no data provided.
***Oral halitosis***								
Srikumar; 2022; CTRI/2019/11/022041 Srikumar et al. (2022)	RCT (parallel); India; Outpatient clinic	N = 120 (*n = 118 analysed*) Age in years, mean (SD): 33.1 (11.2), range 20–53 years Female: 38/120 *(Note: total for males and females in halitosis group sums to 150, although only 120 subjects included in halitosis group, see Table 1 in publication)*	*Inclusion criteria*: Organoleptic oral malodour score > 3 on Rosenberg’s scale (0-5); Volatile Sulfur Compounds (VSC) score >157 parts per billion; probing pocket depth ≤ 4mm in any examined surface. *Exclusion criteria*: systemic condition(s) (e.g. diabetes mellitus, kidney disease, liver disease); oral bacterial infection; pregnant, lactating, or menstruating females; former or current smoker; systemic medication for oral dryness or xerostomia or systemic antibiotic therapy one month prior to intervention; periodontal disease treatment within 6 months prior to intervention.	Intervention group: TTO mouthwash (TTO concentration not stated, *Melaleuca alternifolia*), Dessert Essence, US. Other ingredients: not stated. Control group 1: chlorhexidine mouthwash, Maxxio, Alkem Laboratories, India. Control group 2: Placebo mouthwash (not further defined). All subjects swished with 10mL of their allocated mouthwash for 30 seconds, twice daily for one week. Toothpaste and toothbrush provided to all subjects to use during one-week intervention.	***S. Moorei* count in saliva and on posterior tongue surface** (*measured using real-time SYBR® Green quantitative polymerase chain reaction*). Oral halitosis (*assessed subjectively by full mouth organoleptic scoring, and objectively by presence of VSCs*). Thickness of tongue coating (*according to the Miyazaki Tongue coating index*) PI (*according to the Silness-Löe Index*) Timepoints: Baseline and after 1 week.	***S. Moorei* count in saliva and on posterior tongue surface** sig. decreased in both TTO and chlorhexidine groups (*p* < 0.001, respectively), while no change was observed in placebo group in saliva (*p* = 0.54) or on tongue (*p* = 0.61). Thus, mean reduction in *S. Moorei* in saliva and on tongue were sig. greater in both TTO and chlorhexidine groups when compared with placebo group (*p* < 0.01). Mean organoleptic score, mean VSCs and thickness of tongue coating all sig. decreased in TTO and chlorhexidine groups, compared with placebo group (all *p* < 0.05). No change in placebo group in these parameters after 1 week. Reductions in PI observed in all groups. Change in PI not sig. different between groups after 1 week.	Authors state no adverse effects were noted in any group.	Authors state no funding and no conflicts of interest.
***Patient-clinician cross-contamination***								
Shetty; 2013; trial not registered Shetty et al. (2013)	RCT (parallel); India; Outpatient clinic	N = 60 (*n analysed not reported*) Age in years, range 25–45 Female: not reported	*Inclusion criteria*: ≥ 20 teeth; oral hygiene score 1.3 – 3 (Green & Vermillion, 1960); PI 1–2 (Silness- Löe index); GI 1–2 (Löe- Silness index). *Exclusion criteria*: pacemaker, resin restoration, antibiotic use within 6 months prior to study, history of systemic disease.	Intervention group: TTO mouthwash, (TTO concentration not stated, *Melaleuca alternifolia*) Emoform®, DrWild and Co AG, Switzerland. Control group 1: chlorhexidine mouthwash, Rexidine®, Indoco Remedies Ltd, India. Control group 2: Distilled water. All subjects received ultrasonic tooth scaling. Prior to this procedure subjects swished with 10mL of their allocated mouthwash for 2 minutes.	**Viable bacterial count in dental aerosol during ultrasonic tooth scaling** (*measured in CFU/mL at three physical locations: operator’s nose level, dental assistant’s nose level, and patient’s chest level on trypticase soy agar plates – these were then incubated at 37°C for 24 hours*).	**Mean viable bacterial count** sig. lower in TTO group compared with distilled water (*p <0.001*). However, sig. higher in TTO group compared with Rexidine® (*p <0.001*).	Not assessed.	Authors state no funding and no conflicts of interest.

Abbreviations: ANCOVA, analysis of Co-Variance; ANOVA, analysis of variance; BOP, bleeding on probing; CAL, clinical attachment level; CFU, colony forming units; CoQ10, coenzyme Q10; DMFT, decayed, missing, and filled teeth; BGI, gingival bleeding index; GI, gingival index; NSAID, non-steroidal anti-inflammatory drug; PI, plaque index; PPD, probing pocket depth; Q, quadrant (e.g., Q1–Q4); RCT, randomised controlled trial; SD, standard deviation; Sig., significant (statistically); SPSS, statistical package for the social sciences; SRP, scaling and root planing; TTO, tea tree oil; UK, United Kingdom; US, United States; UV, ultraviolet; VAS, visual analogue scale; VSC, volatile sulphur compound.

**TABLE 2:** Characteristics of included studies—Dermatology.

Author; Year of publication; Trial registration	Study design; Country; Setting	Participant characteristics	Eligibility criteria	Interventions; Comparisons	Outcome measures (*methods*); Timepoints	Main findings	Safety	Funding; Conflict of interest
***Acne vulgaris***								
Bassett; 1990; trial not registered Bassett et al. (1990)	RCT (parallel); Australia; Hospital clinic	N = 124 enrolled: TTO group *n* = 61, control group *n* = 63 (*119 analysed: TTO group n = 58, control group n = 61*) Age in years: mean 19.7, range 12–35 Female: 60/124	*Inclusion criteria*: Mild-moderate acne vulgaris; age >12 years; female using acceptable method of contraception. *Exclusion criteria*: Intercurrent disease; taking systemic antibiotics, corticosteroids, retinoids, anticonvulsants, or androgens within 30 days prior to study; topical acne therapy within 2 weeks prior to study; females commencing oral contraceptive pill within 6 months prior to study; males with beards or moustaches.	Intervention group: TTO gel (5% TTO, *Melaleuca alternifolia*), Australian Plantations Pty Ltd, Australia. Delivered in water-based gel in 30 g aliquots formulated by Lederle Laboratories. Control group: Benzoyl peroxide 5% water-based lotion in 25 mL aliquots. *Note: frequency of application and duration of treatments not stated.*	**Number of inflamed lesions** (superficial and deep) **Number of non-inflamed lesions** (open and closed comedones) Oiliness, Erythema, Scaling, Pruritus, Dryness (*Graded as 0 = Nil, 1 = Mild, 2 = Moderate, 3 = Severe*) Timepoints: Baseline and at month 1, 2 and 3.	**Number of inflamed lesions** sig. lower in benzoyl peroxide group compared with TTO group at month 1 (*p < 0.05*), 2 (*p < 0.001*) and 3 (*p < 0.001*). **Number of non-inflamed lesions** no sig. difference between benzoyl peroxide and TTO groups at any timepoint. Oiliness grade sig. lower in benzoyl peroxide group compared with TTO group at month 1 (*p < 0.001*), 2 (*p < 0.02*) and 3 (*p < 0.02*). Erythema grade no sig. difference between groups at any timepoint (*Note: sig. greater erythema at baseline in TTO group compared with control group, p < 0.05*). Scaling grade and pruritus grade both sig. greater in benzoyl peroxide group compared with TTO group at month 1 (*p < 0.05*). Dryness grade sig. greater in benzoyl peroxide group compared with TTO group at month 1 (*p < 0.001*) and 2 (*p < 0.01*).	Adverse effects reported by sig. more subjects in control group 50/63 compared with TTO group 27/61, (*p < 0.001*) Primary adverse effects reported were dryness, pruritus, stinging, burning, and redness. Dryness was the most frequently reported adverse effect in both groups.	No data provided. One author affiliated with Lederle Laboratories who supplied TTO gel.
Enshaieh; 2007; trial not registered Enshaieh et al. (2007)	RCT (parallel); Iran; Outpatient clinic	N = 60 TTO gel = 30 Placebo = 30 Age in years, mean (SD): TTO group 19.3 (3.1), Placebo group 19.1 (2.6) Age in years: range 15–25 Female: TTO group 23/30 Placebo group 24/30	*Inclusion criteria*: Mild-moderate acne vulgaris (i.e., <20 comedones, <50 papules and pustules and an absence of nodules, cysts, or sinus tracts). *Exclusion criteria*: none.	Intervention group: TTO gel (5% TTO, *Melaleuca alternifolia*). Other ingredients: vehicle carbomer gel, Cinere Company, Iran. Control group: Placebo (vehicle carbomer gel only), Cinere Company, Iran. All subjects applied their allocated gel for 20 min to affected areas, twice daily for 45 days.	**Percent change in mean total acne lesions** (*papules + pustules + comedones + nodules*): **Percent change in mean acne severity index** (papules + [2*pustules] + [comedones/4]): Percent change in mean comedones, papules and pustules. Timepoints: Baseline and day 45	**Percent change in mean total acne lesions** sig. difference between TTO group (43.6% reduction in lesions) and placebo group (12.0% reduction), after 45 days (*p < 0.001*). **Percent change in mean acne severity index** sig. difference between TTO group (40.5% reduction in severity) and placebo group (7.0% reduction), after 45 days (*p < 0.001*). (*Note: data for placebo group conflicts between text and* *Figure 1*). Percent change in mean comedones sig. greater in TTO group (40.2% reduction) compared with placebo group (12.1% reduction), after 45 days (*p < 0.001*). Percent change in mean papules sig. greater in TTO group (40.1% reduction) compared with placebo group (9.7% reduction), after 45 days (*p < 0.001*). Percent change in mean papules sig. greater in TTO group (47.5% reduction) compared with placebo group (2.4% increase, i.e., worsening), *p < 0.001*.	Minimal pruritus: TTO group 3/30, Placebo group 2/30 Burning sensation: TTO group 1/30, Placebo group 2/30 Minimal scaling: TTO group 1/30, Placebo group 0/30	Authors state no funding support and no conflicts of interest. Acknowledgement that Cinere company provided TTO and placebo products.
Najafi-Taher; 2022; IRCT2015090223864N1 Najafi-Taher et al. (2022)	RCT (parallel); Iran; Hospital clinic	N = 100 enrolled: TTO group = 53, control group = 47 (*n analysed not reported*) Age in years as mean (SD): TTO group 26.7 (5.2), Control group 27.4 (5.0) Female: TTO group 42/53 Control group 28/47	*Inclusion criteria:* Mild-moderate acne vulgaris; age 15–40 years. *Exclusion criteria:* Severe acne (nodules and cysts present); diabetes mellitus, endocrine disorder; taking drugs that can cause acne (e.g., steroids, spironolactone, and finasteride); pregnant or lactating; use of other systemic or topical acne treatment from two week before up until end of study.	Intervention group: TTO + adapalene gel (6% TTO nanoemulsion, *Melaleuca alternifolia*). Other ingredients: 0.1% adapalene, DMSO, Tween 80, Span 80, Ethanol, Carbomer 934 and water. Control group: a marketed adapalene gel containing 0.1% adapalene purchased from Aburaihan pharmaceutical Co., Tehran, Iran. Note: Further details on intervention group treatment sourced here (22). Subjects applied allocated gel once daily to clean, dry, affected areas of skin.	**Number of total lesions Number of inflammatory lesions** (papules, pustules) **Number of non-inflammatory lesions** (open and closed comedones)** Acne severity index** (papules + (2 × pustules) + (comedones∕4)) Timepoints: Baseline, week 4, 8 and 12.	**Number of total lesions, inflammatory lesions and non-inflammatory lesions** all reduced to sig. greater extent in TTO + adapalene group compared adapalene only gel, after 12 weeks (*p < 0.001*) **Acne severity index** reduction sig. greater in TTO + adapalene group compared with adapalene only gel, after 12 weeks (*p < 0.001*). Further, 71.7% patients in TTO + adapalene group achieved success in treatment compared with 6.4% in the adapalene only group.	No serious adverse events in either group. Adverse event most frequently reported in both groups was dryness. Irritation reported by sig. more subjects in adapalene only group compared with TTO + adapalene group at week 4 (*p = 0.005*).	Funding: Tehran University of Medical Sciences, grant No. 94-01-87-28505. Conflict of interest: no data provided.
***Wound healing***								
Cho; 2017; trial not registered Cho and Choi, (2017)	RCT (parallel); Korea; Hospital emergency department	N = 94 enrolled (*n = 94 analysed*) Age in years, mean (SD): Burnshield® = 42.3 (12.5) Tap water = 40.2 (12.0) Burn Cool Spray® = 42.7 (13.8) Female: Burnshield® 21/30, Tap water 17/33, Burn Cool Spray® 22/31	*Inclusion criteria*: age ≥16 years; patients presenting with burn wound within 3 hours of incident; burn area less than 5% of total body surface area. *Exclusion criteria*: chemical burns; hypothermia; analgesic use prior to treatment; neurologic or psychiatric disorder; uncooperative behaviour.	Intervention group: Burnshield® foam dressing (1% TTO, *Melaleuca alternifolia*), Levtrade International, South Africa. Other ingredients: 96% water, gelling agent and polyurethane open cell foam; applied to burn for 20 min. Control group 1: running tap water between 23.9°C and 27.3°C, applied continuously to burn for 20 min. Control group 2: Burn Cool Spray®, T&L Co. Ltd., Korea; applied every 5 min for 20 min.	**Skin surface temperature** (measured using infrared camera held 50 cm away from skin FLIR T420®, FLIR Systems Inc., Danderyd, Sweden). Timepoints: 0 min, 5 min, 10 min, 15 min and 20 min. Pain score (*subjectively self-reported using 10 cm ruler visual analog scale*) Timepoints: Pre-treatment and 20-minutes post-treatment.	**Skin surface temperature** sig. greater median reduction in tap water group (-4°C), compared with Burn Cool Spray® (-0.6°C*, p < 0.05*) and Burnshield® (+0.6°C*, p < 0.05*). Pain score sig. decreased in all groups, although sig. greater reduction observed in tap water group compared with Burnshield®, *p < 0.05*. (Note: pre-treatment pain scores were sig. higher in tap water group at baseline, *p = 0.028*). Note: healing time was on average, two days shorter in patients treated with Burnshield (TTO) compared with other treatment groups, *p = 0.101*.	Not assessed.	Funding: (1) Soonchunhyang University Research Fund and, (2) Regencare Co., Ltd. (Seoul, Korea) who also provided Burn Cool Spray® test product but was not involved in any aspect of study.
Rothenberger; 2016; trial not registered Rothenberge et al. (2016)	RCT (cross-over); Germany; Hospital workplace	N = 20 (*n = 20 analysed*) Age in years: Males mean 27, range 24–38; Females mean 26, range 23–33. Female: 11/20	*Inclusion criteria*: none. *Exclusion criteria*: smoking, vascular diseases, comorbidities (e.g., diabetes mellitus, arterial hypertension), or taking perfusion-altering medication.	Intervention group: TTO solution (5% TTO, *Melaleuca alternifolia*). Other ingredients: 95% saline solution (0.9% Sodium chloride USP), B. Braun Melsungen AG, Germany. Control group 1: Saline solution (0.9% Sodium chloride USP), B. Braun Melsungen AG, Germany. Fore, middle, ring, and little fingers of the right hand randomly allocated one of the four test solutions to be immersed in (duration not specified).	**Perfusion dynamics** of fore, middle, ring, or little finger of right hand (*Oxygen to See by LEA Medizintechnik GmbH, Germany*) Blood flow Haemoglobin oxygenation a.→ Haemoglobin concentration Timepoints: Pre-treatment and 10 min post-treatment.	**Perfusion dynamics** a.→ Blood flow sig. higher in TTO group (+19.0%) compared with saline group (−25.6%), after 10 minutes (*p < 0.05*). a.→ Haemoglobin oxygenation no sig. difference between groups after 10 min (+1.5% vs. −9.3%). b.→ Haemoglobin concentration no sig. difference between groups after 10 min (+1.1% vs. −8.7%).	No adverse reactions reported (i.e., skin irritation, allergic contact dermatitis, redness, pruritus, or rash).	Authors state no funding and no conflicts of interest.
***Seborrheic dermatitis***								
Beheshti Roy; 2014; trial not registered Beheshti Roy et al. (2014)	RCT (parallel); Iran; Hospital clinic	N = 54 enrolled (*n = 42 analysed*) Age in years, mean (SD): TTO group 31 (10) Placebo group 28 (8) Female: TTO group 16/23 Placebo group 13/19	*Inclusion criteria*: Mild-moderate facial seborrheic dermatitis; aged 18–45 years. *Exclusion criteria*: Localised or systemic infection; compromised immune system; definitive cutaneous findings e.g., erythroderma, acne, psoriasis; allergy to lotions or moisturisers; pregnant or lactating; use of products for seborrheic dermatitis within 2 weeks prior to study; treatment with systemic steroids or a medication that causes flushing.	Intervention group: TTO gel (5% TTO, *Melaleuca alternifolia*, containing 41.1% Terpinen-4-ol). Other ingredients: hydroxypropyl cellulose gel provided by Parmoon, Tehran, Iran. Control group: placebo gel (hydroxypropyl cellulose). Subjects applied allocated gel three times daily to affected areas.	**Skin area involvement score** (*dermatologist assessed; subscales erythema, scaling, itching and greasy crusts; scoring 1 = ≤10%, 2 = 11%-30%, 3 = 31%-50%, 4 = 51%-70%, 5 = >70%*). Timepoints: Baseline and at weeks 2 and 4. Patient satisfaction score *(dermatologist assessed; <25% = very bad, bad, no change or little improvement, 26%*−*50% = mild improvement, 51%*−*75% = good improvement, 76%*−*99% = major improvement, 100% = total cure*) Adverse events (*patient reported; limited to allergic irritation or inflammation*) Timepoints: Weeks 2 and 4.	**Skin area involvement score** all subscales (erythema, scaling, itching and greasy crusts) sig. decreased in TTO group after 4 weeks. Scores for all subscales sig. lower in TTO group compared with placebo after 4 weeks (*p < 0.05*). Patient satisfaction score in TTO group was 100% i.e., “total cure” in 9/23 patients after week 2 and 21/23 patients after week 4.	No allergic irritation or inflammation reported in either group.	No data provided. TTO and placebo products provided by Parmoon (a company of Dr Jahangir pharmaceutical and hygienic co., Tehran, Iran).
***Skin inflammation***								
Beikert; 2013; trial not registered Beikert et al. (2013)	RCT (parallel, within patient design); Germany; Hospital clinic	N = 40 enrolled (*n = 40 analysed*) Age in years: mean (SD) 26.4 (7.3), range 19–58 Female: 22/40	*Inclusion criteria*: Healthy, adult, non-smokers, both genders, skin-type II or III (Fitzpatrick classification). *Exclusion criteria*: heightened light-sensitivity, allergic disposition, inflammatory skin conditions, skin infections, recent solarium visit, advanced disease where UV-radiation is contraindicated, known allergy to any of the tested substances, use of anti-inflammatory or immune suppressant medication within 8 weeks prior to study, participation in another clinical trial within 4 weeks prior to study, pregnant or lactating.	Intervention group: TTO ointment (5% TTO, *Melaleuca alternifolia*). Other ingredients: cooling ointment (unguentum leniens) formulated by the hospital clinic pharmacy. Control area 1: UV-radiation only, i.e., no ointment applied. Control area 2: UV-radiated area with 100% cooling cream (unguentum leniens). Control area 3: UV-radiated area with 1% hydrocortisone acetate in cooling ointment applied. Control area 4: UV-radiated area with 0.1% betamethasone valerate in cooling ointment applied. Ointments were applied to a UV-radiated area of skin on one side of the upper back. To test tolerability of the ointments, they were also applied to a non-radiated area of skin on the opposite side of the upper back.	**Erythema intensity index** (measured using Mexameter® and assessed according to Krutmann scale) Timepoints: Baseline, after 24 h and 48 h.	**Erythema intensity index** no sig. difference between UV-radiated TTO test area and control areas 1–3 after 48 h. However, erythema sig. lower in control area 4 (0.1% betamethasone) compared with TTO test area after 48 hours (*p = 0.009*). Increased erythema on non-radiated TTO test area after 48 h (suggesting light skin irritation).	No serious side effects. No allergic contact dermatitis reactions.	Funding: no data provided. Conflict of interest: two authors own patents for coriander oil preparations tested.
Hugo Infante; 2022; trial not registered Hugo Infante et al. (2022)	RCT (parallel); Brazil; Not described	N = 40 (*n analysed not reported*) Age in years, mean (SD): 24.2 (2.5), range 18–28 Female: 0/40	*Inclusion criteria*: skin-type II, III or IV (Fitzpatrick classification); aged 18–28 years. *Exclusion criteria*: not reported.	Intervention group 1: TTO emulsion (2% TTO, *Melaleuca alternifolia*), Ferquímica, Brazil, in vehicle formula was applied (1 mL) daily for 90 days. Intervention group 2: TTO nanoemulsion (2% TTO, *Melaleuca alternifolia*), Ferquímica, Brazil, in vehicle formula was applied (1 mL) daily for 90 days. Nanoemulsion produced by PharmaSpecial, Brazil. Control group: Vehicle formula (distilled water, tapioca starch, corn starch, butyleneglycol, polyglyceryl-6-distearate, glycerin, phenoxyethanol and parabens, argan oil, carrageenan iota, ethylene diamine tetra acetic, and butyl hydroxy toluene) was applied (1 mL) daily for 90 days. All subjects instructed to apply SPF 50 sunscreen daily for 15 days (provided by researchers), to avoid use of other sunscreens and skin care products only using their usual skin care products, and to not wash their face within 2 h prior to experiment. Subjects were held in a room with 20–22°C and 45–55% humidity for 20 min prior to experiment.	Note: all measurements collected from frontal facial region. *Stratum corneum* hydration level (*Corneometer®, Courage + Khazaka electronic GmbH, Germany*) Trans-epidermal water loss (*Tewameter® TM 210, Courage & Khazaka, Germany*) Sebum level on skin surface (*Sebumeter® SM815, Courage & Khazaka, Germany*) Sebum level in infundibulum i.e. active sebaceous glands (Sebufix*®* F16*, Courage & Khazaka, Germany*) RCM (*VivaScope™ 1500, VivaScope GmbH, Germany*) *Stratum corneum* thickness Keratinocyte area Average epidermis thickness Papillary depth Average photoaging score calculated using RCM images taken from four regions (*stratum corneum*, *stratum granulosum*, dermal-epidermal junction and collagen networks); score of 5 = best, 1 = worst. Echogenicity i.e. collagen density in dermal layer (*Dermascan C®, Cortex Technology, Denmark*) Timepoints: Baseline and after 90 days	No sig. differences between intervention groups and control group after 90 days in s*tratum corneum* hydration, Trans-epidermal water loss, sebum level on skin surface or sebum level in infundibulum. RCM parameters: →No sig. differences in S*tratum corneum* thickness between groups. →Average epidermis thickness sig. increased in pure TTO group 1 compared with control group (*p* = 0.02). →Keratinocyte area sig. increased in intervention group 2 compared with control group (*p* = 0.02). →Papillary depth sig. increased in intervention group 1 compared with control group (*p* = 0.005). Average photoaging score (using RCM images) not sig. different between groups. However, sig. improvements were found in the *stratum granulosum* for intervention group 2, compared with control (*p* < 0.05), and in the collagen networks for both intervention groups 1 and 2, compared with control (*p* < 0.05). Echogenicity sig. increased in both intervention group 1 (*p* = 0.02) and intervention group 2 (*p* = 0.03), compared with control group.	Not assessed.	Funding: grants provided by FAPESP and CAPES foundations Conflict of interest: no data provided.
***Dandruff***								
Satchell; 2002; trial not registered Satchell et al. (2002a)	RCT (parallel): Australia; Community	N = 126 enrolled (*n = 125 analysed by ITT analysis)* Age in years: TTO group mean 39, Control group mean 42 Female: TTO group 24/63, Control group 31/62	*Inclusion criteria*: Mild-moderate dandruff; aged ≥ 14 years. *Exclusion criteria*: Severe dandruff (whole scalp score >200); unstable dandruff (change in whole scalp score >50 after washout period); seborrheic dermatitis (face and trunk); psoriasis; diabetes mellitus; immunosuppression; chronic disease not stabilised by medication; anticoagulation or systemic corticosteroid use; TTO hypersensitivity.	Intervention group: TTO shampoo (5% TTO, *Melaleuca alternifolia*); after 2-week washout with Johnson’s Baby Shampoo, the TTO shampoo was applied for three minutes before rinsing, once daily, for four weeks. Control group: placebo shampoo (vehicle shampoo without TTO); after 2-week washout with Johnson’s Baby Shampoo, the placebo shampoo was applied for three minutes before rinsing, once daily, for four weeks.	**Quadrant-area-severity score i.e., whole scalp score** (area of involvement score * severity score) a.→ **Area of involvement score** (measured on a scale from 1-5: 1= <10% involvement, 5 = >70% involvement) a.→ **Severity score** (measured on a scale of 0-3; 0 = normal skin, 3 = marked erythema with thick confluent plates of yellowish white scales. Patient reported scaliness, itchiness, and greasiness (*VAS from 0 = none to 10 = worst ever*) Timepoints: Baseline and after weeks 2 and 4.	**Quadrant-area-severity score (i.e., whole scalp score)** sig. reduction in TTO group (−41.2%) compared with placebo (−11.2%) after 4 weeks (*p < 0.001*). **Area of involvement score** sig. decreased in TTO group (−28.3%) compared with placebo (−12.5%) after 4 weeks (*p < 0.001*). **Severity score** sig. decreased in TTO group (−23.4%) compared with placebo (−2.8%) after 4 weeks (*p = 0.001*). Itchiness sig. reduction in TTO group (−23.0%) compared with placebo (−12.1%) after 4 weeks (*p = 0.031*). Greasiness sig. reduction in TTO group (−25.9%) compared with placebo (−8.2%) after 4 weeks (*p = 0.001*), although greasiness mean score was higher in TTO group (43.1) compared with placebo (31.8) at baseline. Scaliness remained similar between groups (TTO group −25.6% vs. placebo −16.9%; *p = 0.066*).	No serious adverse events reported. TTO group: 3/63 reported adverse events (mild stinging in eyes, mild burning of scalp, mild itching of scalp). Placebo group: 8/62 reported adverse events (pruritus, conjunctivitis, and urticaria)	Funding: Australian Tea Tree Oil Research Institute Authors state no conflicts of interest.

Abbreviations: DMSO, dimethyl sulfoxide; ITT, intention-to-treat (analysis); RCT, randomised controlled trial; RCM, reflectance confocal microscopy; SD, standard deviation; TTO, tea tree oil; UV, ultraviolet; VAS, visual analogue scale.

**TABLE 3:** Characteristics of included studies—Infectious disease.

Author; Year of publication; Trial registration	Study design; Country; Setting	Participant characteristics	Eligibility criteria	Interventions; Comparisons	Outcome measures (*methods*); Timepoints	Main findings	Safety	Funding; Conflict of interest
***Infection control (i.e., hand disinfection)***								
Youn; 2021; Clinical Research Information Service No. KCT0003240 Youn et al. (2021)	RCT (parallel); South Korea; Community	N = 112 enrolled (*n = 106 analysed*) Age in years: not reported Female: not reported *Note: Supplementary file 1 describing participant characteristics not available.*	*Inclusion criteria*: aged 18–60 years. *Exclusion criteria*: skin disease affecting the hands or forearms; an open wound, hangnail, or other skin abnormality; immunosuppressant or antibiotic drug use; sensitivity to TTO confirmed by observation.	Intervention group: TTO disinfectant (10% TTO, *Melaleuca alternifolia*), brand and manufacturer not stated. Other ingredients: TTO added in ratio of 2:2:1:15 of TTO, solubiliser, glycerin and sterile distilled water. Dose of 5 mL applied to participant’s hands. Control group 1: Alcohol-based hand sanitiser (a gel containing 83% ethanol without water). Dose of 2 mL applied to participant’s hands. Control group 2: Benzalkonium chloride-based hand sanitiser (a foam containing benzalkonium chloride without water). Dose of 0.8 mL applied to participant’s hands. Control group 3: No treatment. To contaminate their hands, subjects applied 5 mL of Serratia marcescens (ATCC 14756) evenly on their hands for one minute and allowed their hands to dry for 2 min. This contamination procedure was then repeated a second time. Personnel then dispensed the allocated hand disinfectant into subjects’ hands which they rubbed in for ≥ 30 s.	**Skin surface microbial counts** (*assessed by glove juice sampling procedure, US FDA-Tentative Final Monograph for Healthcare Antiseptics, and using MacConkey agar plate, Asan, Korea incubated at 25°C for 48 h*). **Skin surface organisms** (*assessed as relative light units by adenosine triphosphate concentration on skin using ATP Surface Test kit and a Clean-Trace Luminometer 3M Health Care*). Timepoints: Baseline (i.e. in between 1^st^ and 2^nd^ hand contaminations) and post-treatment. Trans-epidermal water loss (*assessed as g/m2/hr using gpskin Barrier probe, gpower, Korea, with normal values in the range 16*–*20 and higher values indicating greater water loss*). Skin condition (*patient-assessed under 3 categories: skin moistness, skin dryness and skin exfoliation, on 5-point scale: 1 = not at all to 5 = extremely high*). Timepoints: Baseline (prior to 1^st^ hand contamination) and post-treatment.	**Skin surface microbial counts** sig. decrease in TTO group -5.50 (1.90), compared with alcohol group: -2.33 (1.62), benzalkonium chloride group: -0.62 (0.57), and no treatment: +0.07 (0.73), all *p < 0.05*. **Skin surface organisms** sig. decrease in TTO group -0.46 (0.51) compared with no treatment -0.11 (0.32), *p < 0.05*. Trans-epidermal water loss no sig. difference between groups in pre- to post-test changes. Skin condition: →*Skin moisture* sig. difference between groups in pre- to post-test changes (TTO 0.96, alcohol 0.63, benzalkonium chloride 0.96, and no treatment 0.59). →*Skin dryness* sig. difference between groups in pre- to post-test changes (TTO −0.81, alcohol −0.26, benzalkonium chloride −0.85, and no treatment −0.59). →*Skin exfoliation* no sig. difference between groups in pre- to post-test changes. *Note: Supplementary File S2 not available.*	Not assessed.	Funding: National Research Foundation of Korea. Grant Number: NRF-2015R1A1A3A04001441. Authors state no conflicts of interest.
Gnatta; 2021; trial not registered Gnatta et al. (2021)	RCT (cross-over); Brazil; Hospital workplace	N = 15 enrolled (*n = 15 analysed*) Age in years: not reported Female: not reported	*Inclusion criteria*: age 18–65 years; short fingernails. *Exclusion criteria*: visible dryness or lesions on hands; contact with antiseptic soap within 48 h prior to study; allergy to test substance(s); pregnant.	Intervention group: TTO soap (2% TTO, *Melaleuca alternifolia*), TTO met standard ISO 4730:2017. Other ingredients: lauryl ether sulfate sodium, cocoamidopropyl betaine, phenoxyethanol, and parabens). Dose of 1.5 mL used. Control group 1: 0.5% triclosan soap (Rioderm®, Rioquímica, Brazil). Dose of 1.5 mL used. Control group 2: 2.0% chlorhexidine soap (Riohex®, Rioquímica, Brazil). Dose of 1.5 mL used. Control group 3: Soft soap. Dose of 5 mL used. *Hand hygiene:* Prior to hand contamination, all subjects cleaned hands with 5 mL soft soap for 60 s, rinsed with mineral water and dried hands with paper towel. After hand contamination with *E. coli*, subjects washed hands with allocated soap.	**Escherichia coli K12** (*ATCC 14948; assessed as CFU/mL by rubbing fingertips in agar plates containing Tryptic Soy Agar with deoxycholate, which were then incubated at 37°C for 24 h and re-incubated for a further 24 h*)*.* Timepoints: Before contamination and after hand hygiene treatment.	**Escherichia coli K12** CFU/mL log^10^ reduction factor sig. higher in TTO group 4.28 (0.50) compared with Riohex® (3.89 (0.62), *p = 0.006*) and soft soap (3.17 (0.55), *p < 0.001*). No sig. difference compared with Rioderm® wash (4.31 (0.35), *p = 0.661*).	Not assessed.	Funding: Foundation for Research Support of the State of São Paulo (Grant No.2013/23008-7). Authors state no conflicts of interest. Triclosan and chlorhexidine soaps donated by Rioquímica company.
Gnatta; 2013; trial not registered Gnatta et al. (2013)	RCT (cross-over); Brazil; Hospital workplace	N = 15 enrolled (*n = 15 analysed*) Age in years, mean (SD) 31.0 (7.7) Female 11/15	*Inclusion criteria*: age 18–55 years; clean, short fingernails. *Exclusion criteria*: visible dryness or lesions on hands; contact with antiseptic soap within 48 h prior to study; allergy to test substance(s); pregnant.	Intervention group: TTO soap (0.3% TTO, *Melaleuca alternifolia*), Doctornatu® liquid soap, Higinatu, Brazil). Dose of 1.5 mL used. Control group 1: 0.5% triclosan soap (Rioderm®, Rioquímica, Brazil). Dose of 1.5 mL used. Control group 2: Soft soap. Dose of 5 mL used. Control group 3: Soft soap + rinsing with 60% propan-2-ol Doses of 5 mL soft soap and 3 mL propan-2-ol used. *Hand hygiene:* Prior to hand contamination, all subjects cleaned hands with 5 mL soft soap for 60 s, rinsed with mineral water and dried hands with paper towel. After hand contamination with *E. coli*, subjects washed hands with allocated soap.	**Escherichia coli K12** (*ATCC 14948; assessed as CFU/mL* Tryptone Soya Selective Agar (DIFCO®, BD®, Sparks, US) incubated at 37°C for 18-24 h and re-incubated for 24 h. Timepoints: Before contamination and after hand hygiene treatment.	**Escherichia coli K12** CFU/mL log10 reduction factor sig. lower in TTO group (3.89 ± 0.69) compared with soft soap + propan-2-ol group (4.89 ± 0.91, *p = 0.001*). No sig. difference compared with soft soap alone (3.87 ± 1.13, *p = 0.247*). Triclosan group also no sig. difference compared with soft soap (3.59 ± 0.71, *p = 0.2975*)	Not assessed.	Funding: Foundation for Research Support of the State of São Paulo (Grant No.2011/18448-2). Authors state no conflicts of interest.
***Molluscum contagiosum***								
Markum; 2012; ACTRN12610000984099 Markum and Baillie, (2012)	RCT (parallel); United States; Not described	N = 53 enrolled (*n = 53 analysed by ITT*) Age in years, mean (SD) 6.3 (5.1) Female: not reported	*Inclusion criteria*: diagnosis of molluscum contagiosum; ≥ 50th percentile for height and weight; meeting all developmental milestones. *Exclusion criteria*: major disease.	Intervention group: TTO (75% TTO, *Melaleuca alternifolia*), terpinene-4-ol content 40.1% conforming to AS 2782-1985. Other ingredients: high selenium canola oil. Control group 1: TTO + iodine (the same TTO and canola oil as TTO intervention in addition to organically bound iodine. Total iodine concentration = 35 micromolar (US patent #7,311,928 from Naturopathix, Inc., US). Control group 2: iodine (the same organically bound iodine in a vehicle of high selenium canola oil). Subjects applied 4 µL topically to each lesion twice daily for 30 days (or until all lesions resolved).	**> 90% reduction in lesions** (*assessment method not defined*) Adverse events (*parent or caregiver reported*) Timepoints: Baseline and every 10 days until end of trial (30 days)	**> 90% reduction in lesions** occurred in sig. more children in TTO + iodine group (16/19) compared with TTO group (3/18, *P<0.01*) and iodine group (1/16, *P<0.01*) after 30 days. Note: conflict with text which states *‘…4 children [in TTO group] met the 90% reduction criterion’*. No correlation between number of lesions at baseline and intervention group.	Redness ≤ 3 mm radius on lesion site: TTO + iodine = 1 TTO = 1 Iodine = 2 Warm sensation on application: TTO + iodine = 3 TTO group = 4	Funding: The Centre of Excellence in Biomedical Research Boise State University, Idaho, US. Authors state no conflicts of interest.
***MRSA colonisation***								
Blackwood; 2013; ISRCTN65190967 Blackwood et al. (2013)	RCT (parallel); Northern Ireland; Hospital intensive care unit	N = 445 enrolled (*n = 391 analysed*) Age in years, mean (SD) TTO group 57.3 (17.9) Control group 57.1 (19.4) Female TTO group 88/195 Control group 68/196	*Inclusion criteria*: age ≥ 18 years; likely to remain in ICU > 48 hours. *Exclusion criteria*: pregnancy; colonised with MRSA on admission; known sensitivity to TTO; readmissions; enrolment in another Investigative Medicinal Product clinical trial within 30 days prior to study.	Intervention group: TTO body wash (50 mg/g TTO, *Melaleuca alternifolia*), Novabac®, Novasel Australia Pty Ltd. Control group: standard care (Johnson’s Baby Softwash®, Johnson & Johnson) Subjects received ≥ 1 full bed bath daily with their allocated body wash until detection of ICU-acquired MRSA, ICU discharge or death.	**Incident MRSA colonisation** (*MRSA screening specimens obtained from the nose and groin*) Timepoints: Baseline (on admission) and at end of study (i.e., ICU-acquired MRSA, discharge, or death). Incident MRSA bacteraemia Timepoints: colonisation and infection data measured daily Maximum increase in SOFA score Timepoints: Baseline (on admission), then daily.	**Incident MRSA colonisation** no sig. difference between TTO group (17/195, 8.7%) and control group (22/196, 11.2%) on discharge/death (2.5%, *p = 0.50*). Incident MRSA bacteraemia occurred in no patients during the study. Maximum increase in SOFA score no sig. difference between TTO group 1.28 (1.79) and control group 1.44 (1.92) during the study (*P=0.85*). Multiple regression analysis showed no sig. relationship between treatment arm and incident MRSA colonization (odds ratio 0.75, 95% CI 0.36–1.56, *p = 0.445*). *Note: which confounding variables were included in this model is unclear from text and table but likely included SOFA score, length of stay and number of invasive device changes*.	Rash TTO group *n* = 2 (both unrelated to bodywash).	Funding: The Northern Ireland Clinical Research Support Centre Authors state no conflicts of interest.
Caelli; 2000; trial not registered Caelli et al. (2000)	RCT (parallel); Australia; Hospital	N = 30 enrolled (*n = 30 analysed by ITT analysis*) Age in years, TTO group mean 58, range 28–82 Control group mean 74, range 45–87 Female TTO group 10/15 Control group 8/15	*Inclusion criteria*: infected or colonised with MRSA. *Exclusion criteria*: none.	Intervention group: TTO regimen (4% TTO nasal ointment + 5% TTO bodywash), both supplied by Australian Bodycare Pty Ltd. Control group: standard care regimen (2% Mupirocin nasal ointment + Triclosan body wash) Subjects followed allocated topical regimen for at least three days. All patients infected with MRSA received intra-venous vancomycin in addition to the decolonization regimen.	**MRSA decolonisation** (*MRSA screening at nostrils, perianal region, and any sites previously MRSA-positive*) Timepoints: 48 and 96 h after the cessation of topical treatment.	**MRSA decolonisation** achieved in 5/15 (33%) patients in TTO group compared with 2/15 (13%) in control group (*note: no statistical comparison*). MRSA infection remained in 3/15 (20%) of patients in TTO group, compared with 8/15 (53%) in the control group (*note: no statistical comparison*).	TTO nasal ointment: mild swelling of nasal mucosa and acute burning upon application (*numbers not reported*). Triclosan body wash: skin tightness (*reported by one patient*). No adverse events reported for TTO bodywash or mupirocin nasal ointment.	Funding: (1) Cavell Trust, (2) the Rural Industries Research and Development Corporation and (3) Australian Bodycare Pty Ltd. Conflict of interest: no data provided. TTO products provided by study funder (Australian Bodycare Pty Ltd.).
Dryden; 2004; trial not registered Dryden et al. (2004)	RCT (parallel); United Kingdom; Hospital	N = 236 enrolled (*n = 224 analysed*) Age: not reported Female: not reported	*Inclusion criteria*: age ≥ 16 years; colonisation with MRSA. *Exclusion criteria*: known sensitivity to TTO; pregnant or lactating.	Intervention group: TTO regimen (10% TTO cream applied to nostrils three times daily and to skin lesions, wounds and ulcers once a day + 5% TTO body wash used all over body at least once daily), both supplied by Ord River Tea Tree Oil Pty Ltd, Australia. Note: TTO cream also applied to axillae and groin areas as an alternative to the TTO bodywash. Control group: standard care regimen (2% mupirocin nasal ointment i.e., Bactroban®, applied to nostrils three times daily + 4% chlorhexidine gluconate soap used all over body at least once daily + 1% silver sulfadiazine cream applied to open skin lesions and ulcers once daily). Subjects followed allocated regimen for five days.	**MRSA decolonisation** i.e., cleared of MRSA carriage (*defined as an absence of MRSA at nostrils, throat, axillae, groin creases and any open skin lesions at both post-treatment assessment timepoints, i.e., at 2-days and 14-days post-treatment*) Timepoints: Baseline, 2-days after treatment and 14-days after treatment.	**MRSA decolonisation** (carriage clearance) no sig. difference between TTO group 46/110 (41%) compared with control group 56/114 (49%), at 14 days post-treatment (*p = 0.0286*). Nasal carriage clearance sig. lower in TTO group who used 10% TTO cream (36/76) compared with control group who used mupirocin nasal ointment (58/74) at 14 days post-treatment (*p = 0.0001*).	None reported verbally or written in patient medical records.	Authors state no funding and no conflicts of interest. Acknowledgment that Ord River Tea Tree Oil Pty Ltd, Australia, supplied the tea tree preparations.
Lee; 2014; trial not registered Lee et al. (2014)	RCT (parallel); Hong Kong; Residential aged care facility	N = 32 enrolled (*n = 32 analysed*) Age in years, mean (SD) TTO group 81.0 (7.6) Control group 79.4 (6.9) Female TTO group 13/16 Control group 12/16	*Inclusion criteria:* ≥ stage II MRSA-colonized wounds (assessed using pressure ulcer categorization from the National Pressure Ulcer Advisory Panel); open chronic wounds with positivity in MRSA wound culture and a break of skin >6 weeks without progression to healing through normal repair processes. *Exclusion criteria:* peripheral vascular disease; use of systemic or topical antimicrobial agents; clinical signs of infection; more than 105 MRSA bacteria/gram of wound tissue; wounds with undermining or tunnelling; known sensitivity or allergy to TTO.	Intervention group: TTO topical solution (10% TTO, *Melaleuca alternifolia*), NOW Foods Company, US. Other ingredients: 90% medical grade paraffin oil. Control group: saline gauze wound dressing only. Subjects’ wound dressings were changed daily by a trained nurse. The wound was cleaned with a 0.9% saline solution. Subjects allocated to the intervention group then had the TTO solution applied. The wound was then covered with a non-adhesive pad.	**MRSA decolonisation** (*measured as CFU/mL after incubating wound swabs aerobically at 35°C for 18-24 h on MRSASelect™ (BioRad, US) agar plates*). **Wound healing** (*assessed using the Pressure Ulcer Scale for Healing tool 3.0 with scores ranging from 0 to 16, where 0 = completely healed*). Timepoints: Baseline and weeks 1, 2, 3 and 4	**MRSA decolonisation –** wound CFU/mL sig. lower in TTO group compared with control group at: Week 1 (4531 vs 8125, *p ≤ 0.001*) Week 2 (2375 vs 8937, *p ≤ 0.001*) Week 3 (468 vs 9875, *p ≤ 0.001*) Week 4 (93 vs 10312, *p ≤ 0.001*) **Wound healing** sig. better in TTO group compared with control group at: Week 1 (5.5 vs 7.6, *p = 0.005*) Week 2 (5.4 vs 6.9, *p = 0.000*) Week 3 (1.0 vs 5.5, *p = 0.000*) Week 4 (0.0 vs 4.6, *p = 0.000*)	No adverse events reported from use of 10% TTO preparation.	Authors state no funding and no conflicts of interest.
***Oral Candida infection***								
Maghu; 2016; trial not registered Maghu et al. (2016)	RCT (parallel); India; Outpatient clinic	N = 36, although this number omits subjects who dropped out of the study (*n = 36 analysed*) Age in years, mean TTO group: 64.9 Control group 1: 63.2 Control group 2: 64.5 Female TTO group: Control group 1: 4/13 Control group 2: 2/10	*Inclusion criteria*: age 20–60 years; oral fungal infection (diagnosis confirmed by histopathological screening or lesion and any prosthesis used). *Exclusion criteria*: anti-fungal medication use; HIV-positive; high risk patients (e.g., leukemia or lymphoma); radiation therapy.	Intervention group: TTO mouthwash (0.25% or 5 mL TTO, botanical species not stated). Other ingredients: 50 mL water. Subjects swished with mouthwash (55 mL) three times daily after meals and were instructed to avoid eating or drinking for following 30 min. Control group 1: clotrimazole ointment; subjects applied ointment three times daily after meals. Control group 2: conservative treatment (regular cleaning and washing of the prosthesis on a daily basis and removal of prosthesis during the night in case fungal infection turned out to be denture-induced). All subjects instructed not to use any other mouthwashes or oral cleaning aids during the study.	Erythema (*assessment method not defined*) Inflammation (*assessment method not defined*) Fungal hyphae (*assessment method not defined*) Burning sensation (*patient-assessed on VAS)* Timepoints: Baseline and after weeks 1, 2 and 3.	Erythema 89% reduction in TTO group compared with 71% with clotrimazole ointment and 40% with conservative treatment. Inflammation 86% reduction in TTO group compared with 80% with clotrimazole ointment and 67% with conservative treatment. Fungal hyphae 86% reduction in TTO group compared with 100% with clotrimazole ointment and 100% with conservative treatment. Burning sensation 100% reduction in TTO group compared with 100% with clotrimazole ointment and 50% with conservative treatment. *Note: no statistical comparisons were performed.*	No patient reported any adverse effects in relation with the TTO mouthwash.	Funding: no data provided Authors state no conflicts of interest.

Abbreviations: ATCC, american type culture collection; ATP, adenosine triphosphate; CFU, colony forming units; CI, confidence interval; FDA, food and drug administration; ICU, intensive care unit; ISO, international organization for standardization; ITT, intention-to-treat (analysis); MRSA, methicillin-resistant *Staphylococcus aureus*; RCT, randomised controlled trial; SD, standard deviation; SOFA, sequential organ failure assessment; TTO, tea tree oil; US, United States; VAS, visual analogue scale.

**TABLE 4:** Characteristics of included studies—Ophthalmology.

Author; Year of publication; Trial registration	Study design; Country; Setting	Participant characteristics	Eligibility criteria	Interventions; Comparisons	Outcome measures (*methods*); Timepoints	Main findings	Safety	Funding; Conflict of interest
***Demodex infestation***								
Craig 2022; trial not registered Craig et al. (2022)	RCT (cross-over); Canada; Outpatient clinic	N = 30 enrolled (*n = 30 analysed*) Age in years, mean (SD) 33 (Soukoulis and Hirsch, 2004), range 20–59. Female: 18/30	*Inclusion criteria*: ≥ 18 years of age *Exclusion criteria*: history of contact lens wear; surgical procedures within two years prior to study; active ocular infection or corneal disease (other than mild dry eye symptoms, i.e. Ocular Surface Disease Index score ≤ 22); topical or systemic medications, or systemic conditions affecting the eye; abnormal corneal sensitivity (Cochet-Bonnet aesthesiometer measurement < 60mm).	Intervention group 1: TTO-based eyelid wipes (5% *Melaleuca alternifolia*) I-Lid’n Lash® Plus, I-MED Pharma, Canada.Intervention group 2: TTO-based eyelid wipes (*Melaleuca alternifolia* - concentration not stated) Blephadex™ Eyelid Wipes, Lunovus, US. Intervention group 3: TTO-based eyelid wipes (*Melaleuca alternifolia* - concentration not stated) Oust™ Demodex®, OCuSOFT®, US. Intervention group 4: TTO-based eyelid wipes (*Melaleuca alternifolia* - concentration not stated) EyeCleanse™ Eyelid Cleanser Lid Wipes with Tea Tree, Chrissanthie, South Africa. Control group: Sensitive Eyes® Plus Saline Solution, Bausch and Lomb, US. Eyelid cleanser wipe or cotton pad soaked in saline solution was applied onto subjects’ closed eyelids using gentle pressure for 10 cycles of side-to-side motion, at the same time of day (within ± 1 h). Washout was 48-72 h between product application days.	**Tear film stability** (*assessed using either Keratograph 5 M, Oculus Optikgeraete GmbH, Germany, or Medmont Corneal Topographer E300, Medmont International, Australia*) Visual acuity (*six-metre best spectacle-corrected logMAR*) Conjunctival hyperaemia i.e. ocular redess (Keratograph 5 M, Oculus Optikgeraete GmbH, Germany) Timepoints: before and at 10 minutes after product application. Ocular surface staining (*according to Centre for Contact Lens Research staining grading scale*) Timepoints: once at enrolment and then at 10 min after each product application **Ocular discomfort score** (*patient-assessed on scale from 0 = no discomfort up to 10 = maximum tolerable discomfort*). Timepoints: before product application and then every 15 s for 5 min after product application, then every 30 s for a further 5 min Time until comfortable eye-opening (*patient assessed as the time elapsed after product application until they felt they could comfortably open their eyes*)	**Tear film stability** (i.e. tear film break up time in seconds) sig. decreased with EyeCleanse™ from 8.9 ± 4.4 to 5.6 ± 3.1, *p < 0.001*. All other groups showed no sig. change. Visual acuity did not significantly change in any group. Bulbar ocular redness sig. increased after applying Lid’n Lash® (0.4 ± 0.2 to 0.6 ± 0.4, *p < 0.05*), and EyeCleanse™ (0.4 ± 0.3 to 0.9 ± 0.6, *p = 0.005*). Similarly, limbal ocular redness sig. increased after applying EyeCleanse™ (0.3 ± 0.2 to 0.8 ± 0.6, *p = 0.01*). Both corneal and conjunctival staining scores increased from 0/100 to 3/100 10-minutes post application of EyeCleanse™ (both *P<0.05*). No sig. changes observed in other intervention groups. **Ocular discomfort score** was sig. higher than baseline (*p < 0.05*) 150 secs post-application of Lid’n Lash®, 60 s post Blephadex™, 120 s post Oust™ Demodex®, and 195 s post EyeCleanse™. Time until patient-reported comfortable eye-opening was sig. longer with Lid’n Lash® vs. Sensitive Eyes® Plus (6 s vs. 1 s, *p < 0.05*), and with EyeCleanse™ vs. Sensitive Eyes® Plus (30 s vs. 1 s, *p < 0.001*).	Superior and inferior lid wiper epitheliopathy grade (*according to Korb et al.*) increased with EyeCleanse™ only. No other adverse or serious adverse events were recorded.	Partial funding from Canadian Optometric Education Trust Fund. Authors state no conflicts of interest.
Karakurt; 2018; trial not registered Karakurt and Zeytun, (2018)	RCT (parallel); Turkey; Outpatient clinic	N = 135 enrolled (*n = 135 analysed*) Age in years, mean (SD) TTO group 57.52 (14.22) Control group 55.15 (13.97) Female TTO group 40/75 Control group 35/60	*Inclusion criteria*: diagnosed demodectic blepharitis based on clinical and parasitological examinations; history of regular application of eyelash-based treatments for Demodex and regular follow-up at study clinic. *Exclusion criteria*: other ocular or systemic disease; ocular surgery; previous systemic or topical treatment.	Intervention group: TTO eyelid shampoo (7.5% TTO, botanical species not stated) Blefaroshampoo®, Teka, Turkey. Control group: eyelid shampoo without TTO (Blepharitis Shampoo®, Jeomed, Turkey) All subjects washed eyelids with allocated shampoo twice daily for 4 weeks.	**Demodex positivity** (*eight eyelashes removed by epilation method and examined under light microscope were defined as Demodex positive when larva, nymphs, or mature Demodex mites were observed in ≥1 eyelash*). **Average Demodex count** (*calculated by dividing total Demodex count by number of eyelashes on which they were observed*). **Ocular symptoms** (*itching, burning, feeling of a foreign body in the eye, eye redness, and cylindrical dandruff assessed as 0 = no symptoms, 1 = light, 2 = moderate, or 3 = severe*). Timepoints: Baseline and after 4 weeks.	**Demodex positivity** all subjects were Demodex positive at baseline. Number of Demodex positive subjects sig. decreased in TTO group to 48/75 after 4 weeks (*p < 0.001*). Similarly, the number of Demodex positive subjects decreased in control group to (53/60) after 4 weeks (*p = 0.008*). *Full Demodex reduction achieved in 36% of TTO group and 12% of control group.* **Average Demodex count** (*in subjects who did not achieve full reduction in Demodex mites*) sig. decrease within TTO group from 12.46/eyelash to 4.15/eyelash after 4 weeks (*p < 0.001*). Similarly, sig. decrease within control group from 11.98/eyelash to 7.91/eyelash, after 4 weeks (*P=0.024*). **Ocular symptoms** mean score of each symptom sig. decreased within TTO group (all *p < 0.001*). Symptom scores also decreased within control group but all *p > 0.05*.	Not assessed.	Funding: Erzincan University, Coordinator of Scientific Research Projects (TSA-2017-441). Authors state no conflicts of interest.
Koo; 2012; trial not registered Koo et al. (2012)	RCT (parallel); Korea; Hospital clinic	N = 281 enrolled (*n = 160 analysed*) Age in years, mean (SD); range TTO group 53.7 (10.3); 23–85 Control group 55.6 (11.3): 25–85 Female TTO group 70/106 Control group 34/54	*Inclusion criteria*: ocular surface discomfort (e.g., dryness, pruritus, ocular pain, or visual disturbance). *Exclusion criteria*: eye surgery within 6 months prior to study; eyedrop use other than artificial tears; current or prior eyelid scrubbing treatment.	Intervention group: TTO eyelid scrub (TTO, *Melaleuca alternifolia*), Sydney Oil Co, Australia. TTO diluted to either 50% or 10% with mineral oil. Subjects received weekly lid scrubs with 50% TTO dilution in clinic and performed twice daily lid scrubs with 10% TTO dilution performed at home, for one month. Control group: Saline eyelid scrub. Subjects received weekly lid scrubs in clinic and performed twice daily lid scrubs performed by subjects at home, for one month.	**Ocular surface discomfort index** (*patient questionnaire scored between 0 and 100 with higher scores meaning greater ocular discomfort*). **Demodex count** (*number of Demodex mites identified by microscopy in total of eight lashes*). Timepoints: Baseline and after one month.	**Ocular surface discomfort index** sig. decrease in TTO ‘good compliance’ group i.e., scrubbed eyelids >10 times/week, compared with control group (*p = 0.005*), and with TTO ‘poor compliance’ group i.e., scrubbing <5 times/week (*p < 0.001*), after 1 month. **Demodex count** sig. decrease in TTO good compliance group i.e., scrubbing eyelids >10 times/week, compared with control group (*p = 0.003*) and with TTO poor compliance group i.e., scrubbing <5 times/week (*p = 0.019*), after 1 month.	Not reported as measured, but authors reported ocular irritation occurred in 5/106 subjects in TTO group.	Authors state no funding and no conflicts of interest.
Wong; 2019; ACTRN12618001368224 Wong et al. (2019)	RCT (parallel, within-subject design); Australia; Unclear	N = 20 enrolled (*n = 20 analysed*) Age in years, median 63.5; range 48–76 Female 14/20	*Inclusion criteria*: age ≥45 years; similar vision in both eyes; generally healthy. *Exclusion criteria*: active anterior segment disease except blepharitis; ocular or systemic medications that may affect tear film or ocular microbiota started within 3 months prior to study or likely to increase in dose during study; ocular surgery within 6 months prior to study; pregnant or lactating; known allergy to TTO, coconut oil or fluorescein dye; heavy make-up users; epilepsy or migraines triggered by flashing light.	Intervention group: TTO-based eyelid wipes (*Melaleuca alternifolia* - concentration not stated) Blephadex™ Eyelid Wipes, Lunovus, US. Subjects used wipes on one randomly selected eye daily for 1 month. Control group: contralateral eye received no treatment.	**Demodex mite count** (*four lashes epilated from each eye and examined under light microscope*). **Ocular microbiota** – bacterial colony count (*swab of inferior lid margin taken, plated, cultured and CFU/mL counted*). **Bacterial lipase** (*assessed by glycerol monolaurate assay of bacterial culture*). Ocular Surface Disease Index (*patient questionnaire scored between 0 and 100 with higher scores meaning greater ocular discomfort*). Tear film stability (*assessed by Tearscope Plus non-invasive tear break up time*). Tear film lipid layer thickness (*assessed by Lipiview® interferometer*). Tear volume (*measured with Phenol red thread test in millimetres*). Ocular symptoms i.e. itching, dryness and overall discomfort (*assessed using VAS from 0 = no symptoms to 100 = maximum symptoms*). Timepoints: Baseline and after 30 days.	**Demodex mite count** sig. decreased in TTO group (median 0, IQR 2) compared with no treatment (median 2, IQR 4) after 30 days (*p = 0.04*). Ocular symptoms of dryness and overall discomfort both sig. decreased in TTO group, compared with no treatment (*p < 0.05*). No sig. difference in change between groups after 30 days (*p > 0.05*) for other outcomes: **Ocular microbiota** →**Bacterial lipase** →Ocular Surface Disease Index →Tear film stability →Tear film lipid layer thickness →Tear volume →Ocular symptom: itching	No adverse events were reported throughout the study. Slight discomfort upon initial use of eyelid wipes *n* = 1.	Funding: The University of New South Wales Authors state no conflicts of interest.
***Dry eye post cataract surgery***								
Mohammadpour; 2020; IRCT2013111313567N5 Mohammadpour et al. (2020)	RCT (parallel); Iran; Hospital clinic	N = 62 enrolled (*n = 62 analysed*) Age in years, mean (SD) 66.4 (8.8), range 37–82 Female 49/62	*Inclusion criteria*: signs and symptoms of dry eye after phacoemulsification cataract surgery that remained after one-month treatment with artificial tears and 1% betamethasone drops. *Exclusion criteria*: signs and symptoms of dry eye present prior to phacoemulsification cataract surgery; meibomian gland dysfunction; blepharitis; epithelial defects; history of trauma; uveitis; trachoma; prior ocular surgery; contact lenses; systemic disease that may cause dry eye.	Intervention group: Eyelid shampoo (EyeSol®, Novaliq GmbH, Germany containing 1% TTO, *Melaleuca alternifolia*) with additional 5% TTO added + artificial tears (Artelac Eye Drops) + topical steroid drops (Betamethasone Eye Drops 0.1%). Control group: Eyelid shampoo (EyeSol®, Novaliq GmbH, Germany containing 1% TTO, *Melaleuca alternifolia*) + artificial tears (Artelac Eye Drops) + topical steroid drops (Betamethasone Eye Drops 0.1%) Study duration was one month. Frequency and doses of eyelid washes are not reported.	Demodex count (*assessed by microscopic examination*) Refraction Corrected and uncorrected distance visual acuity Schirmer test (*test strip inserted at bottom and at corner of the eye without anaesthesia to measure tear production over five minutes*) Tear break-up time (*calculated by observing fluorescein changes in the ocular surface*) Osmolarity of tears (*measured by TearLab*) Ocular Surface Disease Index (*patient questionnaire scored between 0 and 100 with higher scores meaning greater ocular discomfort*) Timepoints: Baseline and after one month.	Demodex count sig. lower in TTO group (0.94 ± 2.26), compared with control group (2.65 ± 3.34), after one month (*p = 0.024*). Tear break-up time sig. improved in TTO group (8.27 ± 3.91), compared with control group (6.55 ± 2.6), after one month (*p < 0.05*). Osmolarity of tears sig. improved in TTO group (291.12 ± 12.6), compared with control group (306.38 ± 10.15), after one month (*p* = *0.018*). Ocular Surface Disease Index score sig. lower in TTO group (21.87 ± 19.09), compared with control group (31.54 ± 22.57), after one month (*p < 0.05*). No sig. difference between groups in refraction, corrected and uncorrected distance visual acuity or Schirmer test, after one month (*p > 0.05*). *Note: no sig. difference between TTO and control group at baseline for any measured outcome (p > 0.05).*	Not assessed.	Authors state no funding and no conflicts of interest.
***Meibomian gland dysfunction***								
Zarei–Ghanavati; 2021; IRCT20201219049753N1 Zarei-Ghanavati et al. (2021)	RCT (parallel, within-patient design); Iran; Hospital clinic	N = 40 enrolled (*n = 40 analysed*) Age in years, mean (SD) 49.2 (21.2), range 28–70 Female 17/40	*Inclusion criteria*: age 18–70 years; diagnosed MGD (‘mainly’ based on Japanese MGD Working Group criteria). *Exclusion criteria*: systemic medication use affecting tear production; topical medication use (e.g., steroids) within 4 weeks prior to study; ocular surgery; other ocular or systemic disease involving ocular surface (e.g., Sjogren syndrome, chemical damage, radiation to head, etc…); infectious keratoconjunctivitis; contact lenses.	Intervention group: TTO eyelid shampoo (EyeSol®, Novaliq GmbH, Germany containing 1% TTO, *Melaleuca alternifolia*). Control group: Johnson’s® baby shampoo, Johnson & Johnson. Subjects washed eyelashes and eyelid margins daily for 60–90 s using the shampoo allocated to the right/left eye and the other shampoo on the opposite eye, for three months.	5-Item Dry Eye Questionnaire score (*patient questionnaire; higher score = greater dry eye severity*) Meibomian gland expressibility (*scored according to number of glands from which fluid could be expressed: 0=all glands expressible, 1=3-4 glands expressible, 2=1-2 glands expressible, 3=no glands expressible*) Plugging (*i.e., oil droplets on eyelid margin*) Capping (*i.e., elevations of meibomian gland orifices*) Eyelid margins: →Foamy tear →Conjunctival hyperaemia →Telangiectasia Tear break-up time (*measured as interval between last blink and appearance of first dry spots on cornea*) Meibum quality (*scored as 0 = clear fluid, 1 = cloudy fluid, 2 = viscous fluid containing particulate matter, 3 = densely opaque, inspissated, toothpaste-like; then summed across the 8 glands tested*) Trichiasis/distichiasis Oxford staining (*score from 0-15; higher scores indicating more severe staining of cornea*) Schirmer test (*using Schirmer1 test*) Timepoints: Baseline and after 1 and 3 months.	5-Item Dry Eye Questionnaire score sig. decrease in TTO group, compared with control group, after 3 months (*p < 0.001*). Meibomian gland expressibility sig. reduced in TTO group, compared with control group, after 3 months (*p = 0.001*). Plugging sig. reduced in TTO group, compared with control group, after 3 months (*p = 0.001*). Capping sig. reduced in TTO group, compared with control group, after 3 months (*p = 0.050*). Eyelid margins: →Foamy tear sig. reduced in TTO group, compared with control group, after 3 months (*p < 0.001*). →Conjunctival hyperaemia no sig. difference between groups after 3 months (*p = 0.187*). →Telangiectasia sig. decreased in TTO group, compared with control group (*p < 0.001*). Tear break-up time increased to sig. greater extent in TTO group, compared with control group, after 3 months (*p < 0.001*). Meibum quality no sig. difference between groups after 3 months (*p = 0.060*). Trichiasis and distichiasis no sig. difference between groups after 3 months (*p > 0.99*). Oxford staining no sig. difference between groups after 3 months (*p = 0.192*). Schirmer test no sig. difference between groups after 3 months (*p = 0.191*).	More subjects in TTO group, compared with control, complained of ocular irritation (21 vs. 12, *p = 0.002*). No allergic reaction or contact dermatitis observed during the 3-month treatment period.	Authors state no funding and no conflicts of interest.

Abbreviations: CFU, colony forming units; IQR, inter-quartile range; LogMAR, logarithm of the minimum angle of resolution; MGD, meibomian gland dysfunction; RCT, randomised controlled trial; SD, standard deviation; TTO, tea tree oil; US, United States; VAS, visual analogue scale.

**TABLE 5:** Characteristics of included studies—Podiatry.

Author; year of publication; trial registration	Study design; country; setting	Participant characteristics	Eligibility criteria	Interventions; comparisons	Outcome measures (methods); timepoints	Main findings	Safety	Funding; conflict of interest
*Onychomycosis*								
Buck; 1994; trial not registered Buck et al. (1994)	RCT (parallel); United States; Hospital and community outpatient clinics	N = 117 enrolled (*n = 108 analysed*)	*Inclusion criteria*: distal subungual toenail onychomycosis confirmed by culture	Intervention group: TTO (100% TTO, *Melaleuca alternifolia*), Thursday Plantation Inc., US.	Clinical assessment (*clinician assessed toenail with greatest fungal involvement as “full”, “partial” or “no” resolution*)	Clinical assessment—no difference between proportions of subjects in TTO group and control group with full or partial resolution of onychomycosis (24/40 vs. 22/36)	Adverse reactions including erythema, irritation and oedema were experienced by: TTO group 5/64	Funding: Schering-Plough Corp, US and Thursday Plantation Inc. US
		Age in years, mean	Exclusion criteria: topical agent applied to toenails within 2 weeks prior to study; immunosuppressant drug use within 6 months prior to study; history of psoriasis; known HIV infection	Subjects applied twice daily for 6 months. *Note: dose not stated*	Timepoints: Baseline and at 1, 3 and 6 months	Fungal culture test—no difference between proportions of subjects with negative culture in TTO group and control group (7/39 vs. 4/36)	Control group 3/53	Conflicts of interest: no data provided
		TTO group 61		Control group: 1% clotrimazole solution, Schering-Plough Corp, US.	Fungal culture test (*assessed by dermatophyte infection test medium*)	Subjective assessment—no difference in proportion of subjects in TTO group and control group reporting resolution or improvement 3 months after discontinuation of therapy (33/59 vs. 27/49)		
		Control group 59		Subjects applied twice daily for 6 months. *Note: dose not stated*	Timepoints: Baseline and post-treatment (i.e., at 6 months)	*Note: no statistically sig. differences found between the two treatments using chi-square statistic*		
		Female TTO group 49/64		At 1-, 3- and 6-month assessments subjects’ toenails were trimmed and debrided	Subjective assessment (*patient-reported appearance and symptoms i.e., pruritis and pain, as resolved, improved, stayed the same or worsened*)			
		Control group 38/53			Timepoint: 3 months after cessation of treatment (i.e., after 9 months)			
Tinea pedis								
Satchell; 2002; trial not registered Satchell et al. (2002b)	RCT (parallel); Australia; Community	N = 137 enrolled (*n = 120 analysed*)	Inclusion criteria: intertriginous tinea pedis; age ≥14 years; dermatophyte infection evident from microscopy examination	Intervention group 1	Mycological cure (*assessed by culture of skin scrapings*)	Mycological cure (i.e., negative culture) was achieved in sig. greater proportion of subjects in 50% TTO group (23/36) and 25% TTO group (18/33), compared with placebo group (14/45), after 4 weeks (*p < 0.01*)	Moderate to severe dermatitis occurred in one patient applying 25% TTO and 3 patients applying 50% TTO (this improved on stopping the study medication)	Funding: Australian Tea Tree Oil Research Institute, Australia
		Age in years, range 17–83; mean TTO 50% group 45	Exclusion criteria: systemic antifungal drug use within 6 months prior to study; topical antifungal drug use within 7 days prior to study; dermatitis; immune-suppressed; known hypersensitivity to TTO.	50% TTO solution (50% TTO, *Melaleuca alternifolia*); mixed with ethanol and polyethylene glycol	Timepoints: Baseline and after 4 weeks	Clinical signs and symptoms—marked improvement achieved in sig. greater proportion of subjects in 50% TTO group (26/38) and 25% TTO group (26/36), compared with placebo group (18/46), after 4 weeks (*p < 0.005*)	Stinging (burning) on application reported by 2 subjects in 25% TTO group and 2 subjects in placebo group	Conflicts of interest: no data provided
		TTO 25% group 38		Intervention group 2: 25% TTO solution (25% TTO, *Melaleuca alternifolia*); mixed with ethanol and polyethylene glycol	Clinical signs and symptoms (*two investigator-assessed items: scaling and inflammation and two patient-assessed items: burning and itching, were each scored separately as 0 = absent, 1 = mild, 2 = moderate, 3 = severe, 4 = very severe. All four items were then summed. Marked improvement was defined as a final clinical score of 0 or reduction of ≥ 3 to a final value < 3*)			
		Control group 39		Control group: placebo (20% ethanol, 80% polyethylene glycol)	Timepoints: Baseline and after weeks 2 and 4			
		Female TTO 50% group 14/51		Subjects applied allocated solution twice daily for 4 weeks. All subjects washed feet with soap and water, dried and then applied their allocated solution. Subjects were instructed not to use other antifungal treatments during the study				
		TTO 25% group 20/54						
		Control group 20/53						
Tong; 1992; trial not registered Tong et al. (1992)	RCT (parallel); Australia; Not described	N = 121 enrolled (*n = 104 analysed*)Age in years, median (range)TTO group 30 (18–60)Tolnaftate group 30 (20–57)Placebo group 34 (19–65)Female TTO group 13/37Tolnaftate group 11/33Placebo group 1/34	Inclusion criteria: diagnosed tinea pedis (based on microscopy examination or fungal culture); age 16–65 yearsExclusion criteria: systemic antifungal drug use within 6 months prior to study; topical antifungal drug use within 1 week prior to study; medical condition or medication use increasing risk for fungal infection	Intervention group: TTO cream (10% w/w TTO, *Melaleuca alternifolia*). Other ingredients: sorbolene creamTTO cream produced by Pharmaco Pty Ltd., AustraliaControl group 1: 1% tolnaftate cream (Tinaderm^®^, Schering Pty Ltd., Australia)Control group 2: placebo (sorbolene cream—vehicle cream)All subjects applied allocated cream twice daily for 4 weeks	Mycological cure (*assessed by culture of skin scrapings*)Timepoints: Baseline and at week 4 (i.e., end of treatment)Clinical signs and symptoms (*two investigator-assessed items: scaling and inflammation and two patient-assessed items: burning and itching, were each scored separately as 0 = absent, 1 = mild, 2 = moderate, 3 = severe, 4 = very severe. All four items were then summed. Clinical improvement defined as an improvement in clinical score > 2 points*)Timepoints: Baseline and at weeks 1, 2, 3 and 4 (i.e., end of treatment)	Mycological cure (i.e., negative culture) achieved in sig. greater proportion of subjects in tolnaftate group (28/33) compared with TTO group (11/37) and placebo group (7/34), after 4 weeks (*p < 0.001*). Difference not sig. between TTO group and placebo, after 4 weeks (*p = 0.393*)Clinical signs and symptoms sig. improvement in TTO group (24/37), compared with placebo group (14/34), after 4 weeks (*p = 0.022*)	Mild erythema developed in one subject from tolnaftate groupNo patient dropped out due to adverse side effects	Funding: Australian Rural Industries Research and Development Corporation and the Australian Tea Tree Oil Industry AssociationConflicts of interest: no data provided

Abbreviations: HIV, human immunodeficiency virus; RCT, randomised controlled trial; TTO, tea tree oil; US, United States.

**TABLE 6:** Characteristics of included studies—Other.

Author; year of publication; trial registration	Study design; country; setting	Participant characteristics	Eligibility criteria	Interventions; comparisons	Outcome measures (methods); timepoints	Main findings	Safety	Funding; conflict of interest
Anxiety and sleep quality								
Ozkaraman; 2018; trial not registered Ozkaraman et al. (2018)	RCT (parallel); Turkey; Outpatient clinic	N = 70 enrolled (*n analysed not reported*)	Inclusion criteria: cancer diagnosis; age ≥ 18 years; able to smell; weekly chemotherapy with paclitaxel	Intervention group: TTO (botanical species not stated, “commercially available”—not further described)	Anxiety (*State-Trait Anxiety Inventory—Turkish version, consisting of two sub-scales: State Anxiety and Trait Anxiety*)	State Anxiety no sig. difference in change scores across groups (F = 0.826, *p = 0.442*), after chemotherapy	Not assessed	Funding: Scientific Research Project Coordination Unit at Eskişehir Osmangazi University
		Age in years, mean (SE) 58.2 (12.8)	Exclusion criteria: chronic disease; psychiatric illness; history of allergies; anxiolytic drug use	Three drops on piece of cotton placed on subjects’ neck and shoulders 10-inches from nose	Sleep quality (*Pittsburgh Quality Sleep Index—Turkish version. Total scores range 0–21. Scores < 5 indicate good sleep quality. Scores > 5 points indicate poor sleep quality*)	Trait Anxiety sig. difference in change scores across groups (F = 11.002, *p < 0.001*). Only lavender sig. decreased trait anxiety (*p < 0.001*)		Conflicts of interest: no data provided
		Female 59/70		Control group 1: Lavender essential oil (*Lavandula hybrida*)	Timepoints: Baseline (prior to 1st cycle chemotherapy) and after completing chemotherapy treatment	In post-hoc analyses, the decrease in lavender essential oil group was sig. greater than TTO group (*p = 0.046*), as TTO did not sig. change trait anxiety		
				Three drops on piece of cotton placed on subjects’ neck and shoulders 10-inches from nose		**Sleep quality** sig. difference in change scores across groups (F = 8.991, *p < 0.001*). Score sig. decreased within both lavender and TTO groups (*p < 0.001*)		
				Control group 2: no treatment				

Abbreviations: RCT, randomised controlled trial; SE, standard error; TTO, tea tree oil.

### 3.2 Dentistry

Eighteen trials were published from 2002 to 2022 and were conducted in Australia (Soukoulis and Hirsch, 2004), Brazil (Casarin et al., 2019), Chile (Catalán et al., 2008), Egypt (Elgendy et al., 2013; Taalab et al., 2021), India (Groppo et al., 2002; Prabhakar et al., 2009; Shetty et al., 2013; Chandrdas et al., 2014; Raut and Sethi, 2016; Bharadwaj et al., 2020; Kamath et al., 2020; Reddy et al., 2020; Srikumar et al., 2022), Italy (Salvatori et al., 2017; Ripari et al., 2020), Switzerland (Saxer et al., 2003), or United Arab Emirates (Rahman et al., 2014), within a primary, secondary or tertiary education setting or within a hospital or outpatient clinic setting.

#### 3.2.1 Oral hygiene practices

Oral hygiene practices focus on the use of tooth brushing, interdental aides, and mouthwashes to limit the accumulation of microbial plaque. Twelve trials tested the efficacy of tea tree oil for control of microbial plaque on the tooth surface (Groppo et al., 2002; Saxer et al., 2003; Soukoulis and Hirsch, 2004; Prabhakar et al., 2009; Chandrdas et al., 2014; Rahman et al., 2014; Salvatori et al., 2017; Casarin et al., 2019; Bharadwaj et al., 2020; Kamath et al., 2020; Reddy et al., 2020; Ripari et al., 2020). Outcomes assessed in these trials included plaque indices or scores (Saxer et al., 2003; Soukoulis and Hirsch, 2004; Rahman et al., 2014; Salvatori et al., 2017; Casarin et al., 2019; Bharadwaj et al., 2020; Reddy et al., 2020; Ripari et al., 2020), gingival indices (Soukoulis and Hirsch, 2004; Salvatori et al., 2017; Bharadwaj et al., 2020; Kamath et al., 2020; Reddy et al., 2020; Ripari et al., 2020), bleeding indices or scores (Saxer et al., 2003; Soukoulis and Hirsch, 2004; Rahman et al., 2014; Salvatori et al., 2017; Ripari et al., 2020), salivary *Streptococcus mutans* (*S. mutans*) count (Groppo et al., 2002; Prabhakar et al., 2009; Chandrdas et al., 2014; Kamath et al., 2020) or, in one study, salivary *Lactobacillus* count (Prabhakar et al., 2009). Only one study measured gingival crevicular fluid as a biomarker of gingival inflammation (Casarin et al., 2019), while two measured dental discoloration (Salvatori et al., 2017; Ripari et al., 2020), and another measured probing pocket depth (Ripari et al., 2020). Ten trials tested a tea tree oil-based mouthwash for controlling microbial plaque (Saxer et al., 2003; Rahman et al., 2014; Salvatori et al., 2017; Casarin et al., 2019; Bharadwaj et al., 2020; Kamath et al., 2020; Reddy et al., 2020; Ripari et al., 2020) and/or salivary microorganisms (Groppo et al., 2002; Prabhakar et al., 2009; Kamath et al., 2020). Of these, two tested Tebodont^®^ (Saxer et al., 2003; Rahman et al., 2014), while others were either formulated for research containing 0.2%–0.5% tea tree oil or, in one study, the tea tree oil was diluted by participants themselves (Ripari et al., 2020).

Three trials tested a tea tree oil-based mouthwash in children aged between 8 and 15 years (Prabhakar et al., 2009; Kamath et al., 2020; Reddy et al., 2020). Kamath et al. (2020), compared the efficacy of a 0.5% tea tree oil mouthwash with three other mouthwashes (one based on aloe vera and peppermint oil, a chlorhexidine mouthwash and a placebo, i.e., distilled water) in 152 subjects aged 8–14 years (Kamath et al., 2020). After 4 weeks, dental plaque, gingival inflammation, and *S. mutans* count significantly decreased with twice daily use of the tea tree oil, aloe vera, and chlorhexidine-based mouthwashes compared with placebo (*p* < 0.001). No differences were observed in these parameters between the tea tree oil, aloe vera and chlorhexidine mouthwashes (Kamath et al., 2020). Prabhakar et al. (2009), also tested a 0.2% tea tree oil mouthwash on *S. mutans* and *Lactobacillus* counts in 36 children aged 9–11 years (Prabhakar et al., 2009). The tea tree oil mouthwash reduced both *S. mutans* and *Lactobacillus* counts after 7 days of using the mouthwash twice daily after meals. Counts of these micro-organisms remained significantly lower than baseline after 14 days (i.e., 7 days after ceasing use of tea tree oil mouthwash). These results however, were not compared to the placebo group, for which, the authors reported no data (Prabhakar et al., 2009). Lastly, Reddy et al. (2020), compared the effects of a 0.2% tea tree oil mouthwash with a chlorhexidine mouthwash or placebo (*not defined*) on plaque and gingival indices in children (Reddy et al., 2020). After 15 days, both dental plaque and gingival inflammation decreased with use of both tea tree oil and chlorhexidine mouthwashes (Reddy et al., 2020). Compared with chlorhexidine, the tea tree oil mouthwash was more effective in reducing gingival inflammation (mean difference −0.30 [−0.47, −0.13], based on re-analysis of data (see Tables 1–6). Compared with placebo, the tea tree oil mouthwash was more effective at reducing dental plaque.

Adverse effects were only assessed in two of these trials (Prabhakar et al., 2009; Reddy et al., 2020). Children using a 0.2% tea tree oil mouthwash reported unpleasant taste, burning sensation, bad breath and nausea, although these effects were more frequently reported with use of a garlic mouthwash (Prabhakar et al., 2009). In the other trial, 1/30 children reported taste changes and burning sensation with use of a 0.2% tea tree oil mouthwash (Reddy et al., 2020).

Seven trials tested tea tree oil-based mouthwashes in adults 18 years of age or older (Groppo et al., 2002; Saxer et al., 2003; Rahman et al., 2014; Salvatori et al., 2017; Casarin et al., 2019; Bharadwaj et al., 2020; Ripari et al., 2020). Bharadwaj et al. (2020), compared twice daily rinses with a tea tree oil-based mouthwash, to Freshclor (a chlorine dioxide-based mouthwash) or Guard-OR (a chlorhexidine-based mouthwash) for 3 weeks in 60 adults aged 18–25 years (Bharadwaj et al., 2020). All mouthwashes reduced both dental plaque accumulation and gingival inflammation after 3 weeks. However, tea tree oil was more effective in reducing dental plaque accumulation than Freshchlor, but less effective in reducing gingival inflammation compared with Guard-OR. Casarin et al. (2019), compared twice daily rinses with a mouthwash containing 0.3% tea tree oil nanoparticles or with Periogard^®^ (a chlorhexidine gluconate-based mouthwash) in 60 adult subjects. Participants using Periogard^®^ had less dental plaque after 4 days than those using the tea tree oil mouthwash on both biofilm free and biofilm covered surfaces (both *p <* 0.05). However, as this parameter was not measured at baseline, it is not possible to know whether those in the Periogard^®^ group had less dental plaque at baseline. No significant difference was observed in gingival crevicular volume between the two groups. Groppo et al. (2002), tested the anti-microbial effect against *S. mutans* of a 0.2% tea tree oil mouthwash, compared with a 2.5% garlic mouthwash or 0.12% chlorhexidine mouthwash in 30 adults aged 18–35 years (Groppo et al., 2002). After 1 week of rinsing with their allocated mouthwash, salivary *S. mutans* count significantly decreased within all groups. Use of either the TTO or garlic mouthwashes resulted in significantly lower *S. mutans* in the 2 weeks after ceasing mouth rinsing (Groppo et al., 2002). Rahman et al. (2014), compared twice daily rinses with Tebodont^®^ (a tea tree oil-based mouthwash) with a 0.05% cetylpyridinium chloride mouthwash (Aquafresh^®^), a 0.12% chlorhexidine mouthwash (Oro-Clense^®^), or a placebo (colored water) (Rahman et al., 2014). After 5 days using their allocated mouthwash, Tebodont^®^ reduced dental plaque, although this reduction was not significantly different to placebo. Oro-Clense^®^ was more effective at reducing plaque compared with Tebodont^®^ (*p* = 0.019). Gingival bleeding was found to decrease to a similar extent with all mouthwashes (including placebo). Salvatori et al. (2017), also tested Tebodont^®^, comparing this to a 0.12% chlorhexidine mouthwash, an essential oil mouthwash (without tea tree oil) and a placebo mouthwash (i.e., red food dye in water) in 16 participants aged 21–37 years (Salvatori et al., 2017). Participants used their allocated mouthwash for 2 weeks—frequency of use was not reported. No significant differences were observed between groups in dental plaque, bleeding, or gingival inflammation after 2 weeks (Salvatori et al., 2017). Saxer et al. (2003), was the third study to test Tebodont^®^, comparing this to a placebo mouthwash without TTO provided by the same manufacturer in 30 subjects aged 18–65 years (Saxer et al., 2003). Participants rinsed three times daily with their allocated mouthwash for 12 weeks. No differences were observed in plaque or bleeding indices between groups after 12 weeks Ripari et al. (2020), compared a tea tree oil mouthwash used 1–3 times daily with a 0.12% chlorhexidine mouthwash used twice daily, for 14 days in 42 subjects aged 18–60 years (Ripari et al., 2020). Participants diluted the tea tree oil mouthwash themselves and were instructed to use a total of 9 drops per day, hence the variable mouthwash frequency for this group. The TTO mouthwash produced significantly greater reductions in both gingival inflammation and probing depth compared with the chlorhexidine mouthwash (*p* < 0.001). However, both mouthwashes reduced dental plaque and gingival bleeding to a similar extent (Ripari et al., 2020).

Adverse effects of tea tree oil mouthwashes in adult participants were assessed in all but one study (Bharadwaj et al., 2020), while a further study reported no participants to have experienced a “serious adverse events or side effects” (Casarin et al., 2019). Of the remaining studies, the following adverse effects were reported: burning sensation (Groppo et al., 2002; Rahman et al., 2014), bitter taste (Rahman et al., 2014), altered taste perception (Salvatori et al., 2017), nausea (Ripari et al., 2020), and minor changes to the oral mucosa (Saxer et al., 2003). Both burning sensation and bitter taste were also reported by intervention groups using a mouthwash not based on tea tree oil (Groppo et al., 2002; Rahman et al., 2014), and burning sensation was reportedly more intense with a garlic-based mouthwash than with the tea tree oil mouthwash tested (Groppo et al., 2002). While 4/22 participants using a tea tree oil mouthwash reported nausea, 2/22 participants in this group were pregnant (Ripari et al., 2020).

Rather than targeting salivary micro-organisms, Chandrdas et al. (2014), tested a tea tree oil solution for sanitizing toothbrushes in 210 subjects aged 18–25 years (Chandrdas et al., 2014). Participants brushed their teeth with a sterile toothbrush twice daily for 2 weeks, after which toothbrushes were either treated with a UV toothbrush sanitizing device or immersed for 12 h in one of the following sanitizing solutions: 0.2% tea tree oil solution, 3% fresh garlic solution, 0.2% chlorhexidine solution, 0.05% cetylpyridinium chloride solution, or distilled water. All treatments significantly reduced the count of *S. mutans* after 2 weeks, including the distilled water. However, any between group comparisons were compromised by the significant difference in *S. mutans* counts observed between groups at baseline (*p* < 0.001) (Chandrdas et al., 2014). Finally, Soukoulis et al., 2004, tested a 2.5% tea tree oil gel which was used as a toothpaste to brush gingival tissue in 58 adult subjects with moderate to severe gingivitis (Soukoulis and Hirsch, 2004). Twice daily brushing with a 2.5% tea tree oil gel was compared with Perioguard^®^ and a placebo gel (same vehicle gel as tea tree oil gel, without tea tree oil), for 8 weeks (Soukoulis and Hirsch, 2004). No differences were found between the gels in gingival inflammation or staining score. During the trial no adverse reactions to the tea tree oil gel were reported (Soukoulis and Hirsch, 2004).

#### 3.2.2 Periodontitis

Three studies tested the application of a tea tree oil-based gel to the periodontium as a treatment for periodontitis (Elgendy et al., 2013; Raut and Sethi, 2016; Taalab et al., 2021). Two trials tested a 5% tea tree oil-based gel applied to the periodontal pocket in addition to scale and root planing (SRP) and compared this to SRP alone (Elgendy et al., 2013; Taalab et al., 2021). Elgendy et al. (2013), found the 5% tea tree oil-based gel + SRP was superior to SRP alone for reducing gingival inflammation, probing pocket depth and clinical attachment loss among 40 subjects aged 30–60 years (Elgendy et al., 2013). Similarly, Taalab et al. (2021), found application of a 5% tea tree oil gel, adjunctive to SRP, significantly reduced clinical attachment level, gingival inflammation, bleeding on probing and level of matrix metalloproteinase-8 in the gingival crevicular fluid (a biomarker of local periodontal disease), after 6 months in 30 subjects aged 25–50 years (Taalab et al., 2021). Raut and Sethi (2016), compared three gel treatments (i.e., 5% tea tree oil gel, a Co-Q10 gel, or a placebo gel) within each patient by using a ‘split mouth’ study design (Raut and Sethi, 2016). Here, three sites in the periodontium affected by periodontitis were identified for each patient and one of the treatment gels was randomly applied to each of these three affected sites, such that each patient received all three treatment gels. All participants also received SRP as a co-intervention. Dental plaque and gingival inflammation decreased within all groups, although probing pocket depth and clinical attachment level decreased only within the tea tree oil and Co-Q10 groups. Adverse effects only assessed in one study (Taalab et al., 2021), where no participants reported an adverse reaction, and some (number not provided) reported an unpleasant taste from the tea tree oil gel applied.

#### 3.2.3 Denture stomatitis

Catalán et al. (2008), compared a Coe-Comfort tissue conditioner with added tea tree oil to the same Coe-Comfort tissue conditioner alone or with the anti-fungal medicine Nystatin added, on growth inhibition of *Candida albicans* on the palate mucosa (Catalán et al., 2008). Both tea tree oil and Nystatin significantly reduced *C. albicans* count after 12 days, compared with Coe-Comfort alone. Palatal inflammation also significantly decreased with both tea tree oil and Nystatin, compared with Coe-Comfort alone. Adverse events were not assessed.

#### 3.2.4 Halitosis

Srikumar et al. (2022), compared a tea tree oil based mouthwash to a chlorhexidine mouthwash (Maxxio^®^) and placebo mouthwash, for treatment for oral halitosis (Srikumar et al., 2022). Halitosis was measured subjectively by full mouth organoleptic scoring and objectively by presence of Volatile Sulfur Compounds (VSCs). Further, presence of *Solobacterium moorei* (*S. moorei*) bacteria on the tongue has been positively correlated with oral halitosis. In this study, researchers found 100% of participants diagnosed with oral halitosis (*n* = 120) had *S. moorei* bacteria in their saliva and on the posterior tongue surface, compared with 17% and 24%, respectively, of participants who did not have oral halitosis (*n* = 40). Participants with halitosis (*n* = 120) rinsed daily with 30 ml of their allocated mouthwash for 1 week. A significantly greater reduction in *S. moorei* count in saliva and on tongue was found for the tea tree oil and chlorhexidine mouthwashes, compared with the placebo group—which showed no change in *S. moorei* counts. Both organoleptic score and VSCs also significantly decreased with the tea tree oil and chlorhexidine mouthwashes relative to placebo—which showed no change in these parameters after 1 week. Researchers noted no adverse effects of the mouthwashes (Srikumar et al., 2022).

#### 3.2.5 Patient-clinician cross-contamination

Shetty et al. (2013), compared a tea tree oil mouthwash (Emoform^®^—same manufacturer as Tebodont^®^) to a chlorhexidine mouthwash (Rexidine^®^) or distilled water, for reducing the bacterial count of dental aerosol (i.e., in patients’ breath) released during ultrasonic tooth scaling (Shetty et al., 2013). The purpose was to investigate prophylactic mouthwashes that may be used to limit patient-to-clinician cross infection during such procedures. Emoform^®^ was more effective in reducing the total Colony Forming Units per milliliter (CFU/ml) in dental aerosol compared with distilled water (*p* < 0.001). However, Rexidine^®^ was found to be more effective than Emoform^®^ for this parameter (*p* < 0.001). Safety of the mouthwashes was not assessed.

#### 3.2.6 Risk of bias

All domains were assessed as low risk or unclear risk (Table 7), except for two studies assessed as high risk for “other bias” (Ripari et al., 2020; Srikumar et al., 2022). These two trials were assessed as high risk due to conflicting data within published article and between trial registry and published article (Srikumar et al., 2022), or participants diluting their own tea tree oil mouthwash without an assessment of compliance (Ripari et al., 2020). Selection bias due to use of a non-random or quasi-random allocation sequence was assessed as low risk in 11 of the 18 dentistry studies; the remainder assessed as unclear risk as the method for randomizing participants was not defined (Saxer et al., 2003; Soukoulis and Hirsch, 2004; Catalán et al., 2008; Prabhakar et al., 2009; Chandrdas et al., 2014; Salvatori et al., 2017; Kamath et al., 2020). Selection bias arising from inadequate concealment was assessed as low risk in nine of 18 studies; the remainder as unclear risk largely due to providing no information on methods used for allocation concealment (Groppo et al., 2002; Saxer et al., 2003; Soukoulis and Hirsch, 2004; Catalán et al., 2008; Prabhakar et al., 2009; Elgendy et al., 2013; Shetty et al., 2013; Chandrdas et al., 2014; Ripari et al., 2020). Five of ten studies measuring objective outcomes (Groppo et al., 2002; Prabhakar et al., 2009; Elgendy et al., 2013; Taalab et al., 2021; Srikumar et al., 2022), and eight of 13 studies measuring subjective outcomes (Saxer et al., 2003; Elgendy et al., 2013; Rahman et al., 2014; Salvatori et al., 2017; Reddy et al., 2020; Ripari et al., 2020; Taalab et al., 2021; Srikumar et al., 2022), were assessed as unclear risk for performance bias due to 1) lack of information on blinding, or 2) potential breaking of blinding and this potentially influencing participant and/or personnel behavior with regards to the intervention(s), e.g., it was plausible that participants could detect TTO and this may have influenced their behavior on-trial. All studies measuring objective outcomes were assessed as low risk for detection bias, as were seven of 13 studies measuring subjective outcomes (Table 7). The remainder were assessed as unclear risk, due to lack of information on outcome assessor blinding. Studies assessed as low risk for attrition bias (8/18) either clearly reported attrition within results text or flow diagram, reported that all participants completed the study, or were of short enough duration that attrition was highly unlikely, e.g., conducted all assessments before and after an ultrasonic tooth scaling procedure (Shetty et al., 2013). Only three studies had been registered on a public trial registry platform (Casarin et al., 2019; Taalab et al., 2021; Srikumar et al., 2022), and were therefore assessed as having low risk for reporting bias; all other trials were assessed as unclear risk for this domain. Finally, 2 out of 18 studies were assessed as high risk for other bias due to concerns about the study design, and the data validity. Another 9 of the 18 studies were assessed as unclear risk for other bias due to insufficient reporting of study design, or methodology.

**TABLE 7:** Risk of bias assessment of the included studies—Dentistry.

Bias author (year)	Random sequence generation (selection bias)	Allocation concealment (selection bias)	Blinding of participants and personnel (performance bias)		Blinding of outcome assessment (detection bias)		Incomplete outcome data (attrition bias)	Selective reporting (reporting bias)	Other bias
			Objective outcomes	Subjective outcomes	Objective outcomes	Subjective outcomes			
Bharadwaj et al. (2020)	Low risk	Low risk	N/A	Low risk	N/A	Low risk	Low risk	Unclear risk	Low risk
Casarin et al. (2019)	Low risk	Low risk	Low risk	Low risk	Low risk	Low risk	Low risk	Low risk	Unclear risk
Catalán et al. (2008)	Unclear risk	Unclear risk	Low risk	N/A	Low risk	N/A	Unclear risk	Unclear risk	Unclear risk
Chandrdas et al. (2014)	Unclear risk	Unclear risk	Low risk	N/A	Low risk	N/A	Unclear risk	Unclear risk	Low risk
Elgendy et al. (2013)	Low risk	Unclear risk	Unclear risk	Unclear risk	Low risk	Unclear risk	Low risk	Unclear risk	Low risk
Groppo et al.(2002)	Low risk	Unclear risk	Unclear risk	N/A	Low risk	N/A	Unclear risk	Unclear risk	Unclear risk
Kamath et al. (2020)	Unclear risk	Low risk	Low risk	Low risk	Low risk	Unclear risk	Unclear risk	Unclear risk	Unclear risk
Prabhakar et al. (2009)	Unclear risk	Unclear risk	Unclear risk	N/A	Low risk	N/A	Unclear risk	Unclear risk	Unclear risk
Rahman et al. (2014)	Low risk	Low risk	N/A	Unclear risk	N/A	Low risk	Low risk	Unclear risk	Low risk
Raut and Sethi (2016)	Low risk	Low risk	N/A	Low risk	N/A	Unclear risk	Unclear risk	Unclear risk	Unclear risk
Reddy et al. (2020)	Low risk	Low risk	N/A	Unclear risk	N/A	Unclear risk	Unclear risk	Unclear risk	Unclear risk
Ripari et al. (2020)	Low risk	Unclear risk	N/A	Unclear risk	N/A	Unclear risk	Unclear risk	Unclear risk	High risk
Salvatori et al. (2017)	Unclear risk	Low risk	N/A	Unclear risk	N/A	Low risk	Unclear risk	Unclear risk	Unclear risk
Saxer et al. (2003)	Unclear risk	Unclear risk	N/A	Unclear risk	N/A	Low risk	Low risk	Unclear risk	Low risk
Shetty et al. (2013)	Low risk	Unclear risk	Low risk	N/A	Low risk	N/A	Low risk	Unclear risk	Low risk
Soukoulis and Hirsch (2004)	Unclear risk	Unclear risk	N/A	Low risk	N/A	Unclear risk	Unclear risk	Unclear risk	Unclear risk
Srikumar et al. (2022)	Low risk	Low risk	Unclear risk	Unclear risk	Low risk	Low risk	Low risk	Low risk	High risk
Taalab et al. (2021)	Low risk	Low risk	Unclear risk	Unclear risk	Low risk	Low risk	Low risk	Low risk	Low risk

### 3.3 Dermatology

Trials conducted in the field of dermatology were published 1990 to 2022, and conducted in Australia (Bassett et al., 1990; Satchell et al., 2002a), Germany (Beikert et al., 2013; Rothenberger et al., 2016), Iran (Enshaieh et al., 2007; Beheshti Roy et al., 2014; Najafi-Taher et al., 2022), Brazil (Hugo Infante et al., 2023), or Korea (Cho and Choi, 2017), either within a hospital, an outpatient setting or a community setting.

#### 3.3.1 Acne vulgaris

Three trials tested the effect of tea tree oil-based gels containing 5%–6% tea tree oil on acne lesion counts and acne severity in subjects with mild to moderate acne vulgaris (Bassett et al., 1990; Enshaieh et al., 2007; Najafi-Taher et al., 2022). Bassett et al. (1990), compared a 5% tea tree oil gel to a 5% benzoyl peroxide lotion in 124 subjects aged 12–35 years. Both treatments significantly reduced the number of inflamed and non-inflamed acne lesions. The benzoyl peroxide lotion was more effective in reducing the number of inflamed lesions after 1, 2 and 3 months, although resulted in a greater degree of facial skin scaling and pruritus compared to the tea tree oil gel. Enshaieh et al. (2007), compared twice daily application of a 5% tea tree oil gel to a placebo carbomer gel for 45 days, in 60 subjects aged 15–25 years (Enshaieh et al., 2007). Reductions in both total lesion count and acne severity were significantly greater in subjects applying the tea tree oil gel compared with placebo (*p* < 0.001). Finally, Najai-Taher et al. (2022), compared once daily application of a 6% tea tree oil nano-emulsion gel with 0.1% adapalene to a commercial gel containing only 0.1% adapalene, over 12 weeks, in 100 subjects aged 15–40 years. Reductions observed in acne severity and in counts of inflammatory, non-inflammatory and total lesions were all significantly greater in subjects applying the tea tree oil + adapalene gel compared with adapalene only gel (*p* < 0.001).

Safety was measured in all three studies. Application of a 5% tea tree oil gel resulted in minimal pruritis (3/30), burning sensation (1/30), and minimal scaling (1/30), although similar numbers of subjects applying a placebo gel reported these events (Enshaieh et al., 2007). However, Basset et al. (1990), found subjects using a 5% tea tree oil gel reported less adverse effects (e.g., dryness, stinging and burning), than those using the benzoyl peroxide lotion (27/61 vs. 50/63, *p* < 0.001). Najafi-Taher et al. (2022) found a higher proportion of subjects using a 6% tea tree oil + adapalene gel reported irritation after 4 weeks use, compared with an adapalene only gel (43% vs.17%, *p* = 0.005), although, after 12 weeks, there was no difference between groups in irritation.

#### 3.3.2 Wound healing

Two trials tested tea tree oil for improving outcomes related to wound healing (i.e., blood flow, hemoglobin oxygenation and concentration, and skin surface temperature in the case of burns) (Rothenberger et al., 2016; Cho and Choi, 2017). Cho and Choi (2017), compared Burnshield^®^ foam wound dressings containing tea tree oil with Burn Cool Spray^®^ or running tap water, in 94 adult subjects (Cho and Choi, 2017). Subjects presenting to hospital emergency with a burn wound(s) within 3 hours of the incident were allocated one of the afore-mentioned treatments for 20 min. Running tap water was significantly more effective than Burnshield^®^ and Burn Cool Spray^®^ for reducing skin surface temperature. Safety was not assessed. Rothenberger et al. (2016), tested a saline solution with or without 5% tea tree oil on skin perfusion dynamics in 20 healthy subjects aged 23–38 years (Rothenberger et al., 2016). Blood flow was significantly higher in fingers immersed in the saline with tea tree oil solution, compared with saline alone. No difference was found in hemoglobin concentration or oxygenation. Subjects did not report any adverse reactions.

#### 3.3.3 Seborrheic dermatitis

Beheshti Roy et al. (2014), compared application of a 5% tea tree oil gel to a placebo (vehicle gel) three times daily to facial areas affected with seborrheic dermatitis in 54 subjects aged 18–45 years (Beheshti Roy et al., 2014). Clinical signs of erythema, scaling, itching, and greasy crusts were all significantly lower in subjects applying the tea tree oil gel compared with placebo gel after 4 weeks. No subject reported allergic irritation or inflammation.

#### 3.3.4 Skin inflammation

Beikert et al. (2013), tested the anti-inflammatory effect of a cooling ointment (100% *Unguentum leniens*) to which 5% tea tree oil was added, in 40 subjects aged 19–58 years (Beikert et al., 2013). One side of the subjects’ upper back was subject to UV-B irradiation to induce a local inflammatory response, while the other side was not. Using Fin Chambers^®^, the tea tree oil ointment was applied within one chamber and placed on the radiated skin and within another chamber placed on the non-radiated skin. In the other empty chambers, six different plant extracts, 1% hydrocortisone acetate, or 0.1% betamethasone valerate, each in the same vehicle ointment 100% *Unguentum leniens*, were applied, as well as the vehicle ointment alone. Local inflammation, measured by degree of skin erythema, did not significantly differ between the treatments 48 h post-irradiation. Skin areas where the tea tree oil ointment had been applied to non-irradiated skin showed increased skin erythema suggesting mild irritation. No serious side effects or contact dermatitis occurred in subjects during the trial.

#### 3.3.5 Skin photodamage

Hugo Infante et al. (2023), compared the effect of an oil-in-water emulsion (vehicle formula) to this same vehicle formula with either 2% pure tea tree oil or 2% tea tree oil in nano-emulsion form added, on skin photoaging (Hugo Infante et al., 2023). No significant changes were observed in hydration or sebum levels between groups. Compared with controls, the tea tree oil nano-emulsion resulted in improvements to the stratum granulosum, including a significant increase in keratinocyte area, while the pure tea tree oil significantly increased the average epidermis thickness and papillary depth. Further, collagen density and observed collagen networks significantly increased in both tea tree oil intervention groups, compared with the control vehicle formula (Table 1). Safety was not assessed (Hugo Infante et al., 2023).

#### 3.3.6 Dandruff

Satchell et al. (2002a), compared daily hair washing with a 5% tea tree oil shampoo or a placebo shampoo (i.e., same shampoo without tea tree oil) for treatment of dandruff in 126 subjects aged 16 and older (Satchell et al., 2002a). Reduction in both the area of scalp involved and severity of dandruff was significantly greater in subjects washing their hair with the tea tree oil shampoo compared with placebo. Adverse events reported by subjects using the tea tree oil shampoo (3/63) included mild stinging in eyes, mild burning of scalp, and mild itching of scalp. Those in the placebo group (8/62) reported pruritus, conjunctivitis, and urticaria.

#### 3.3.7 Risk of bias

Table 8 Selection bias due to use of a non-random or quasi-random allocation sequence was assessed as low risk in four of the nine trials conducted in the field of dermatology as a computer-generated sequence was used (Enshaieh et al., 2007; Cho and Choi, 2017; Hugo Infante et al., 2023; Najafi-Taher et al., 2022). The remainder provided insufficient information to assess this domain and were assessed as unclear risk. All nine dermatology trials were assessed as having an unclear risk of selection bias arising from inadequate concealment, as methods to conceal allocation sequences were not described. Primary outcomes in four of the nine trials were measured objectively (Beikert et al., 2013; Rothenberger et al., 2016; Cho and Choi, 2017; Hugo Infante et al., 2023). One of these trials was assessed as high risk, as personnel applying treatments also assessed the primary outcome and were not blinded to allocation (Cho and Choi, 2017). All four were assessed as low risk for detection bias as it was highly unlikely that the objective outcomes were affected by any lack of blinding and in one trial the assessor was blinded (Beheshti Roy et al., 2014). Five studies had subjectively assessed primary outcomes (Bassett et al., 1990; Satchell et al., 2002a; Enshaieh et al., 2007; Najafi-Taher et al., 2022). Three of these were assessed as high risk for performance bias as it is likely that blinding was broken through detection of tea tree oil odor (or lack thereof), and those receiving placebo may have altered their behavior as a result of unblinding (Satchell et al., 2002a; Enshaieh et al., 2007; Beheshti Roy et al., 2014). Intervention compliance was also not assessed in these studies. All subjective outcomes were assessed as low risk for detection bias. Attrition bias was mostly assessed as low risk, although one study was assessed as high risk given that twice the number of participants allocated to placebo dropped out and the reason provided (i.e., lost to follow-up “due to personal reasons”) is inadequate to eliminate a risk of bias (Beheshti Roy et al., 2014). Only one study had been registered on a public trial registry platform and was therefore assessed as low risk for reporting bias (Najafi-Taher et al., 2022), the remainder were assessed as unclear risk. Four trials were assessed as unclear risk for other bias as the source of tea tree oil used was not defined (Satchell et al., 2002a; Beikert et al., 2013; Rothenberger et al., 2016; Najafi-Taher et al., 2022).

**TABLE 8:** Risk of bias assessment of the included studies—Dermatology.

Bias author (year)	Random sequence generation (selection bias)	Allocation concealment (selection bias)	Blinding of participants and personnel (performance bias)		Blinding of outcome assessment (detection bias)		Incomplete outcome data (attrition bias)	Selective reporting (reporting bias)	Other bias
			Objective outcomes	Subjective outcomes	Objective outcomes	Subjective outcomes			
Bassett et al. (1990)	Unclear risk	Unclear risk	N/A	Unclear risk	N/A	Low risk	Low risk	Unclear risk	Low risk
Beheshti Roy et al. (2014)	Unclear risk	Unclear risk	N/A	High risk	N/A	Low risk	High risk	Unclear risk	Low risk
Beikert et al. (2013)	Unclear risk	Unclear risk	Low risk	N/A	Low risk	N/A	Low risk	Unclear risk	Unclear risk
Cho and Choi (2017)	Low risk	Unclear risk	High risk	N/A	Low risk	N/A	Low risk	Unclear risk	Low risk
Enshaieh et al. (2007)	Low risk	Unclear risk	N/A	High risk	N/A	Low risk	Unclear risk	Unclear risk	Low risk
Hugo Infante et al. (2023)	Low risk	Unclear risk	Low risk	N/A	Low risk	Unclear risk	Low risk	Unclear risk	Low risk
Najai-Taher et al. (2022)	Low risk	Unclear risk	N/A	Unclear risk	N/A	Low risk	Unclear risk	Low risk	Unclear risk
Rothenberger et al. (2016)	Unclear risk	Unclear risk	Low risk	N/A	Low risk	N/A	Low risk	Unclear risk	Unclear risk
Satchell et al. (2002a)	Unclear risk	Unclear risk	N/A	High risk	N/A	Low risk	Low risk	Unclear risk	Unclear risk

### 3.4 Infectious disease

Infectious diseases and/or infection control were addressed in nine trials published 2000 to 2021, conducted in Australia (Caelli et al., 2000), Brazil (Gnatta et al., 2013; Gnatta et al., 2021), Hong Kong (Lee et al., 2014), India (Maghu et al., 2016), Korea (Youn et al., 2021), Northern Ireland (Blackwood et al., 2013), United Kingdom (Dryden et al., 2004), or the United States (Markum and Baillie, 2012) within the hospital, outpatient, residential aged care, or community setting.

#### 3.4.1 Methicillin-resistant *Staphylococcus aureus* (MRSA) infection

Four trials tested the effect of topically applied tea tree oil-based products for the prevention of MRSA colonization (Blackwood et al., 2013), or eradication of MRSA bacteria from the body (Caelli et al., 2000; Dryden et al., 2004; Lee et al., 2014), in hospital care settings.

Blackwood et al. (2013), compared Novobac^®^ 5% tea tree oil body wash with standard care (i.e., Johnson’s Baby Softwash^®^) for preventing colonization with MRSA in 391 patients across two hospital intensive care units (Blackwood et al., 2013). Subjects received daily full bed baths with their allocated body wash until detection of ICU-acquired MRSA, discharge from ICU, or death. Compared with standard care, there was no reduction in the incidence of ICU-acquired MRSA infection (*p = 0.50*). A limitation to this study was that subjects using the Novobac^®^ 5% tea tree oil had a longer admission, longer duration of ventilation, greater number of days with devices in place, and a higher percentage of these patients were nursed adjacent to a patient infected with MRSA. However, in a multiple regression analysis including the strongest independent predictors of MRSA colonization, there remained no statistically significant difference between treatment group (tea tree oil or standard care) and incident MRSA colonization (Blackwood et al., 2013). Caelli et al. (2000), compared a standard care topical regimen for MRSA decolonization to a tea tree oil topical regimen in patients infected or colonized with MRSA (Caelli et al., 2000). MRSA decolonization was achieved in 5/15 patients who received the tea tree oil regimen and 2/15 patients who received standard care, after a minimum 3 day treatment. However, patients in the tea tree oil group had, on average, a longer length of stay, and thus, longer duration of treatment. In a similar, yet larger, study, Dryden et al. (2004), also compared a standard care regimen to a tea tree oil topical regimen (10% tea tree oil cream applied to nostrils, skin lesions, wounds, and ulcers as well as a 5% tea tree oil body wash) for MRSA decolonization (Dryden et al., 2004). Fourteen days post-treatment, a similar proportion of patients achieved MRSA decolonization (tea tree oil regimen 46/110 versus standard care regimen 56/114, *p* = 0.0286). The 10% tea tree oil cream which was applied to the nostrils three times daily, was however, found to be less effective at clearing MRSA from nasal passages when compared with Bactroban^®^ nasal ointment. Lastly, Lee et al. (2014), tested a topical tea tree oil solution (i.e., 10% tea tree oil with paraffin oil) for healing and decolonizing wounds infected with MRSA (Lee et al., 2014). All subjects received standard wound care, i.e., daily wound cleaning with 0.9% saline solution followed by application of a wound dressing for 4 weeks. Subjects allocated to receive the tea tree oil solution had this applied after cleaning the wound with the saline solution. CFU/ml of MRSA were found to be significantly lower in the wounds of patients who had the 10% tea tree oil solution applied to their wound at weeks 1, 2, 3 and week 4 (post-intervention). At these same timepoints, wound healing (based on wound size, exudate, and type of wound tissue) was significantly better with the tea tree oil solution compared with saline alone. Adverse events were measured in all four studies. No adverse events were reported by subjects receiving a tea tree oil topical regimen (10% tea tree oil cream and 5% tea tree oil body wash) (Dryden et al., 2004), or a topical 10% tea tree oil wound preparation (Lee et al., 2014). In the other two trials, using a tea tree oil body wash, no adverse events were reported in one trial (Caelli et al., 2000), while in the other, two subjects reported a rash which later were both found to be unrelated to the tea tree oil body wash used (Blackwood et al., 2013). However, subjects (numbers not stated) reported mild swelling of the nasal mucosa and acute burning upon application of the 4% tea tree oil nasal ointment (Caelli et al., 2000).

#### 3.4.2 Infection control (i.e., hand disinfection)

Three trials tested the effect of tea tree oil soaps (Gnatta et al., 2013; Gnatta et al., 2021), or a tea tree oil-based hand disinfectant (Youn et al., 2021) on microbial counts on the skin surface of hands. Youn et al. (2021), compared a tea tree oil-based hand disinfectant to a standard alcohol-based hand sanitizer, a benzalkonium chloride-based hand sanitizer and a no treatment control in 112 subjects aged 18–60 years (Youn et al., 2021). Skin surface log_10_ microbial count decreased in all groups, except in the no treatment control group. The greatest reduction was with use of the tea tree oil-based hand disinfectant, which achieved a significantly greater reduction compared with the alcohol-based hand sanitizer, the benzalkonium chloride-based hand sanitizer and no treatment (all *p* < 0.05). In two similar studies, Gnatta et al. (2013) and Gnatta et al. (2021), tested a tea tree oil hand soap for reducing the concentration of *Escherichia coli* K12 on the hand surface (Gnatta et al., 2013; Gnatta et al., 2021). Both studies enrolled 15 participants from a hospital workplace setting and used a cross-over design to compare a tea tree oil soap (0.2%–0.3% tea tree oil) to either Rioderm^®^, Soft Soap, or Soft Soap with 60% propan-2-ol hand rinse (in the 2013 study) (Gnatta et al., 2013), or to Rioderm^®^, Riohex^®^, and Soft Soap (in the 2021 study) (Gnatta et al., 2021). In the 2013, trial the tea tree oil soap (Doctornatu^®^ liquid soap), used to wash hands after *E. coli* K12 contamination, reduced the CFU/mL count *E. coli* K12 to a similar extent as Soft Soap and was inferior to Soft Soap with 60% propan-2-ol (*p* = 0.001). In the 2021 trial, the tea tree oil soap (2% tea tree oil) used significantly reduced *E. coli* K12 compared with chlorhexidine (*p* = 0.006), and with Soft Soap (*p* < 0.001), although no difference was found between tea tree oil soap and Rioderm^®^. Adverse events were not assessed in these three trials (Gnatta et al., 2013; Gnatta et al., 2021; Youn et al., 2021).

#### 3.4.3 Molluscum contagiosum

Markum and Baillie (2012), compared a topical formulation containing tea tree oil organically bound to iodine in a high selenium canola oil to the same vehicle oil with tea tree oil or with organically bound iodine (i.e., tea tree oil + iodine vs. tea tree oil alone vs. iodine alone), for the treatment of molluscum contagiosum lesions in 53 children (Markum and Baillie, 2012). A significantly greater number of children resolved > 90% of their lesions with the tea tree oil + iodine formulation (16/19), compared with tea tree oil alone (3/18) or iodine alone (1/16), after 30 days (both *p* < 0.01). Adverse events included redness at lesion site reported by one subject each in the tea tree oil + iodine and tea tree oil alone groups and by two subjects in the iodine alone group. A warm sensation on application was also reported in groups receiving tea tree oil i.e., tea tree oil + iodine (3/19) and tea tree oil (4/18).

#### 3.4.4 Oral candida infection

Maghu et al. (2016), compared a 0.25% tea tree oil mouthwash to an anti-fungal (clotrimazole) topical ointment or a standard care regime for the treatment of oral Candida infection in 36 subjects aged 20–60 years (Maghu et al., 2016). Clinician- assessed signs of erythema, inflammation, and fungal hyphae (*highly diagnostic of infection with Candida*), decreased in all groups, although larger reductions were seen in the tea tree oil mouthwash and clotrimazole ointment groups, compared with standard care (*note: no statistical comparisons were performed*). Adverse effects were not reported by any subjects using the tea tree oil mouthwash.

#### 3.4.5 Risk of bias

All domains were mostly assessed as low or unclear risk of bias (Table 9). Two trials were assessed as high risk for selection bias, due to the use of an allocation sequence that could have been predicted by those allocating interventions (Gnatta et al., 2013; Gnatta et al., 2021). High risk of detection bias was also found in one trial using subjectively assessed outcomes, as any attempt at blinding outcome assessors was likely broken due to the different treatment modalities used (Maghu et al., 2016). Only three trials had published their trial protocol on a public register (Markum and Baillie, 2012; Blackwood et al., 2013; Youn et al., 2021), and were thus assessed as having low risk of reporting bias. Three trials were assessed as having unclear risk of performance bias, as it was not reported whether personnel applying tea tree oil products were blinded and, this may have influenced doses used on subjects (Caelli et al., 2000; Dryden et al., 2004; Lee et al., 2014). Finally, six of the nine infectious disease studies were assessed as having an unclear risk of selection bias due to insufficient information on how a random sequence was generated (Caelli et al., 2000; Markum and Baillie, 2012; Gnatta et al., 2013; Lee et al., 2014; Maghu et al., 2016; Gnatta et al., 2021).

**TABLE 9:** Risk of bias assessment of the included studies—Infectious Disease.

Bias author (year)	Random sequence generation (selection bias)	Allocation concealment (selection bias)	Blinding of participants and personnel (performance bias)		Blinding of outcome assessment (detection bias)		Incomplete outcome data (attrition bias)	Selective reporting (reporting bias)	Other bias
			Objective outcomes	Subjective outcomes	Objective outcomes	Subjective outcomes			
Blackwood et al. (2013)	Low risk	Low risk	Low risk	N/A	Low risk	N/A	Low risk	Low risk	Low risk
Caelli et al. (2000)	Unclear risk	Unclear risk	Unclear risk	N/A	Low risk	N/A	Unclear risk	Unclear risk	Low risk
Dryden et al. (2004)	Low risk	Unclear risk	Unclear risk	N/A	Low risk	N/A	Low risk	Unclear risk	Low risk
Gnatta et al. (2021)	Unclear risk	High risk	Low risk	N/A	Low risk	N/A	Low risk	Unclear risk	Low risk
Gnatta et al. (2013)	Unclear risk	High risk	Low risk	N/A	Low risk	N/A	Low risk	Unclear risk	Low risk
Lee et al. (2014)	Unclear risk	Unclear risk	Unclear risk	Unclear risk	Low risk	Unclear risk	Unclear risk	Unclear risk	Low risk
Maghu et al. (2016)	Unclear risk	Unclear risk	N/A	Unclear risk	N/A	High risk	Unclear risk	Unclear risk	High risk
Markum and Baillie (2012)	Unclear risk	Unclear risk	N/A	Low risk	N/A	Low risk	Low risk	Low risk	Low risk
Youn et al. (2021)	Low risk	Unclear risk	Low risk	N/A	Low risk	N/A	Low risk	Low risk	Low risk

### 3.5 Ophthalmology

Six trials were published in the field of ophthalmology from 2012 to 2021, and were conducted in Australia (Wong et al., 2019), Canada (Craig et al., 2022), Iran (Mohammadpour et al., 2020; Zarei-Ghanavati et al., 2021), Korea (Koo et al., 2012), or Turkey (Karakurt and Zeytun, 2018), within the hospital or outpatient clinic setting.

#### 3.5.1 Demodex infestation

Three studies tested tea tree oil-based eyelid wipes, washes or scrubs for control of *Demodex* mites in subjects with diagnosed demodectic blepharitis (Karakurt and Zeytun, 2018), ocular surface discomfort (Koo et al., 2012), or in generally healthy adults (Wong et al., 2019).

Karakurt and Zeytun (2018), compared twice daily eyelid washes with Blefaroshampoo^®^ (with 7.5% tea tree oil) with Blepharitis Shampoo (without tea tree oil) in 135 adult subjects (Karakurt and Zeytun, 2018). Koo et al. (2012), compared twice daily eyelid scrubs with 10% tea tree oil diluted in mineral oil to a saline eyelid scrub in 281 adult subjects (Koo et al., 2012). In addition to these twice daily eyelid scrubs performed at home, weekly eyelid scrubs were also performed within a clinic with 50% tea tree oil diluted in mineral oil (used in tea tree oil group) or with saline (for saline control group). Wong et al. (2019), compared BlephaDex™ eyelid wipes (containing tea tree oil) to a no treatment control in only 20 subjects aged 45 years and older (Wong et al., 2019). Subjects were randomly allocated BlephaDex™ eyelid wipes to use on their right or left eye in this within-group trial, while the contralateral eye received no treatment. In all three trials, interventions were 1 month in duration and all measured *Demodex* mite count at baseline and after 1 month. In two trials, use of tea tree oil significantly reduced *Demodex* mite count to a greater extent than saline or no treatment (Koo et al., 2012; Wong et al., 2019). Karakurt and Zeytun (2018), also found a larger reduction in Demodex mite count in those using Blefaroshampoo (with tea tree oil), compared with those using Blepharitis Shampoo (without tea tree oil), although no between group comparison was performed. Two of these trials measured patient-assessed ocular symptoms at baseline and after 1 month (Koo et al., 2012; Karakurt and Zeytun, 2018). Koo et al. (2012) found a greater reduction in patient-reported ocular discomfort after 1 month with twice daily eyelid scrubs with 10% tea tree oil diluted in mineral oil, compared with saline eyelid scrubs, but only among subjects who performed tea tree oil eyelid scrubs at home > 10 times per week (i.e., had good compliance with the intervention). Karakurt and Zeytun (2018) found patient-reported ocular symptoms including itching, burning, feeling of a foreign body in the eye, eye redness, and cylindrical dandruff all significantly decreased with use of the Blefaroshampoo (with tea tree oil), while those subjects using the Blepharitis Shampoo (without tea tree oil) had similar symptom scores after 4 weeks.

To provide data on the tolerability of different tea tree oil-based eyelid wipe products, Craig et al. (2022), compared four commercial tea tree oil-based eyelid wipes (i.e., Oust™ Demodex^®^, I-Lid’n Lash^®^ Plus, Blephadex™ and Eye Cleanse™) to a saline solution (i.e., Sensitive Eyes^®^ Plus Saline Solution), in terms of tear film break up time, as well as ocular discomfort and redness (Craig et al., 2022). Ten-minutes after application, Eye Cleanse™ lid cleansing wipes was the only product to significantly decrease tear film break up time. Eye Cleanse™ wipes also increased ocular redness (both bulbar and limbal) and corneal and conjunctival staining—a measure of damage to ocular surfaces. I-Lid’n Lash^®^ Plus also caused a significant, yet smaller, increase in bulbar ocular redness. Patient-assessed ocular discomfort remained significantly higher than baseline for a median 195 s after applying Eye Cleanse™, 150 s after I-Lid’n Lash^®^ Plus, 120 s after Oust™, and 60 s after Blephadex™ (Craig et al., 2022). Median time after which participants could comfortably open their eyes was one second for the saline solution, compared with 3 s for Oust™, 5 s for Blephadex™, 6 s for I-Lid’n Lash^®^ Plus (*p* < 0.05), and 30 s for EyeCleanse™ (*p* < 0.001) (Craig et al., 2022). Only EyeCleanse™ significantly increased lid wiper epitheliopathy, although all cases were resolved within 24 h and no other adverse events were recorded.

#### 3.5.2 Dry eye post cataract surgery

Mohammadpour et al. (2020), compared a tea tree oil-based eyelid shampoo (Eyesol^®^, with 5% tea tree oil), with and without an additional 5% tea tree oil added, for treating dry eye post phacoemulsification cataract surgery (Mohammadpour et al., 2020). After 1 month, subjects Eyesol^®^ with 5% additional TTO shampoo had significantly lower *Demodex* counts, improved tear osmolarity and break-up time and less ocular discomfort, compared with Eyesol^®^ shampoo alone. Safety not assessed.

#### 3.5.3 Meibomian gland dysfunction

Zarei-Ghanavati et al. (2021), also tested the tea tree oil-based eyelid shampoo Eyesol^®^ and compared this to Johnson’s baby shampoo for treating meibomian gland dysfunction (Zarei-Ghanavati et al., 2021). In this within-patient RCT, Eyesol^®^ was randomly allocated to either the right or left eye, with Johnson’s baby shampoo used on the contralateral eye. Compared with Johnson’s baby shampoo, improvement in patient-assessed severity of dry eye was significantly greater in those using Eyesol^®^ and clinician-assessed signs of meibomian gland expressibility, plugging, capping, foamy tear, telangiectasia and tear break-up time also significantly improved with Eyesol^®^, after 3 months of daily eyelid washing. All other outcomes assessed showed no significant difference between groups (Table 4).

Adverse events were measured in three of the five ophthalmology trials (Koo et al., 2012; Wong et al., 2019; Zarei-Ghanavati et al., 2021). Ocular irritation was the main side effect of tea tree oil use reported by 5/106 subjects using a tea tree oil-based mineral oil solution (Koo et al., 2012) and 21/40 subjects using tea tree oil-based Eyesol^®^ shampoo (compared with 12/40 reporting this symptom from use of baby shampoo) (Zarei-Ghanavati et al., 2021). Further, 1/20 subjects using Blephadex™ eyelid wipes reported slight discomfort upon initial use which later resolved (Wong et al., 2019).

#### 3.5.4 Risk of bias

All domains were mostly assessed as low or unclear risk of bias (Table 10). Two trials measuring patient-reported ocular symptoms were assessed as high risk for detection bias as any attempt at blinding was likely broken due to the distinct odor of tea tree oil in the eyelid products used (Koo et al., 2012; Karakurt and Zeytun, 2018). The study by Koo et al. (2012) was also assessed as having high risk for attrition bias with 43.1% (160/281) attrition after only 1 month. Attrition was also twice as high in the tea tree oil intervention group with no reasons for drop-out reported.

**TABLE 10:** Risk of bias assessment of the included studies—Ophthalmology.

Bias author (year)	Random sequence generation (selection bias)	Allocation concealment (selection bias)	Blinding of participants and personnel (performance bias)		Blinding of outcome assessment (detection bias)		Incomplete outcome data (attrition bias)	Selective reporting (reporting bias)	Other bias
			Objective outcomes	Subjective outcomes	Objective outcomes	Subjective outcomes			
Craig et al. (2022)	Low risk	Low risk	Low risk	Low risk	Low risk	Low risk	Low risk	Unclear risk	Low risk
Karakurt and Zeytun (2018)	Unclear risk	Unclear risk	Low risk	Low risk	Low risk	High risk	Low risk	Unclear risk	Unclear risk
Koo et al. (2012)	Unclear risk	Unclear risk	Low risk	Low risk	Low risk	High risk	High risk	Unclear risk	Low risk
Mohammadpour et al. (2020)	Low risk	Low risk	Low risk	Low risk	Low risk	Unclear risk	Unclear risk	Low risk	Low risk
Wong et al. (2019)	Low risk	Unclear risk	Unclear risk	N/A	Low risk	N/A	Low risk	Low risk	Low risk
Zarei-Ghanavati et al. (2021)	Low risk	Low risk	Unclear risk	Unclear risk	Low risk	Unclear risk	Unclear risk	Low risk	Low risk

### 3.6 Podiatry

Three trials were published in the field of podiatry from 1992 to 2002, and conducted in Australia (Tong et al., 1992; Satchell et al., 2002b) or the United States (Buck et al., 1994), within hospital or community outpatient clinics.

#### 3.6.1 Tinea pedis

Both Australian trials tested tea tree oil for the treatment of tinea pedis (Athlete’s foot), compared with a placebo (i.e., vehicle solution) (Tong et al., 1992; Satchell et al., 2002b), or with Tinaderm^®^ (Tong et al., 1992). Satchell et al. (2002b), compared a solution containing tea tree oil (either 50% or 25% concentration) to a placebo solution (vehicle solution), applied topically for 4 weeks in 137 subjects aged 17–83 years (Satchell et al., 2002b). After 4 weeks, a higher proportion of subjects treated with either the 50% or 25% tea tree oil solution had a negative mycological culture, compared with subjects receiving the vehicle solution (23/36 or 18/33 versus 14/45, *p* < 0.01) (Satchell et al., 2002b). In a separate trial, Tong et al. (1992), compared a sorbolene cream with 10% tea tree oil to a placebo vehicle cream (i.e., 100% sorbolene cream) applied topically for 4 weeks in 121 subjects aged 16–65 years. After 4 weeks, there was no significant difference in the number of patients achieving a negative mycological culture (*p* = 0.393) (Tong et al., 1992). When compared to Tinaderm^®^, a higher proportion of subjects applying Tinaderm^®^ had a negative mycological culture, compared with tea tree oil (*p* < 0.001). In both trials, clinical signs, and symptoms of tinea pedis (i.e., scaling, inflammation, burning and itching) improved in a greater proportion of subjects using tea tree oil, compared with placebo (Tong et al., 1992; Satchell et al., 2002b). Adverse events were assessed in both trials (Tong et al., 1992; Satchell et al., 2002b). Three of the 69 subjects treated with a 50% tea tree oil solution and one treated with a 25% tea tree oil solution developed moderate to severe dermatitis which improved upon ceasing treatment (Satchell et al., 2002b). One subject applying Tinaderm^®^ developed mild erythema (Tong et al., 1992).

#### 3.6.2 Onychomycosis

Buck et al. (1994), tested twice daily topical application of 100% tea tree oil, compared with a 1% clotrimazole antifungal solution, for 6 months for the treatment of onychomycosis—a fungal toenail infection (Buck et al., 1994). Tea tree oil was found to be as effective as the clotrimazole solution based on negative fungal culture at 6 months (i.e., post-intervention). The toenail assessed as having the greatest fungal involvement at baseline showed “full” or “partial” resolution in a similar proportion of patients in tea tree oil and clotrimazole groups. Further, after 9 months (i.e., 3 months post-intervention), a similar proportion of patients reported that their toenail appearance and symptoms had either improved or completely resolved. Adverse events (i.e., erythema, irritation, and edema) were experienced by 5/64 using 100% tea tree oil compared with 3/53 using the clotrimazole solution.

#### 3.6.3 Risk of bias

All domains were mostly assessed as low or unclear risk of bias (Table 11). Unclear risk was assigned for absence of information on methods for randomizing participants to intervention groups, and the lack of publicly available trial protocols either in a published manuscript or on a trial registry platform. Two trials were assessed as high risk for detection bias for patient-reported symptoms, given the likely breaking of blinding due to the distinct odor of tea tree oil (Tong et al., 1992; Buck et al., 1994).

**TABLE 11:** Risk of bias assessment of the included studies—Podiatry.

Bias author (year)	Random sequence generation (selection bias)	Allocation concealment (selection bias)	Blinding of participants and personnel (performance bias)		Blinding of outcome assessment (detection bias)		Incomplete outcome data (attrition bias)	Selective reporting (reporting bias)	Other bias
			Objective outcomes	Subjective outcomes	Objective outcomes	Subjective outcomes			
Buck et al. (1994)	Low risk	Low risk	Low risk	Low risk	Low risk	High risk	Low risk	Unclear risk	Low risk
Satchell et al. (2002b)	Unclear risk	Unclear risk	Unclear risk	N/A	Low risk	N/A	Low risk	Unclear risk	Unclear risk
Tong et al. (1992)	Unclear risk	Unclear risk	Low risk	Low risk	Low risk	High risk	Unclear risk	Unclear risk	Unclear risk

### 3.7 Other fields

#### 3.7.1 Anxiety and sleep quality

Ozkaraman et al. (2018), tested the effect of Lavender essential oil, in comparison to tea tree oil or no treatment, on anxiety and sleep quality in 70 patients undergoing weekly chemotherapy (Ozkaraman et al., 2018). Three drops of a commercially available tea tree oil (source not defined) were placed on a piece of cotton and positioned on the patient’s neck during their chemotherapy session. Patients were also instructed to smell their allocated oil at home every evening at 9 p.m. for 5 min for 1 month. Lavender essential oil significantly decreased trait anxiety (e.g., being a steady person, not worrying too much over something that doesn’t matter), while no change was observed with tea tree oil. No difference was found between groups in the change in state anxiety scores (e.g., being tense or worried). Tea tree oil was found to be as effective as lavender essential oil in improving sleep quality, although baseline scores for sleep quality were higher in the tea tree oil group (Ozkaraman et al., 2018). Safety was not assessed.

#### 3.7.2 Risk of bias

Table 12 Given the distinct smell of lavender and tea tree and the use of a no treatment control, it would not have been possible to blind participants to treatment allocation (Ozkaraman et al., 2018). Thus, performance and detection bias were assessed as high risk for the two primary patient-reported outcomes assessed: State-Trait Anxiety Inventory and Pittsburgh Sleep Quality Index. Attrition bias and reporting bias were both assessed as unclear risk as there was no information on attrition and the trial protocol was not made publicly available. Finally, other bias was assessed as unclear risk given the tea tree oil used was not described.

**TABLE 12:** Risk of bias assessment of the included studies—Other.

Bias author (year)	Random sequence generation (selection bias)	Allocation concealment (selection bias)	Blinding of participants and personnel (performance bias)		Blinding of outcome assessment (detection bias)		Incomplete outcome data (attrition bias)	Selective reporting (reporting bias)	Other bias
			Objective outcomes	Subjective outcomes	Objective outcomes	Subjective outcomes			
Ozkaraman et al. (2018)	Low risk	Low risk	N/A	High risk	N/A	High risk	Unclear risk	Unclear risk	Unclear risk

## 4 Discussion

### 4.1 Summary of key findings

This review aimed to critically appraise evidence from randomized control trials examining the therapeutic efficacy and safety of tea tree oil on outcomes related to human health. The search yielded 46 eligible studies spanning the fields of dentistry, dermatology, infectious disease, ophthalmology, podiatry, as well as anxiety and sleep quality—a substantial expansion in research since the 2000 review by Ernst & Huntley which identified only four trials (Ernst and Huntley, 2000).

#### 4.1.1 Dentistry

Mouthwashes containing 0.2%–0.5% tea tree oil may be more effective in reducing dental plaque accumulation than a placebo mouthwash (Kamath et al., 2020; Reddy et al., 2020), or a 0.1% chlorine dioxide mouthwash (Bharadwaj et al., 2020), but not in comparison to a 0.12% chlorhexidine mouthwash (Casarin et al., 2019; Kamath et al., 2020). Evidence on the effects of tea tree oil mouthwashes on gingival inflammation and gingival bleeding is conflicting, and further studies are needed to confirm observed antimicrobial effects of tea tree oil mouthwashes on salivary *S. mutans* (Prabhakar et al., 2009; Kamath et al., 2020). Side effects of tea tree oil mouthwashes were minor with burning sensation most frequently reported, although this was also reported with other mouthwashes tested. Adjunctive therapy with a 5% tea tree oil gel delivered locally to the periodontium may improve SRP treatment outcomes in patients with periodontitis (Elgendy et al., 2013; Raut and Sethi, 2016; Taalab et al., 2021). We found insufficient evidence for the use of tea tree oil in treating *C. albicans* induced denture stomatitis, oral *C. albicans* infection, or in prevention of patient-clinician cross-contamination (Catalán et al., 2008; Shetty et al., 2013; Maghu et al., 2016).

#### 4.1.2 Dermatology

For the treatment of acne, a 5% tea tree oil-based topical gel may be effective in reducing the number of lesions and severity of acne, in comparison to a placebo (Enshaieh et al., 2007). However, tea tree oil was less effective for reducing the number of inflamed lesions compared with topical benzoyl peroxide (i.e., standard care) (Bassett et al., 1990). A combination of a tea tree oil nano-emulsion with adapalene in a topical gel shows potential as a novel treatment option for reducing lesion counts and severity of acne (Najafi-Taher et al., 2022). Clinical signs and symptoms of seborrheic dermatitis might be improved with a 5% tea tree oil topical gel, although findings are based on one small poor-quality trial (Beheshti Roy et al., 2014). Available evidence does not support the use of a tea tree oil dressing for treating burn wounds, with running tap water being more effective in cooling burns (Cho and Choi, 2017). Topical application of a 5% tea tree oil ointment was also found to be ineffective for reducing skin erythema in a UVB radiation model (Beikert et al., 2013). Topical application of tea tree oil in the concentrations used in these trials can result in mild reactions, such as burning sensation or itching upon application.

#### 4.1.3 Infectious diseases

While daily bed baths with a 5% tea tree oil body wash did not prevent patient colonization with MRSA (Blackwood et al., 2013), a topical tea tree oil-based regime showed similar efficacy to a routine topical regimen for MRSA decolonization in patients already colonized with MRSA bacteria (Caelli et al., 2000; Dryden et al., 2004). One smaller trial found 10% tea tree oil in paraffin oil applied to wounds colonized with MRSA improved wound healing and reduced MRSA bacterial counts (Lee et al., 2014). Adverse effects in MRSA trials were limited to mild swelling of the nasal mucosa and acute burning upon application of a 4% tea tree oil nasal ointment. Treating molluscum contagiosum lesions in young children may also be possible with topical application of 75% tea tree oil with iodine, with adverse events limited to mild redness at lesion site and warm sensation on application (Markum and Baillie, 2012).

#### 4.1.4 Ophthalmology

Tea tree oil-based eyelid wipes or scrubs aid reduction of ocular *Demodex* mite counts when compared with saline or no treatment (Koo et al., 2012; Wong et al., 2019). This finding is consistent with the review by Lam et al. (2020), who reported tea tree oil showed efficacy in controlling *Demodex* mite populations. Use of wipes may be safer, as ocular irritation can occur with tea tree oil eyelid scrubs (Koo et al., 2012). There is substantial difference however in the tolerability of commercially available eyelid cleansers (Craig et al., 2022). Further, more evidence is required to confirm efficacy and safety of tea tree oil-based eyelid shampoos for treating dry eye and meibomian gland dysfunction (Mohammadpour et al., 2020; Zarei-Ghanavati et al., 2021).

#### 4.1.5 Podiatry

Dermatophyte fungi causing tinea pedis may effectively be treated with tea tree oil applied topically in either 25% or 50% concentration, although moderate to severe dermatitis may present as a side effect and efficacy of this treatment has not been compared with standard care (Satchell et al., 2002b).

#### 4.1.6 Other

Finally, there was insufficient evidence to advise use of tea tree oil for improving anxiety or quality of sleep, with lavender essential oil being potentially more efficacious for reducing anxiety, at least during chemotherapy (Ozkaraman et al., 2018).

### 4.2 Alignment with existing systematic reviews

Several systematic reviews on tea tree oil have been conducted previously. About 20 years ago, Ernst and Huntley identified only four randomized controlled trials addressing acne, tinea pedis or onychomycosis (Ernst and Huntley, 2000). Since then, a further three systematic reviews have been published, although all focus on specific therapeutic indications only (Casarin et al., 2018; Lam et al., 2020; Savla et al., 2020). Savla et al. (2020), included six RCTs examining efficacy of tea tree oil for *Demodex* blepharitis with a total of 562 participants (Savla et al., 2020). While they found lower number of mites compared to the control interventions, they concluded that there was uncertainty whether 5%–50% tea tree oil was effective for treating *Demodex* blepharitis (Savla et al., 2020). With the lack of long-term studies, they also concluded that lower concentrations may be preferred to reduce potential side effects such as skin irritation. Lam et al. (2020), compared current pharmaceutical treatments for human demodicosis, and examined 95 studies, including trials on tea tree oil (Lam et al., 2020). They concluded that tea tree oil was the most effective, followed by metronidazole, ivermectin and permethrin, and the authors recommended tea tree oil as a first-line therapy. In our opinion, this recommendation is not supported by existing evidence. While the results for treating *Demodex* are promising, the lack of high-quality evidence, and the concerns around safety, especially with higher concentrations of tea tree oil, need to be considered, and further research is needed before such recommendations can be made. Casarin et al. (2018), included 25 studies addressing periodontal disease, however only six of those were clinical trials, whereas the present systematic review found 15 RCTs addressing periodontal disease prevention and treatment (Casarin et al., 2018). Casarin et al., 2018, suggested that tea tree oil has potential anti-inflammatory and antimicrobial properties, although we found that existing evidence for anti-inflammatory outcomes (i.e., gingival inflammation) is conflicting, and further trials are needed to confirm antimicrobial effects.

### 4.3 Limitations

This review has several limitations, the most important concerning the source and quality of tea tree oils. Only 24 of the 43 included studies stated the brand of tea tree oil or tea tree oil product that was used for the intervention. The remainder only stated that *M. alternifolia* was used or, in four trials (Soukoulis and Hirsch, 2004; Maghu et al., 2016; Karakurt and Zeytun, 2018; Ozkaraman et al., 2018), it was only stated that “tea tree oil” was used. Few stated the chemical composition of the tea tree oil used or the storage conditions. While tea tree oil is stable for more than 12 months under optimal conditions, exposure to oxygen, heat and light can cause rapid degradation of tea tree oil (AgriFutures Australia, 2021). Oxidized tea tree oil leads to a decrease in α-terpinene and γ-terpinene, and an increase in p-cymene and peroxide, which are main drivers of sensitization, and skin irritation (Hammer et al., 2006). Further to this, a recent study identified that nearly 50% of commercial tea tree oil samples showed signs of adulteration (Bejar, 2017). Adulterated tea tree oil is prepared by blending fractions of cheaper oils as well as adding synthetic analogues of terpinen-4-ol, and without detailed information about the product, the brand, and the composition of the tea tree oil product investigated, results of the trials included in this review cannot be attributed to *M. alternifolia* essential oil with certainty. Adulterated tea tree oil may also cause adverse reactions that would otherwise not occur with pure tea tree oil essential oil, leading to potentially erroneous safety profiles.

A general limitation to clinical research investigating tea tree oil is the difficulty in blinding of participants and personnel due to the strong unique aroma of tea tree oil. It is well understood that tea tree oil has a strong odor and taste, which can easily be distinguished by trial participants. While many trials claimed that participants were blinded, it is unclear whether the blinding was successful, and no data were collected to ascertain blinding in most studies. Researchers have previously been able to successfully blind participants by use of deception, i.e., falsely advising patients that the aroma of their treatment had been changed to prevent detection of tea tree oil (Carson et al., 2008). In addition, most trials did not assess compliance/adherence with the trial interventions, or, adverse events, including in very recent trials, highlighting the importance of comprehensive evaluation of efficacy and safety outcomes. Concerningly, some trials did not state the doses or frequency at which the tea tree oil interventions were used.

Another limitation to consolidating evidence from trials investigating tea tree oil is the heterogeneity of tea tree oil-based product formulations. Variations in formulations can result in huge differences in product efficacy, for example a sorbolene base for tea tree oil used for acne was shown to inhibit its efficacy (personal communication), and acne products may require an alternative carrier. In the last decade, many novel formulations have been developed for drug delivery, and it is essential to understand the limitations and benefits of delivery systems to design appropriate tea tree oil products with improved effectiveness and safety, adequate stability, and shelf-life. As such, it is recommended that all new tea tree oil formulations are rigorously tested *in-vitro*, *in-vivo*, and in clinical trials prior to release onto the market. Finally, researchers must adhere to standard research design and reporting guidelines, as well as register their trials prospectively in national/international trial databases for transparency.

### 4.4 Safety issues

The question of the safety of tea tree oil has been a controversial topic for decades. Issues have been raised around its ability to cause dermal irritation and its potential as an allergic sensitizer. Several studies have been conducted, and while tea tree oil was ranked twenty-third on the list of most frequent allergens reported in Australia (Toholka et al., 2015), studies in North America and Europe found relatively low allergenicity rates to tea tree oil (de Groot and Schmidt, 2016). It is however accepted, that most allergic reactions occur with aged/oxidized tea tree oil due to the degradation of its components. In addition, case reports have linked tea tree oil with gynecomastia in young boys (Henley et al., 2007), although recent studies refuted these findings and found that it was implausible for tea tree oil to cause endocrine disruptions (Hawkins et al., 2020; Hawkins et al., 2021). Lastly, a recent report by Lee et al. (2020), found that 17% of adverse events due to exposure to essential oils were caused by tea tree oil (Lee et al., 2020). According to US data from the National Electronic Injury Surveillance System (NEISS) from 2010 to 2020 (https://www.cpsc.gov/Research--Statistics/NEISS-Injury-Data), most injuries associated with tea tree oil were due to ingestion in toddlers and children, with an overall low hospitalization rate, and no deaths. The large number of incidents (nearly 350 in 2017), however, warrants better consumer protection, such as mandatory child-proof safety caps on tea tree oil bottles.

## 5 Conclusion

This systematic review provides the first comprehensive appraisal of human trials testing the therapeutic efficacy and safety of tea tree oil. Therapeutic indications for which tea tree oil has been tested span multiple fields of medicine including dentistry, dermatology, infectious disease, ophthalmology, and podiatry.

In contrast to the well-established traditional uses of tea tree plant, no trials were identified for the treatment of minor wounds, furuncles, or insect bites of tea tree oil. Such analgesic and antipruritic properties of tea tree oil are not well documented, and further research into the modes of action are warranted. One recent trial was located on the treatment of vaginitis, suggesting tea tree oil pessaries were an effective treatment for vaginitis, although this trial was excluded on the basis of study design (Durić et al., 2021). While tea tree leaves have traditionally been used for treating coughs and colds, no clinical trials testing tea tree oil for these indications were found, and the safety of tea tree oil for inhalation is poorly understood.

Reliability of the evidence from many of these trials suffered from inadequate reporting, particularly reporting of the source and quality of tea tree oils used on patients. Future investment in rigorous human clinical trials is warranted to clarify potential benefits of tea tree oil for treatment of acne, molluscum contagiosum lesions, microbial causes of tooth decay, periodontal disease, and oral fungal conditions, infections caused by antibiotic-resistant bacteria, and ocular conditions caused by *Demodex* mite infestation. In future trials, emphasis should focus on comparing tea tree oil to current standard care as well as an appropriate placebo. Novel formulations should also be considered to enhance localized delivery of active components of tea tree oil. Further, it is critical to assess safety of tea tree oil treatments to better understand the risks to patients and consumers of these products.

## Data Availability

The original contributions presented in the study are included in the article/Supplementary Material, further inquiries can be directed to the corresponding author.

## References

[B1] AgriFutures Australia (2021). Updated report on the efficacy and safety of tea tree oil.

[B2] Bassett I. B. Pannowitz D. L. Barnetson R. S. (1990). A comparative study of tea-tree oil versus benzoylperoxide in the treatment of acne. Med. J. Aust. 153 (8), 455–458. 10.5694/j.1326-5377.1990.tb126150.x 2145499

[B3] Beheshti Roy A. Tavakoli-Far B. Fallah Huseini H. Tousi P. Shafigh N. Rahimzadeh M. (2014). Efficacy of melaleuca alternifolia essential oil in the treatment of facial seborrheic dermatitis: A double-blind, randomized, placebo-controlled clinical trial. J. Med. Plants 13 (51), 26–32.

[B4] Beikert F. C. Schonfeld B. S. Frank U. Augustin M. (2013). Antiinflammatory potential of seven plant extracts in the ultraviolet erythema test. A randomized, placebo-controlled study. Hautarzt 64 (1), 40–46. 10.1007/s00105-012-2505-x 23337964

[B5] Bejar E. (2017). Adulteration of tea tree oil (Melaleuca alternifolia and M. linariifolia). Botanical Adulterants Prevention Bulletin, 1–8.

[B6] Bharadwaj A. N. Byatappa V. Raju R. Umakanth R. Alayadan P. (2020). Antiplaque and antigingivitis effectiveness of tea tree oil and chlorine dioxide mouthwashes among young adults: A randomized controlled trial. World J. Dent. 11 (6), 451–456. 10.5005/jp-journals-10015-1765

[B7] Blackwood B. Thompson G. McMullan R. Stevenson M. Riley T. V. Alderdice F. A. (2013). Tea tree oil (5%) body wash versus standard care (Johnson's Baby Softwash) to prevent colonization with methicillin-resistant *Staphylococcus aureus* in critically ill adults: a randomized controlled trial. J. Antimicrob. Chemother. 68 (5), 1193–1199. 10.1093/jac/dks501 23297395

[B8] Braun L. Cohen M. Monographs A-Z. (2005). in Herbs & natural supplements: An evidence-based guide (Sydney: Elsevier Mosby).

[B9] Buck D. S. Nidorf D. M. Addino J. G. (1994). Comparison of two topical preparations for the treatment of onychomycosis: Melaleuca alternifolia (tea tree) oil and clotrimazole. J. Fam. Pract. 38 (6), 601–605. 8195735

[B10] Caelli M. Porteous J. Carson C. F. Heller R. Riley T. V. (2000). Tea tree oil as an alternative topical decolonization agent for methicillin-resistant *Staphylococcus aureus* . J. Hosp. Infect. 46 (3), 236–237. 10.1053/jhin.2000.0830 11073734

[B11] Carson C. F. Hammer K. A. Riley T. V. (2006). Melaleuca alternifolia (tea tree) oil: A review of antimicrobial and other medicinal properties. Clin. Microbiol. Rev. 19 (1), 50–62. 10.1128/CMR.19.1.50-62.2006 16418522 PMC1360273

[B12] Carson C. F. Smith D. W. Lampacher G. J. Riley T. V. (2008). Use of deception to achieve double-blinding in a clinical trial of Melaleuca alternifolia (tea tree) oil for the treatment of recurrent herpes labialis. Contemp. Clin. Trials 29 (1), 9–12. 10.1016/j.cct.2007.04.006 17544340

[B13] Casarin M. Pazinatto J. Oliveira L. M. Souza M. E. Santos R. C. V. Zanatta F. B. (2019). Anti-biofilm and anti-inflammatory effect of a herbal nanoparticle mouthwash: a randomized crossover trial. Braz Oral Res. 33, e062. 10.1590/1807-3107bor-2019.vol33.0062 31859706

[B14] Casarin M. Pazinatto J. Santos R. C. V. Zanatta F. B. (2018). Melaleuca alternifolia and its application against dental plaque and periodontal diseases: A systematic review. Phytother. Res. 32 (2), 230–242. 10.1002/ptr.5974 29235165

[B15] Catalán A. Pacheco J. G. Martínez A. Mondaca M. A. (2008). *In vitro* and *in vivo* activity of melaleuca alternifolia mixed with tissue conditioner on Candida albicans. Oral Surg. Oral Med. Oral Pathology, Oral Radiology Endodontology 105 (3), 327–332. 10.1016/j.tripleo.2007.08.025 18280967

[B16] Chandrdas D. Jayakumar H. L. Chandra M. Katodia L. Sreedevi A. (2014). Evaluation of antimicrobial efficacy of garlic, tea tree oil, cetylpyridinium chloride, chlorhexidine, and ultraviolet sanitizing device in the decontamination of toothbrush. Indian J. Dent. 5 (4), 183–189. 10.4103/0975-962x.144718 25565751 PMC4260383

[B17] Cho Y. S. Choi Y. H. (2017). Comparison of three cooling methods for burn patients: A randomized clinical trial. Burns 43 (3), 502–508. 10.1016/j.burns.2016.09.010 27707640

[B18] Craig J. P. Bitton E. Dantam J. Jones L. Ngo W. Wang M. T. M. (2022). Short-term tolerability of commercial eyelid cleansers: A randomised crossover study. Cont. Lens Anterior Eye 45, 101733. 10.1016/j.clae.2022.101733 35842288

[B19] de Groot A. C. Schmidt E. (2016). Essential Oils, Part IV: Contact Allergy. Dermatitis 27 (4), 170–175. 10.1097/der.0000000000000197 27427818

[B20] Deyno S. Mtewa A. G. Abebe A. Hymete A. Makonnen E. Bazira J. (2019). Essential oils as topical anti-infective agents: A systematic review and meta-analysis. Complementary Ther. Med. 47, 102224. 10.1016/j.ctim.2019.102224 31780027

[B21] Down Under Enterprises (2017a). Pure Australian tea tree oil: Medicinal applications of tea tree oil. Available at: https://www.downunderenterprises.com/teatreeoil-antivirus-mechanism .

[B22] Down Under Enterprises (2016a). Pure Australian tea tree oil: Personal care applications for skin and hair. Available at: https://www.downunderenterprises.com/ .

[B23] Down Under Enterprises (2017b). Pure Australian tea tree oil: Tea tree oil in household care products. Available at: https://www.downunderenterprises.com/tto-homecare.

[B24] Down Under Enterprises (2016b). Pure Australian tea tree oil: Tea tree oil in oral care applications. Available at: https://www.downunderenterprises.com/ .

[B25] Dryden M. S. Dailly S. Crouch M. (2004). A randomized, controlled trial of tea tree topical preparations versus a standard topical regimen for the clearance of MRSA colonization. J. Hosp. Infect. 56 (4), 283–286. 10.1016/j.jhin.2004.01.008 15066738

[B26] Durić K. Kovčić Hadžiabdić S. Durić M. Nikšić H. Uzunović A. Džudžević Čančar H. (2021). Efficacy and safety of three plant extracts based formulations of vagitories in the treatment of vaginitis: a randomized controlled trial. Med. Glas. (Zenica) 18 (1), 47–54. 10.17392/1261-21 33269580

[B27] Elgendy E. A. Ali S. A. Zineldeen D. H. (2013). Effect of local application of tea tree (Melaleuca alternifolia) oil gel on long pentraxin level used as an adjunctive treatment of chronic periodontitis: A randomized controlled clinical study. J. Indian Soc. Periodontol. 17 (4), 444–448. 10.4103/0972-124x.118314 24174722 PMC3800405

[B28] Enshaieh S. Jooya A. Siadat A. H. Iraji F. (2007). The efficacy of 5% topical tea tree oil gel in mild to moderate acne vulgaris: A randomized, double-blind placebo-controlled study. Indian J. Dermatology, Venereol. Leprology 73 (1), 22–25. 10.4103/0378-6323.30646 17314442

[B29] Ernst E. Huntley A. (2000). Tea tree oil: A systematic review of randomized clinical trials. Forsch. Komplementarmedizin Klass. Naturheilkd. 7 (1), 17–20. 10.1159/000057164 10800248

[B30] European Medical Agency (2012). European Union herbal monograph on Melaleuca alternifolia (Maiden and Betch) Cheel, M. linariifolia Smith, M. dissitiflora F. Mueller and/or other species of Melaleuca, aetheroleum EMA/HMPC/320930/2012. Available at: https://www.ema.europa.eu/documents/herbal-monograph/final-european-union-herbal-monograph-melaleuca-alternifolia-maiden-betch-cheel-m-linariifolia-smith/other-species-melaleuca-aetheroleum-first-version_en.pdf .

[B31] Montreal Process Implementation Group for Australia and National Forest Inventory Steering Committee (2019). Australia’s State of the Forests Report 2018. Canberra: ABARES. Available at: https://www.awe.gov.au/abares/forestsaustralia/profiles/melaleuca-2019 (Accessed December 1, 2021 2021).

[B32] Gnatta J. R. de Brito Poveda V. Padoveze M. C. Graziano K. U. Turrini R. N. T. da Silva M. J. P. (2021). Melaleuca alternifolia essential oil soap: a potential alternative for hand hygiene. Eur. J. Clin. Microbiol. Infect. Dis. 40 (7), 1517–1520. 10.1007/s10096-021-04190-w 33635424

[B33] Gnatta J. R. Pinto F. M. Bruna C. Q. Souza R. Q. Graziano K. U. Silva M. J. (2013). Comparison of hand hygiene antimicrobial efficacy: Melaleuca alternifolia essential oil versus triclosan. Rev. Lat. Am. Enferm. 21 (6), 1212–1219. 10.1590/0104-1169.2957.2356 24402336

[B34] Groppo F. C. Ramacciato J. C. Simões R. P. Flório F. M. Sartoratto A. (2002). Antimicrobial activity of garlic, tea tree oil, and chlorhexidine against oral microorganisms. Int. Dent. J. 52 (6), 433–437. 10.1111/j.1875-595X.2002.tb00638.x 12553397

[B35] Hammer K. A. Carson C. F. Riley T. V. Nielsen J. B. (2006). A review of the toxicity of Melaleuca alternifolia (tea tree) oil. Food Chem. Toxicol. 44 (5), 616–625. 10.1016/j.fct.2005.09.001 16243420

[B36] Hawkins J. Hires C. Dunne E. Baker C. (2020). The relationship between lavender and tea tree essential oils and pediatric endocrine disorders: A systematic review of the literature. Complement. Ther. Med. 49, 102288. 10.1016/j.ctim.2019.102288 32147050

[B37] Hawkins J. Hires C. Dunne E. Keenan L. (2021). Prevalence of endocrine disorders among children exposed to Lavender Essential Oil and Tea Tree Essential Oils. Int. J. Pediatr. Adolesc. Med. 9, 117–124. 10.1016/j.ijpam.2021.10.001 35663791 PMC9152575

[B38] Henley D. V. Lipson N. Korach K. S. Bloch C. A. (2007). Prepubertal gynecomastia linked to lavender and tea tree oils. N. Engl. J. Med. 356 (5), 479–485. 10.1056/NEJMoa064725 17267908

[B39] Higgins J. P. T. Altman D. G. Gøtzsche P. C. Jüni P. Moher D. Oxman A. D. (2011). The Cochrane Collaboration’s tool for assessing risk of bias in randomised trials. BMJ 343, d5928. 10.1136/bmj.d5928 22008217 PMC3196245

[B40] Hugo Infante V. Maria Maia Campos P. Darvin M. Lohan S. Schleusener J. Schanzer S. (2023). Cosmetic Formulations with Melaleuca alternifolia Essential Oil for the Improvement of Photoaged Skin: A Double-Blind, Randomized, Placebo-Controlled Clinical Study. Photochem Photobiol 99 (1), 176–183. 10.1111/php.13660 35668682

[B41] International Organization for Standardization (2017). ISO 4730:2017(en) Essential oil of Melaleuca, terpinen-4-ol type (Tea Tree oil). 3rd ed. Vernier, Geneva. Switzerland: ISO copyright office (www.iso.org) .

[B42] Kamath N. P. Tandon S. Nayak R. Naidu S. Anand P. S. Kamath Y. S. (2020). The effect of aloe vera and tea tree oil mouthwashes on the oral health of school children. Eur. Archives Paediatr. Dent. 21 (1), 61–66. 10.1007/s40368-019-00445-5 31111439

[B43] Karakurt Y. Zeytun E. (2018). Evaluation of the Efficacy of Tea Tree Oil on the Density of Demodex Mites (Acari: Demodicidae) and Ocular Symptoms in Patients with Demodectic Blepharitis. J. Parasitol. 104 (5), 473–478. 10.1645/18-46 30016200

[B44] Koo H. Kim T. H. Kim K. W. Wee S. W. Chun Y. S. Kim J. C. (2012). Ocular surface discomfort and Demodex: effect of tea tree oil eyelid scrub in Demodex blepharitis. J. Korean Med. Sci. 27 (12), 1574–1579. 10.3346/jkms.2012.27.12.1574 23255861 PMC3524441

[B45] Lam N. S. K. Long X. X. Li X. Yang L. Griffin R. C. Doery J. C. (2020). Comparison of the efficacy of tea tree (Melaleuca alternifolia) oil with other current pharmacological management in human demodicosis: A Systematic Review. Parasitology 147 (14), 1587–1613. 10.1017/S003118202000150X 32772960 PMC10317738

[B46] Lee K. A. Harnett J. E. Cairns R. (2020). Essential oil exposures in Australia: analysis of cases reported to the NSW Poisons Information Centre. Med. J. Aust. 212 (3), 132–133. 10.5694/mja2.50403 31709543

[B47] Lee R. L. P. Leung P. H. M. Wong T. K. S. (2014). A randomized controlled trial of topical tea tree preparation for MRSA colonized wounds. Int. J. Nurs. Sci. 1 (1), 7–14. 10.1016/j.ijnss.2014.01.001

[B48] Maghu S. Desai V. D. Sharma R. (2016). Comparison of efficacy of alternative medicine with allopathy in treatment of oral fungal infection. J. Tradit. Complement. Med. 6 (1), 62–65. 10.1016/j.jtcme.2014.11.023 26870682 PMC4738066

[B49] Markum E. Baillie J. (2012). Combination of essential oil of Melaleuca alternifolia and iodine in the treatment of molluscum contagiosum in children. J. Drugs Dermatol 11 (3), 349–354. 22395586

[B50] Mohammadpour M. Maleki S. Khorrami-Nejad M. (2020). The effect of tea tree oil on dry eye treatment after phacoemulsification cataract surgery: A randomized clinical trial. Eur. J. Ophthalmol. 30 (6), 1314–1319. 10.1177/1120672119867642 31379213

[B51] Murray M. (1992). The healing power of herbs. USA: Prima Health.

[B52] Najafi-Taher R. Jafarzadeh Kohneloo A. Eslami Farsani V. Mehdizade Rayeni N. Moghimi H. R. Ehsani A. (2022). A topical gel of tea tree oil nanoemulsion containing adapalene versus adapalene marketed gel in patients with acne vulgaris: a randomized clinical trial. Arch. Dermatol Res. 314 (7), 673–679. 10.1007/s00403-021-02267-2 34251536

[B53] Ozkaraman A. Dugum O. Ozen Yilmaz H. Usta Yesilbalkan O. (2018). Aromatherapy: The Effect of Lavender on Anxiety and Sleep Quality in Patients Treated With Chemotherapy. Clin. J. Oncol. Nurs. 22 (2), 203–210. 10.1188/18.CJON.203-210 29547610

[B54] Page M. J. McKenzie J. E. Bossuyt P. M. Boutron I. Hoffmann T. C. Mulrow C. D. (2021). The PRISMA 2020 statement: an updated guideline for reporting systematic reviews. Rev. Esp. Cardiol. Engl. Ed. 74 (9), 790–799. 10.1016/j.rec.2021.07.010 34446261

[B55] Prabhakar A. R. Vipin A. H. U. J. A. Basappa N. (2009). Effect of Curry Leaves, Garlic and Tea Tree Oil on Streptococcus Mutans and Lactobacilli in Children: A Clinical and Microbiological Study. Pesqui. Bras. em Odontopediatria Clínica Integr. 9 (3), 259–263. 10.4034/1519.0501.2009.0093.0002

[B56] Rahman B. Alkawas S. Al Zubaidi E. A. Adel O. I. Hawas N. (2014). Comparative antiplaque and antigingivitis effectiveness of tea tree oil mouthwash and a cetylpyridinium chloride mouthwash: A randomized controlled crossover study. Contemp. Clin. Dent. 5 (4), 466–470. 10.4103/0976-237X.142813 25395761 PMC4229754

[B57] Raut C. P. Sethi K. S. (2016). Comparative evaluation of co-enzyme Q10 and Melaleuca alternifolia as antioxidant gels in treatment of chronic periodontitis: A clinical study. Contemp. Clin. Dent. 7 (3), 377–381. 10.4103/0976-237X.188572 27630504 PMC5004553

[B58] Reddy V. Bennadi D. Satish G. Divya G. Reddy C. V. K. (2020). Effectiveness of tea tree oil and chlorhexidine as mouth rinse in the control of dental plaque and chronic gingivitis -A comparative study. Eur. J. Mol. Clin. Med. 7 (8), 1576–1582.

[B59] Rhind J. P. (2020). “Melaleuca alternifolia - tea tree,” in Essential oils: A comprehensive handbook for aromatic therapy (United Kingdom: Singing Dragon), 488–493.

[B60] Ripari F. Cera A. Freda M. Zumbo G. Zara F. Vozza I. (2020). Tea Tree Oil versus Chlorhexidine Mouthwash in Treatment of Gingivitis: A Pilot Randomized, Double Blinded Clinical Trial. Eur. J. Dent. 14 (1), 55–62. 10.1055/s-0040-1703999 32168532 PMC7069753

[B61] Rothenberger J. Krauss S. Tschumi C. Rahmanian-Schwarz A. Schaller H. E. Held M. (2016). The Effect of Polyhexanide, Octenidine Dihydrochloride, and Tea Tree Oil as Topical Antiseptic Agents on *in vivo* Microcirculation of the Human Skin: A Noninvasive Quantitative Analysis. Wounds 28 (10), 341–346. 27768571

[B62] Salvatori C. Barchi L. Guzzo F. Gargari M. (2017). A comparative study of antibacterial and anti-inflammatory effects of mouthrinse containing tea tree oil. Oral Implantol. (Rome) 10 (1), 59–70. 10.11138/orl/2017.10.1.059 28757937 PMC5516420

[B63] Satchell A. C. Saurajen A. Bell C. Barnetson R. S. (2002a). Treatment of dandruff with 5% tea tree oil shampoo. J. Am. Acad. Dermatol 47 (6), 852–855. 10.1067/mjd.2002.122734 12451368

[B64] Satchell A. C. Saurajen A. Bell C. Barnetson R. S. (2002b). Treatment of interdigital tinea pedis with 25% and 50% tea tree oil solution: a randomized, placebo-controlled, blinded study. Australas. J. Dermatol 43 (3), 175–178. 10.1046/j.1440-0960.2002.00590.x 12121393

[B65] Savla K. Le J. T. Pucker A. D. (2020). Tea tree oil for Demodex blepharitis. Cochrane Database Syst. Rev. 6 (6), CD013333. 10.1002/14651858.CD013333.pub2 32589270 PMC7388771

[B66] Saxer U. P. Stauble A. Szabo S. H. Menghini G. (2003). Effect of mouthwashing with tea tree oil on plaque and inflammation. Schweiz Monatsschr Zahnmed. 113 (9), 985–996. 14567294

[B67] Shetty S. K. Sharath K. Shenoy S. Sreekumar C. Shetty R. N. Biju T. (2013). Compare the effcacy of two commercially available mouthrinses in reducing viable bacterial count in dental aerosol produced during ultrasonic scaling when used as a preprocedural rinse. J. Contemp. Dent. Pract. 14 (5), 848–851. 10.5005/jp-journals-10024-1414 24685786

[B68] Soukoulis S. Hirsch R. (2004). The effects of a tea tree oil-containing gel on plaque and chronic gingivitis. Aust. Dent. J. 49 (2), 78–83. 10.1111/j.1834-7819.2004.tb00054.x 15293818

[B69] Srikumar K. P. Bhagyashree B. N. Srirangarajan S. Ravi R. J. Vinaya R. (2022). Efficacy of Melaleuca alternifolia and chlorhexidine mouth rinses in reducing oral malodor and Solobacterium moorei levels. A 1 week, randomized, double-blind, parallel study. Indian J. Pharmacol. 54 (2), 77–83. 10.4103/ijp.ijp_772_20 35546457 PMC9249155

[B70] Taalab M. R. Mahmoud S. A. Moslemany R. M. E. Abdelaziz D. M. (2021). Intrapocket application of tea tree oil gel in the treatment of stage 2 periodontitis. BMC Oral Health 21 (1), 239. 10.1186/s12903-021-01588-y 33952216 PMC8101226

[B71] Thomas A. Deshmukh R. (2019). Tea tree oil market by application (cosmetics & toiletries application, therapeutics application, and industrial application), end user (FMCG manufacturer, cosmetics companies, pharmaceutical companies, and others), and grade (Pharma/Cosmetic grade and therapeutic grade): Global opportunity analysis and industry forecast, 2018–2025. Allied Market Research.)

[B72] Tisserand R. Young R. (2014). Essential oil safety: A guide for health care professionals. Edinburgh: Churchill Livingstone.

[B73] Toholka R. Wang Y. S. Tate B. Tam M. Cahill J. Palmer A. (2015). The first Australian Baseline Series: Recommendations for patch testing in suspected contact dermatitis. Australas. J. Dermatol 56 (2), 107–115. 10.1111/ajd.12186 25196101

[B74] Tong M. M. Altman P. M. Barnetson R. S. (1992). Tea tree oil in the treatment of tinea pedis. Australas. J. Dermatol 33 (3), 145–149. 10.1111/j.1440-0960.1992.tb00103.x 1303075

[B75] Wong K. Flanagan J. Jalbert I. Tan J. (2019). The effect of Blephadex Eyelid Wipes on Demodex mites, ocular microbiota, bacterial lipase and comfort: a pilot study. Cont. Lens Anterior Eye 42 (6), 652–657. 10.1016/j.clae.2019.06.001 31239200

[B76] World Health Organization (1999). WHO consultation on selected medicinal plants (2nd: 1999: Ravello-salerno, Italy). WHO monographs on selected medicinal plants. Geneva: World Health Organization.

[B77] Youn B. H. Kim Y. S. Yoo S. Hur M. H. (2021). Antimicrobial and hand hygiene effects of Tea Tree Essential Oil disinfectant: A randomised control trial. Int. J. Clin. Pract. 75 (8), e14206. 10.1111/ijcp.14206 33950544

[B78] Zarei-Ghanavati S. Nooghabi M. J. Zamani G. (2021). Comparison of the Effect of Tea Tree Oil Shampoo With Regular Eyelid Shampoo in Meibomian Gland Dysfunction Treatment. Am. J. Ophthalmol. 229, 45–51. 10.1016/j.ajo.2021.04.009 33905746

