# Supplementary material for: Efficacy and safety of Melaleuca alternifolia (tea tree) oil for human health—A systematic review of randomized controlled trials
Source: Front Pharmacol. 2023 Mar 24;14:1116077. doi: 10.3389/fphar.2023.1116077 (PMC10080088; doi:10.3389/fphar.2023.1116077)
Supplement: Supplementary file 1 [file DataSheet1.docx]

Supplementary Material

# Search strategies executed in electronic databases

**Pubmed (**[**https://pubmed.ncbi.nlm.nih.gov/advanced/**](https://pubmed.ncbi.nlm.nih.gov/advanced/)**)**

((Tea Tree Oil[Title/Abstract] OR Melaleuca alternifolia[Title/Abstract])) AND (randomized controlled trial[Publication Type] OR controlled clinical trial[Publication Type] OR randomized[Title/Abstract] OR randomised[Title/Abstract] OR random [Title/Abstract] OR randomly[Title/Abstract] OR group[Title/Abstract] OR groups[Title/Abstract] OR trial[Title/Abstract] OR control[Title/Abstract] OR controlled[Title/Abstract])

**Scopus (**[**https://www-scopus-com.ezproxy.scu.edu.au/search/form.uri?display=advanced**](https://www-scopus-com.ezproxy.scu.edu.au/search/form.uri?display=advanced)**)**

( TITLE-ABS-KEY ( tea AND tree AND oil ) OR TITLE-ABS-KEY ( melaleuca AND alternifolia ) ) AND ( TITLE-ABS-KEY ( random* ) OR TITLE-ABS ( control* ) OR TITLE-ABS-KEY ( placebo ) OR TITLE-ABS ( trial ) )

**CENTRAL Cochrane Library (**[**https://www.cochranelibrary.com/advanced-search?q=*&t=6**](https://www.cochranelibrary.com/advanced-search?q=*&t=6)**)**

MeSH descriptor: [Tea Tree Oil] explode all trees OR MeSH descriptor: [Melaleuca] explode all trees OR ("tea tree oil"):ti,ab,kw OR ("Melaleuca alternifolia"):ti,ab,kwSupplementary Material should be uploaded separately on submission. Please include any supplementary data, figures and/or tables. All supplementary files are deposited to FigShare for permanent storage and receive a DOI.
